# Synthesis, Antifungal, and Antibacterial Activities of Novel Benzoylurea Derivatives Containing a Pyrimidine Moiety

**DOI:** 10.3390/molecules28186498

**Published:** 2023-09-07

**Authors:** Jiansong An, Wenjun Lan, Qiang Fei, Pei Li, Wenneng Wu

**Affiliations:** 1 School of Food Science and Engineering, Guiyang University, Guiyang 550005, China; anjiansong027@126.com (J.A.); lwj1371801231@126.com (W.L.); fqorganic@163.com (Q.F.); 2 State Key Laboratory of Functions and Applications of Medicinal Plants, Guizhou Medical University, Guiyang 550004, China; 3 Natural Products Research Center of Guizhou Province, Guiyang 550000, China; 4 Qiandongnan Engineering and Technology Research Center for Comprehensive Utilization of National Medicine, Kaili University, Kaili 556011, China

**Keywords:** benzoylurea, pyrimidine, antifungal activity, antibacterial activity, succinate dehydrogenase

## Abstract

To explore more efficient and less toxic antibacterial and antifungal pesticides, we utilized 2,6-difluorobenzamide as a starting material and ultimately synthesized 23 novel benzoylurea derivatives containing a pyrimidine moiety. Their structures were characterized and confirmed by ^1^H NMR, ^13^C NMR, ^19^F NMR, and HRMS. The bioassay results demonstrated that some of the title compounds exhibited moderate to good in vitro antifungal activities against *Botrytis cinerea* in cucumber, *Botrytis cinerea* in tobacco, *Botrytis cinerea* in blueberry, *Phomopsis* sp., and *Rhizoctonia solani*. Notably, compounds **4j** and **4l** displayed EC_50_ values of 6.72 and 5.21 μg/mL against *Rhizoctonia solani*, respectively, which were comparable to that of hymexazol (6.11 μg/mL). Meanwhile, at 200 and 100 concentrations, the target compounds **4a**–**4w** exhibited lower in vitro antibacterial activities against *Xanthomonas oryzae* pv. *oryzicola* and *Xanthomonas citri* subsp. *citri*, respectively, compared to those of thiodiazole copper. Furthermore, the molecular docking simulation demonstrated that compound **4l** formed hydrogen bonds with SER-17 and SER-39 of succinate dehydrogenase (SDH), providing a possible explanation for the mechanism of action between the target compounds and SDH. This study represents the first report on the antifungal and antibacterial activities of novel benzoylurea derivatives containing a pyrimidine moiety.

## 1. Introduction

Pesticides serve as a primary means of preventing and controlling agricultural disasters, such as pests and weeds, thereby ensuring the healthy growth and successful harvest of crops [1,2,3]. For a prolonged period, crop yields have been constrained not only by natural conditions but also by detrimental biological infestations, particularly fungal and bacterial diseases that have exhibited an exacerbating trend in recent years, jeopardizing the stable production of crops [4,5]. Although China’s current grain production is sufficient to meet basic needs, the significant amount of money spent annually on pest control highlights ongoing challenges in this area. While farm chemicals play a crucial role in disease management, their non-standard use can have adverse effects on ecosystems. The issue of pesticide misuse has resulted in problems such as excessive residues in crops, resistance to pesticides, and pollution of water and soil. Therefore, it is imperative to develop novel pesticides with enhanced biological activity, broad-spectrum sterilization capabilities, greater efficiency, and reduced toxicity.

**Figure 1:**
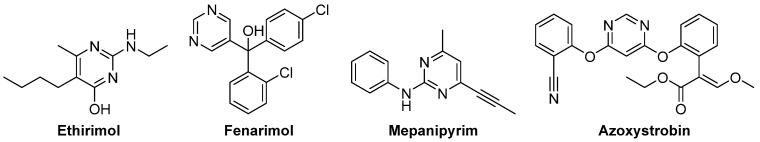
Some commercial pesticides containing a pyrimidine structure.

**Figure 2:**
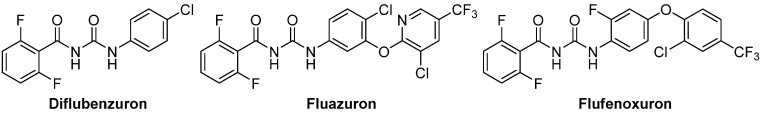
Some commercial pesticides containing a benzoylurea structure.

Pyrimidine compounds have demonstrated a diverse range of biological activities in previous studies, encompassing antiviral, antibacterial, fungicidal, insecticidal, and herbicidal properties [6,7,8,9,10,11,12,13,14,15,16,17,18,19,20]. Consequently, a variety of pyrimidine derivatives (Figure 1) have been successfully developed into commercial pesticides that have made significant contributions to the smooth functioning of agricultural production. Pyrimidine derivatives have exhibited promising potential as an initial foundation for the exploration of novel succinate dehydrogenase inhibitors (SDHIs) [21,22]. Meanwhile, previous research has demonstrated the effective insecticidal properties of benzoylureas, which are commercially available as diflubenzuron, fluazuron, and flufenoxuron (Figure 2) [23,24,25]. These compounds offer numerous advantages including mild environmental impact, low residue levels, and easy degradation, making them a popular focus in synthetic research for an extended period. As potent inhibitors of chitin synthesis, benzoylureas exert a remarkably active inhibitory effect owing to their distinctive mechanism of action. Consequently, research on benzoylurea compounds in the field of insecticidal and acaricidal properties has reached a relatively advanced stage of development. Recent studies also have revealed that benzoylurea compounds possess certain fungicidal activity [26]. In addition, the chemistry of fluorine-containing compounds has undergone significant advancements in recent years. Inherent characteristics of the fluorine atom, such as its high electronegativity, small atomic radius, and low polarizability of the C–F bond, contribute significantly to enhancing the biological activity of fluorinated molecules [27,28]. Thus, the substitution of fluorine continues to be an appealing strategy in the advancement of drug molecules with enhanced activity and selectivity.

The present study drew inspiration from the aforementioned research and employed the principle of molecular hybridization to strategically combine benzoylurea with a pyrimidine moiety, resulting in the design and synthesis of a series of novel benzoylurea derivatives featuring an active pyrimidine group. Then, their in vitro antibacterial and antifungal activities were determined. Finally, a molecular docking study was conducted to investigate the binding mode of the target compounds with succinate dehydrogenase (SDH).

## 2. Results and Discussion

### 2.1. Chemistry

**Scheme 1:**
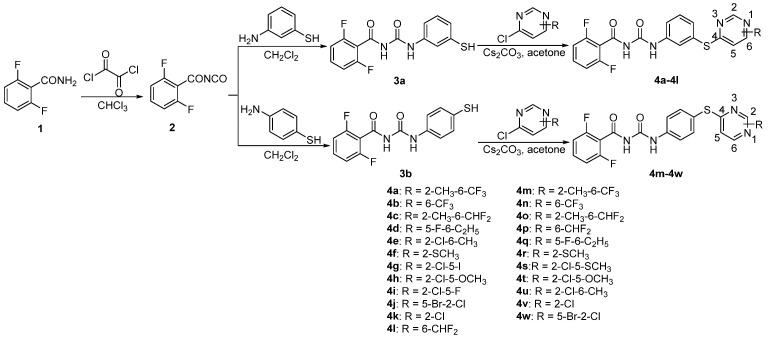
The synthetic procedures of benzoylurea derivatives incorporating a pyrimidine moiety.

The synthetic procedures of the target compounds **4a**–**4w** were summarized in Scheme 1. As shown in Scheme 1, using 2,6-difluorobenzamide as the starting material, the target compounds were prepared by condensation, acylation, and thioetherification reactions with the yields of 37.3–97.5%. The structures were confirmed by ^1^H NMR, ^13^C NMR, ^19^F NMR, and HRMS.

In the ^1^H NMR spectra of compound **4l**, two singlet peaks at 10.64 and 9.06 ppm verified the presence of two CONH groups; one multiplet at 7.12–7.09 ppm indicated the presence of H atoms in the pyrimidine structure. In the ^13^C NMR spectra of compound **4l**, two singlet peaks at 174.84 and 162.18 ppm revealed the presence of C atoms in the C=O group; one triplet at 133.99 ppm indicated the presence of C atoms in the CHF_2_ group. In addition, the molecular weight of compound **4l** was correctly assigned by HRMS data with the [M + Na]^+^ ions of *m/z* 459.05093.

### 2.2. Biological Evaluations

The in vitro antifungal activities of the target compounds **4a**–**4w** against *Botrytis cinerea* in cucumber, *Botrytis cinerea* in tobacco, *Botrytis cinerea* in blueberry, *Phomopsis* sp., and *Rhizoctonia solani* were evaluated using the mycelial growth rate method, and the preliminary bioassay results are listed in Table 1. Table 1 demonstrates that the target compounds exhibit certain in vitro antifungal activities against *Botrytis cinerea* in cucumber (3.65–50.15%), *Rhizoctonia solani* (40.88–89.74%), *Botrytis cinerea* in tobacco (35.74–51.26%), *Phomopsis* sp. (21.73–49.84%), and *Botrytis cinerea* in blueberry (2.83–62.26%). Among them, compounds **4f**, **4l**, and **4q** demonstrate significant in vitro antifungal activity against *Botrytis cinerea* in cucumber at 50 μg/mL, with the inhibition rates of 32.52%, 50.15%, and 43.47%, respectively, which surpass the efficacy of hymexazol (24.64%). Furthermore, compound **4l** exhibits superior antifungal activity (89.74%) against *Rhizoctonia solani* compared to hymexazol (71.98%). Additionally, compound **4j** demonstrates comparable antifungal activity (49.84%) against *Phomopsis* sp., with an efficacy equivalent to that of hymexazol (47.09%).

Meanwhile, the 50% effective concentration (EC_50_) values of compounds **4j** and **4l** against *Rhizoctonia solani* were also determined and are listed in Table 2. Table 2 shows that the EC_50_ values of compounds **4j** and **4l** against *Rhizoctonia solani* were 6.72 and 5.21 μg/mL, respectively, which were similar to that of hymexazol (6.11 μg/mL).

In addition, the in vitro antibacterial activities of the target compounds **4a**–**4w** against *Xanthomonas oryzae* pv. *oryzicola* and *Xanthomonas citri* subsp. *citri* were assessed using the turbidimeter tests, and the preliminary bioassay results are presented in Table 3. As shown in Table 3, all the test compounds exhibit lower antibacterial activities against *Xanthomonas oryzae* pv. *oryzicola* and *Xanthomonas citri* subsp. *citri* compared to those of thiodiazole copper at concentrations of 200 and 100 μg/mL.

### 2.3. Docking Analysis

**Figure 3:**
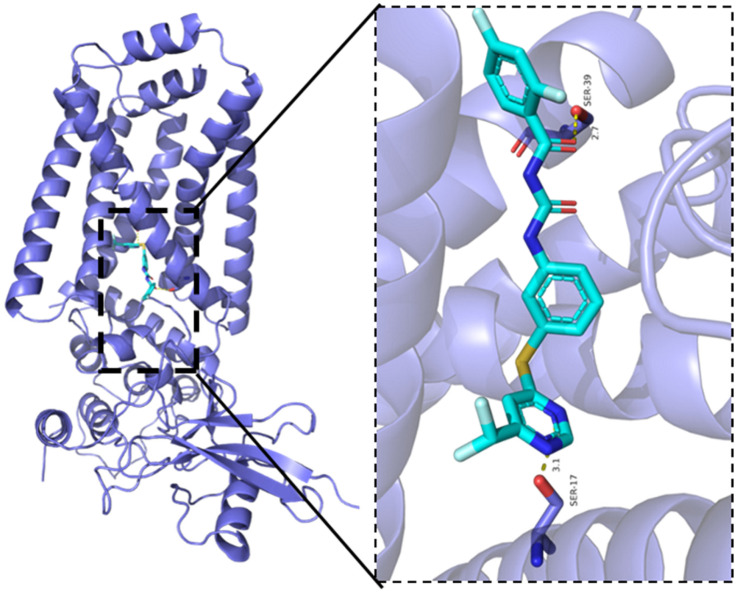
The docking binding mode of compound **4l** to SDH.

To elucidate the binding mode of the target compounds to SDH, a molecular docking simulation was conducted for compound **4l** and SDH. As depicted in Figure 3, compound **4l** was successfully docked into the active site of the SDH receptor (PDB: 2FBW) with a favorable binding energy of −10.6 kcal/mol. Notably, the N atom in the pyrimidine ring formed a hydrogen bond interaction with the amino acid residue SER-17 at a distance of 3.1 Å, while the O atom in the amide group established another hydrogen bond interaction with the amino acid residue SER-39 at a distance of 2.7 Å.

## 3. Materials and Methods

### 3.1. Materials and Instruments

The melting points (m.p.) of the target compounds were determined on an uncorrected XT-4 binocular microscope (Beijing Tech Instrument Co., Beijing, China). Nuclear magnetic resonance (^1^H NMR, ^13^C NMR, and ^19^F NMR) was conducted on a Bruker NMR spectrometer (Bruker, Rheinstetten, Germany). High resolution mass spectrometry (HRMS) was performed on a Thermo Scientic Q Exactive Plus instrument (Thermo Fisher Scientific, Waltham, MA, USA).

### 3.2. Chemical Synthesis

#### 3.2.1. Preparation Procedure of Intermediates **2**–**3**

As shown in Scheme 1, 2,6-difluorobenzamide (20 mmol) and CHCl_3_ (20 mL) were added to a three-necked flask (50 mL) equipped with a tail gas treatment device (0.25 mol/L NaOH aqueous solution, 200 mL) on the condenser tube. Oxalyl chloride (40 mmol), dissolved in CHCl_3_ (20 mL), was then carefully added dropwise. The mixture was stirred for 0.5 h under ice bath conditions before being placed in an oil bath at 65 °C for reflux condensation reaction. After the completion of the reaction, the mixture was dried under pressure to obtain intermediates **2**.

The 3-aminothiophenol or 4-aminothiophenol (20 mmol) and CH_2_Cl_2_ (40 mL) were added to a 100 mL three-neck flask, followed by the slow dropwise addition of intermediate **2** (21 mmol) which was dissolved in CH_2_Cl_2_ (15 mL). The reaction mixture was allowed to proceed at room temperature. Upon completion of the reaction, the resulting solid precipitate was filtered, washed with methanol, dried, and subjected to recrystallization using anhydrous methanol to obtain intermediates **3**.

#### 3.2.2. Preparation Procedure of the Target Compounds **4a**–**4w**

As shown in Scheme 1, intermediate **3** (10 mmol), acetone (20 mL), Cs_2_CO_3_ (15 mmol), and substituted 4-chloropyrimidine (11 mmol) were added to a 50 mL round bottom flask and reacted at room temperature. Upon completion of the reaction, the resulting solid precipitate was filtered, washed with methanol, dried, and purified using column chromatography to obtain the target compounds **4a**–**4w**. The physical characteristics and the ^1^H NMR, ^13^C NMR, ^19^F NMR, and HRMS data of the target compounds **4a**–**4w** are shown below. The spectra of ^1^H NMR, ^13^C NMR, ^19^F NMR, and HRMS for compounds **4a**–**4w** are shown in Supplementary Materials.

2,6-Difluoro-*N*-((3-(((2-methyl-6-(trifluoromethyl)pyrimidin-4-yl)thio)phenyl)carbamoyl) benzamide (**4a**). White solid; yield 49.5%; m.p. 149.4–152.7 °C; ^1^H NMR (600 MHz, DMSO-*d*_6_, ppm) *δ* 11.56 (s, 1H, CONH), 10.40 (s, 1H, CONH), 8.02 (t, *J* = 2.00 Hz, 1H, Ph-H), 7.78 (d, *J* = 7.20 Hz, 1H, Ph-H), 7.67–7.32 (m, 1H, Ph-H), 7.57 (t, *J* = 7.90 Hz, 1H, Ph-H), 7.45–7.44 (m, 1H, Ph-H), 7.27 (t, *J* = 8.20 Hz, 2H, Ph-H), 7.19 (s, 1H, pyrimidine-H), 2.64 (s, 3H, pyrimidine-CH_3_); ^13^C NMR (150 MHz, DMSO-*d*_6_, ppm) *δ* 175.09, 168.69, 162.63, 160.02 (d, *J* = 6.60 Hz), 158.36 (d, *J* = 7.20 Hz), 154.07 (q, *J* = 34.67 Hz), 150.63, 139.44, 133.65, 131.16, 130.98, 127.17, 126.76, 123.64 (q, *J* = 274.13 Hz), 122.73, 112.64 (d, *J* = 20.55 Hz), 112.61 (d, *J* = 20.70 Hz), 110.46 (d, *J* = 2.77 Hz), 25.78; ^19^F NMR (565 MHz, DMSO-*d*_6_, ppm) δ −69.10, −113.49; HRMS (ESI) *m/z* calculated for C_20_H_13_F_5_N_4_O_2_S [M + Na]^+^: 491.05695, found: 491.05716.
2,6-Difluoro-*N*-((3-(((6-(trifluoromethyl)pyrimidin-4-yl)thio)phenyl)carbamoyl) benzamide (**4b**). White solid; yield 52.2%; m.p. 150.1–153.4 °C; ^1^H NMR (600 MHz, CDCl_3_, ppm) *δ* 10.63 (s, 1H, CONH), 9.66 (s, 1H, CONH), 8.97 (s, 1H, pyrimidine-H), 7.78 (s, 1H, Ph-H), 7.48 (d, *J* = 8.30 Hz, 1H, Ph-H), 7.40 (t, *J* = 8.10 Hz, 2H, Ph-H), 7.30 (d, *J* = 7.60 Hz, 1H, Ph-H), 7.05 (s, 1H, pyrimidine-H), 6.93 (t, *J* = 8.70 Hz, 2H, Ph-H); ^13^C NMR (150 MHz, CDCl_3_, ppm) *δ* 175.60, 162.53, 161.02 (d, *J* = 7.35 Hz), 158.98 (d, *J* = 6.75 Hz), 158.48, 155.03 (q, *J* = 43.50 Hz), 151.18, 138.69, 133.75 (t, *J* = 12.60 Hz), 131.49, 130.74, 127.17, 126.73, 122.47, 112.99, 112.45 (d, *J* = 25.20 Hz), 112.35 (d, *J* = 30.60 Hz), 112.21, 112.07; ^19^F NMR (565 MHz, DMSO-*d*_6_, ppm) δ −69.05, −113.42; HRMS(ESI) *m/z* calculated for C_19_H_11_F_5_N_4_O_2_S [M + Na]^+^: 477.04163, found: 477.04151.
*N*-(3-(((6-(difluoromethyl)-2-methylpyrimidin-4-yl)thio)phenyl)carbamoyl)-2,6-difluorobenzamide (**4c**). White solid; yield 59.0%; m.p. 162.1–165.4 °C; ^1^H NMR (600 MHz, CDCl_3_, ppm) *δ* 10.70 (s, 1H, CONH), 9.94 (s, 1H, CONH), 7.84 (d, *J* = 2.40 Hz, 1H, Ph-H), 7.51 (d, *J* = 8.30 Hz, 1H, Ph-H), 7.48–7.40 (m, 2H, Ph-H), 7.36 (d, *J* = 7.60 Hz, 1H, Ph-H), 6.99 (t, *J* = 8.60 Hz, 2H, Ph-H), 6.79 (s, 1H, pyrimidine-H), 6.37 (t, *J* = 54.90 Hz, 1H, pyrimidine-CHF_2_), 2.71 (s, 3H, pyrimidine-CH_3_); ^13^C NMR (150 MHz, CDCl_3_, ppm) *δ* 175.06, 168.09, 162.67, 160.95 (d, *J* = 7.35 Hz), 159.42 (t, *J* = 30.75 Hz), 158.91 (d, *J* = 7.35 Hz), 151.44, 138.56, 133.70 (t, *J* = 12.30 Hz), 131.59, 130.59, 128.00, 126.70, 122.17, 114.26, 112.46, 112.36 (d, *J* = 25.65 Hz), 112.33 (d, *J* = 25.65 Hz), 110.40, 109.45 (t, *J* = 4.35 Hz), 25.72; ^19^F NMR (565 MHz, DMSO-*d*_6_, ppm) δ −113.38, −120.61; HRMS (ESI) *m/z* calculated for C_20_H_14_F_4_N_4_O_2_S [M + Na]^+^: 473.06662, found: 473.06658.
*N*-(3-(((6-ethyl-5-fluoropyrimidin-4-yl)thio)phenyl)carbamoyl)-2,6-difluorobenzamide (**4d**). White solid; yield 54.4%; m.p. 165.2–167.9 °C; ^1^H NMR (600 MHz, CDCl_3_, ppm) *δ* 10.62 (s, 1H, CONH), 10.04 (s, 1H, CONH), 8.55 (d, *J* = 2.20 Hz, 1H, pyrimidine-H), 7.73 (t, *J* = 1.90 Hz, 1H, Ph-H), 7.49 (d, *J* = 7.60 Hz, 1H, Ph-H), 7.46 (m, 1H, Ph-H), 7.41–7.31 (m, 2H, Ph-H), 7.00 (t, *J* = 8.60 Hz, 2H, Ph-H), 2.85 (m, 2H, pyrimidine-CH_2_), 1.33 (t, *J* = 7.60 Hz, 3H, CH_3_); ^13^C NMR (150 MHz, CDCl_3_, ppm) *δ* 162.59, 160.96 (d, *J* = 7.20 Hz), 158.93 (d, *J* = 7.67 Hz), 153.48, 153.48 (t, *J* = 10.97 Hz), 151.38 (d, *J* = 8.78 Hz), 137.84, 133.53 (t, *J* = 12.06 Hz), 131.82, 129.75, 127.10, 126.93, 121.65, 112.45 (t, *J* = 21.96 Hz), 112.38 (d, *J* = 25.22 Hz), 24.07, 11.80; ^19^F NMR (565 MHz, DMSO-*d*_6_, ppm) δ −113.44, −135.12; HRMS (ESI) *m/z* calculated for C_20_H_15_F_3_N_4_O_2_S [M + Na]^+^: 455.07587, found: 455.07600.
*N*-(3-(((2-chloro-6-methylpyrimidin-4-yl)thio)phenyl)carbamoyl)-2,6-difluorobenzamide (**4e**). White solid; yield 56.6%; m.p. 201.1–203.9 °C; ^1^H NMR (600 MHz, CDCl_3_, ppm) *δ* 10.70 (s, 1H, CONH), 9.77 (s, 1H, CONH), 7.85 (t, *J* = 2.00 Hz, 1H, Ph-H), 7.54–7.48 (m, 2H, Ph-H), 7.45 (t, *J* = 7.90 Hz, 1H, Ph-H), 7.37 (d, *J* = 7.60 Hz, 1H, Ph-H), 7.02 (t, *J* = 8.60 Hz, 2H, Ph-H), 6.46 (s, 1H, pyrimidine-H), 2.35 (s, 3H, pyrimidine-CH_3_); ^13^C NMR (150 MHz, CDCl_3_, ppm) *δ* 175.64, 169.29, 162.57, 160.99 (d, *J* = 6.59 Hz), 160.13, 158.96 (d, *J* = 6.59 Hz), 151.16, 138.57, 134.08 (t, *J* = 12.06 Hz), 131.69, 130.68, 127.98, 126.81,122.27, 114.34, 112.49 (d, *J* = 25.23 Hz), 112.46 (d, *J* = 25.22 Hz), 112.11, 24.02; ^19^F NMR (565 MHz, DMSO-*d*_6_, ppm) δ −114.30; HRMS (ESI) *m/z* calculated for C_19_H_13_ClF_2_N_4_O_2_S [M + Na]^+^: 457.03036, found: 457.03080.
2,6-Difluoro-*N*-((3-(((2-(methylthio)pyrimidin-4-yl)thio)phenyl)carbamoyl)benzamide (**4f**). White solid; yield 51.8%; m.p. 232.2–234.9 °C; ^1^H NMR (600 MHz, CDCl_3_, ppm) *δ* 10.65 (s, 1H, CONH), 9.66 (s, 1H, CONH), 8.14 (d, *J* = 5.30 Hz, 1H, pyrimidine-H), 7.89–7.75 (m, 1H, Ph-H), 7.51–7.45 (m, 2H, Ph-H), 7.41 (t, *J* = 7.80 Hz, 1H, Ph-H), 7.37–7.35 (m, 1H, Ph-H), 7.01 (t, *J* = 10.02 Hz, 2H, Ph-H), 6.46–6.45 (m, 1H, pyrimidine-H), 2.44 (s, 3H, pyrimidine-SCH_3_); ^13^C NMR (150 MHz, CDCl_3_, ppm) *δ* 172.52, 172.22, 162.69, 160.93 (d, *J* = 6.59 Hz), 158.90 (d, *J* = 7.68 Hz), 155.82, 151.36, 138.21, 133.83 (t, *J* = 12.08 Hz), 131.91, 130.20, 128.55, 126.96, 121.83, 112.38 (d, *J* = 29.60 Hz), 112.35 (d, *J* = 29.61 Hz), 112.25, 112.13, 14.06; ^19^F NMR (565 MHz, DMSO-*d*_6_, ppm) δ −113.52; HRMS (ESI) *m/z* calculated for C_19_H_14_F_2_N_4_O_2_S_2_ [M + Na]^+^: 455.04172, found: 455.04184.
*N*-(3-(((2-chloro-5-iodipyrimidin-4-yl)thio)phenyl)carbamoyl)-2,6-difluorobenzamide (**4g**). Yellow solid; yield 55.0%; m.p. 192.4–195.1 °C; ^1^H NMR (600 MHz, CDCl_3_, ppm) *δ* 10.57 (s, 1H, CONH), 8.83 (s, 1H, CONH), 8.52 (s, 1H, pyrimidine-H), 7.81 (t, *J* =1.90 Hz, 1H, Ph-H), 7.61–7.59 (m, 1H, Ph-H), 7.55–7.50 (m, 1H, Ph-H), 7.44 (t, *J* = 8.00 Hz, 1H, Ph-H), 7.31 (d, *J* = 8.00 Hz, 1H, Ph-H), 7.05 (t, *J* = 8.80 Hz, 2H, Ph-H); ^13^C NMR (150 MHz, CDCl_3_, ppm) *δ* 175.07, 163.61, 162.15, 160.26, 159.09, 150.55, 137.96, 133.87, 131.51, 129.96, 128.65, 126.53, 121.99, 112.62 (d, *J* = 25.35 Hz), 112.59 (d, *J* = 24.98 Hz), 112.08, 89.01; ^19^F NMR (565 MHz, DMSO-*d*_6_, ppm) δ −114.16; HRMS (ESI) *m/z* calculated for C_18_H_10_ClF_2_IN_4_O_2_S [M + Na]^+^: 568.91199, found: 568.91179.
*N*-(3-(((2-chloro-5-methoxypyrimidin-4-yl)thio)phenyl)carbamoyl)-2,6-difluorobenzamide (**4h**). White solid; yield 42.2%; m.p. 167.6–170.4 °C; ^1^H NMR (600 MHz, CDCl_3_, ppm) *δ* 10.58 (s, 1H, CONH), 9.61 (s, 1H, CONH), 7.90 (s, 1H, pyrimidine-H), 7.71 (t, *J* = 1.90 Hz, 1H, Ph-H), 7.55 (d, *J* = 9.18 Hz, 1H, Ph-H), 7.49–7.44 (m, 1H, Ph-H), 7.39 (t, *J* = 7.90 Hz, 1H, Ph-H), 7.32 (d, *J* = 7.80 Hz, 1H, Ph-H), 7.01 (t, *J* = 8.70 Hz, 2H, Ph-H), 4.02 (s, 3H, pyrimidine-OCH_3_); ^13^C NMR (150 MHz, CDCl_3_, ppm) *δ* 162.58, 162.45, 161.00 (d, *J* = 7.68 Hz), 158.97 (d, *J* = 6.59 Hz), 151.76, 151.14, 148.75, 137.74, 136.64, 133.59 (t, *J* = 12.06 Hz), 131.81, 129.68, 127.15, 126.71, 121.63, 112.46 (d, *J* = 25.22 Hz), 112.42 (d, *J* = 25.23 Hz), 112.36, 56.77; ^19^F NMR (565 MHz, DMSO-*d*_6_, ppm) δ −113.43; HRMS (ESI) *m/z* calculated for C_19_H_13_ClF_2_N_4_O_3_S [M + Na]^+^: 473.02551, found: 473.02572.
*N*-(3-(((2-chloro-5-fluoropyrimidin-4-yl)thio)phenyl)carbamoyl)-2,6-difluorobenzamide (**4i**). Yellow solid; yield 40.9%; m.p. 170.1–173.9 °C; ^1^H NMR (600 MHz, CDCl_3_, ppm) *δ* 10.60 (s, 1H, CONH), 9.37 (s, 1H, CONH), 8.18 (s, 1H, pyrimidine-H), 7.80 (s, 1H, Ph-H), 7.58 (d, *J* = 8.10 Hz, 1H, Ph-H), 7.53–7.48 (m, 1H, Ph-H), 7.43 (t, *J* = 7.90 Hz, 1H, Ph-H), 7.34 (d, *J* = 7.80 Hz, 1H, Ph-H), 7.04 (t, *J* = 8.60 Hz, 2H, Ph-H); ^13^C NMR (150 MHz, CDCl_3_, ppm) *δ* 174.79, 164.88, 162.57, 159.99 (d, *J* = 7.50 Hz), 159.35, 158.33 (d, *J* = 7.50 Hz), 150.49, 138.90, 131.21, 130.59, 128.66, 126.46, 122.30, 112.70 (d, *J* = 22.50 Hz), 112.67 (d, *J* = 22.25 Hz), 91.87; ^19^F NMR (565 MHz, DMSO-*d*_6_, ppm) δ −113.41, −137.62; HRMS (ESI) *m/z* calculated for C_18_H_10_ClF_3_N_4_O_2_S [M + Na]^+^: 464.99000, found: 464.99625.
*N*-(3-(((5-bromo-2-chloropyrimidin-4-yl)thio)phenyl)carbamoyl)-2,6-difluorobenzamide (**4j**). Yellow solid; yield 51.6%; m.p. 136.8–140.2 °C; ^1^H NMR (600 MHz, CDCl_3_, ppm) *δ* 10.61 (s, 1H, CONH), 9.44 (s, 1H, CONH), 8.38 (s, 1H, pyrimidine-H), 7.77 (t, *J* = 2.00 Hz, 1H, Ph-H), 7.56–7.54 (m, 1H, Ph-H), 7.52–7.48 (m, 1H, Ph-H), 7.43 (t, *J* = 7.90 Hz, 1H, Ph-H), 7.32 (d, *J* = 7.70 Hz, 1H, Ph-H), 7.03 (t, *J* = 8.50 Hz, 2H, Ph-H); ^13^C NMR (150 MHz, CDCl_3_, ppm) *δ* 172.13, 162.33, 161.07 (d, *J* = 7.68 Hz), 159.08 (d, *J* = 5.48 Hz), 157.85, 150.86, 137.96, 133.74 (t, *J* = 12.06 Hz), 131.68, 129.92, 127.32, 126.62, 122.02, 115.48, 112.54 (d, *J* = 25.23 Hz), 112.50 (d, *J* = 25.22 Hz), 112.22; ^19^F NMR (565 MHz, DMSO-*d*_6_, ppm) δ −113.59; HRMS (ESI) *m/z* calculated for C_18_H_10_BrClF_2_N_4_O_2_S [M + Na]^+^: 522.92566, found: 522.92273.
*N*-(3-(((2-chloropyrimidin-4-yl)thio)phenyl)carbamoyl)-2,6-difluorobenzamide (**4k**). Yellow solid; yield 50.3%; m.p. 164.3–167.9 °C; ^1^H NMR (600 MHz, CDCl_3_, ppm) *δ* 10.70 (s, 1H, CONH), 9.70 (s, 1H, CONH), 8.21 (d, *J* = 5.50 Hz, 1H, pyrimidine-H), 7.87 (t, *J* = 2.00 Hz, 1H, Ph-H), 7.54–7.49 (m, 2H, Ph-H), 7.46 (t, *J* = 7.90 Hz, 1H, Ph-H), 7.38–7.36 (m, 1H, Ph-H), 7.02 (t, *J* = 8.60 Hz, 2H, Ph-H), 6.64 (d, *J* = 5.50 Hz, 1H, pyrimidine-H); ^13^C NMR (150 MHz, CDCl_3_, ppm) *δ* 176.40, 162.46, 161.04 (d, *J* = 6.59 Hz), 159.01 (d, *J* =7.67 Hz), 160.64, 157.93, 150.94, 138.67, 134.01 (t, *J* = 12.05 Hz), 131.59, 130.75, 127.83, 126.76, 122.36, 115.30, 112.53 (d, *J* = 25.22 Hz), 112.49 (d, *J* = 25.22 Hz), 112.05; ^19^F NMR (565 MHz, DMSO-*d*_6_, ppm) δ −113.49; HRMS (ESI) *m/z* calculated for C_18_H_11_ClF_2_N_4_O_2_S [M + Na]^+^: 443.01498, found: 443.01515.
*N*-(3-(((6-(difluoromethyl)pyrimidin-4-yl)thio)phenyl)carbamoyl)-2,6-difluorobenzamide (**4l**). Yellow solid; yield 51.6%; m.p. 121.4–124.7 °C; ^1^H NMR (600 MHz, CDCl_3_, ppm) *δ* 10.64 (s, 1H, CONH), 9.06 (s, 1H, CONH), 8.97 (s, 1H, pyrimidine-H), 7.89 (t, *J* = 1.90 Hz, 1H, Ph-H), 7.62–7.60 (m, 1H, Ph-H), 7.53–7.46 (m, 2H, Ph-H), 7.39–7.37 (m, 1H, Ph-H), 7.10 (s, 1H, pyrimidine-H), 7.05–7.02 (m, 2H, Ph-H), 6.43 (t, *J* = 54.80 Hz, 1H, pyrimidine-CHF_2_); ^13^C NMR (150 MHz, CDCl_3_, ppm) *δ* 174.84, 162.18, 161.14 (d, *J* = 7.68 Hz), 159.06 (d, *J* = 7.62 Hz), 158.11, 150.48, 138.59, 133.99 (t, *J* = 12.06 Hz), 131.53, 130.70,127.70, 126.85, 122.32, 114.11, 112.96, 112.65 (d, *J* = 26.3 Hz), 112.61 (d, *J* = 25.23 Hz), 112.18; ^19^F NMR (565 MHz, DMSO-*d*_6_, ppm) δ −113.39, −120.70; HRMS (ESI) *m/z* calculated for C_19_H_12_F_4_N_4_O_2_S [M + Na]^+^: 459.05072, found: 459.05093.
2,6-Difluoro-*N*-((4-(((2-methyl-6-(trifluoromethyl)pyrimidin-4-yl)thio)phenyl)carbamoyl) benzamide (**4m**). White solid; yield 52.5%; m.p. 159.8–162.9 °C; ^1^H NMR (600 MHz, CDCl_3_, ppm) *δ* 10.71 (s, 1H, CONH), 9.17 (s, 1H, CONH), 7.68–7.66 (m, 2H, Ph-H), 7.56–7.51 (m, 3H, Ph-H), 7.07 (t, *J* = 8.50 Hz, 2H, Ph-H), 6.83 (s, 1H, pyrimidine-H), 2.72 (s, 3H, pyrimidine-CH_3_); ^13^C NMR (150 MHz, CDCl_3_, ppm) *δ* 176.15, 168.73, 162.38, 161.12 (d, *J* = 6.75 Hz), 159.09 (d, *J* = 7.05 Hz), 154.93 (q, *J* = 42.90 Hz), 150.97, 139.38, 136.62, 133.69 (t, *J* = 12.08 Hz), 121.70, 121.50, 112.54 (d, *J* = 25.05 Hz), 112.51 (d, *J* = 25.20 Hz), 109.45, 25.81; ^19^F NMR (565 MHz, DMSO-*d*_6_, ppm) δ −69.11, −113.43; HRMS (ESI) *m/z* calculated for C_20_H_13_F_5_N_4_O_2_S [M + Na]^+^: 491.05713, found: 491.05716.
2,6-Difluoro-*N*-((4-(((6-(trifluoromethyl)pyrimidin-4-yl)thio)phenyl)carbamoyl) benzamide (**4n**).White solid; yield 51.2%; m.p. 181.6–184.9 °C; ^1^H NMR (600 MHz, CDCl_3_, ppm) *δ* 10.71 (s, 1H, CONH), 9.04 (s, 1H, CONH), 9.04–9.00 (m, 1H, pyrimidine-H), 7.69 (d, *J* = 8.50 Hz, 2H, Ph-H), 7.56 (d, *J* = 8.50 Hz, 2H, Ph-H), 7.26 (s, 1H, pyrimidine-H), 7.12 (d, *J* = 1.40 Hz, 1H, Ph-H), 7.07 (t, *J* = 10.20 Hz, 2H, Ph-H), ^13^C NMR (150 MHz, CDCl_3_, ppm) *δ* 176.08, 162.16, 161.20 (q, *J* = 6.00 Hz), 158.45, 154.63, 150.43, 139.52, 136.68, 133.96 (t, *J* = 12.30 Hz), 121.57, 121.26, 112.90 (d, *J* = 30.9 Hz), 112.88 (d, *J* = 31.8 Hz), 112.52 (d, *J* = 3.65 Hz); ^19^F NMR (565 MHz, DMSO-*d*_6_, ppm) δ −69.04, −113.38; HRMS (ESI) *m/z* calculated for C_19_H_11_F_5_N_4_O_2_S [M + Na]^+^: 477.04166, found: 477.04151.
*N*-(4-(((6-(difluoromethyl)-2-methylpyrimidin-4-yl)thio)phenyl)carbamoyl)-2,6-difluorobenzamide (**4o**). White solid; yield 56.4%; m.p. 210.5–213.8 °C; ^1^H NMR (600 MHz, CDCl_3_, ppm) *δ* 10.70 (s, 1H, CONH), 9.44 (s, 1H, CONH), 7.64 (d, *J* = 6.40 Hz, 1H, Ph-H), 7.58–7.52 (m, 3H, Ph-H), 7.27 (d, 1H, Ph-H), 7.06 (t, *J* = 8.30 Hz, 2H, Ph-H), 6.79 (s, 1H, pyrimidine-H), 6.37 (t, *J* = 54.90 Hz, 1H, pyrimidine-CHF_2_), 2.69 (s, 3H, pyrimidine-CH_3_); ^13^C NMR (150 MHz, CDCl_3_, ppm) *δ* 175.56, 168.05, 162.20, 161.17 (d, *J* = 5.56 Hz), 159.13 (d, *J* = 5.57 Hz), 150.55, 139.24, 136.70, 133.88 (t, *J* = 11.85 Hz), 122.15, 121.47,114.33, 112.65 (d, *J* = 26.85 Hz), 112.62 (d, *J* = 32.55 Hz), 109.29, 25.72; ^19^F NMR (565 MHz, DMSO-*d*_6_, ppm) δ −113.67, −120.58; HRMS (ESI) *m/z* calculated for C_20_H_14_F_4_N_4_O_2_S [M + Na]^+^: 473.06671, found: 473.06658.
*N*-(4-(((6-(difluoromethyl)pyrimidin-4-yl)thio)phenyl)carbamoyl)-2,6-difluorobenzamide (**4p**). White solid; yield 50.6%; m.p. 179.5–182.6 °C; ^1^H NMR (600 MHz, CDCl_3_, ppm) *δ* 10.70 (s, 1H, CONH), 9.22 (s, 1H, CONH), 8.95 (s, 1H, pyrimidine-H), 7.66 (d, *J* = 8.70 Hz, 2H, Ph-H), 7.56–7.54 (m, 3H, Ph-H), 7.08–7.05 (m, 3H, Ph-H), 6.43 (t, *J* = 54.80 Hz, 1H, pyrimidine-CHF_2_); ^13^C NMR (150 MHz, CDCl_3_, ppm) *δ* 175.37, 162.14, 161.20 (d, *J* = 6.30 Hz), 159.16 (d, *J* = 6.60 Hz), 159.12, 158.07, 150.44, 139.34, 136.69, 133.95 (t, *J* = 12.30 Hz), 121.75, 121.51, 112.69 (d, *J* = 25.65 Hz), 112.66 (d, *J* = 25.65 Hz), 112.23; ^19^F NMR (565 MHz, DMSO-*d*_6_, ppm) δ −113.45, −120.68; HRMS (ESI) *m/z* calculated for C_19_H_12_F_4_N_4_O_2_S [M + Na]^+^: 459.05075, found: 495.05093.
*N*-(4-(((6-ethyl-5-fluoropyrimidin-4-yl)thio)phenyl)carbamoyl)-2,6-difluorobenzamide (**4q**). Yellow solid; yield 54.3%; m.p. 171.2–173.7 °C; ^1^H NMR (600 MHz, CDCl_3_, ppm) *δ* 10.63 (s, 1H, CONH), 9.51 (s, 1H, CONH), 8.55 (d, *J* = 2.20 Hz, 1H, pyrimidine-H), 7.59 (d, *J* = 10.20 Hz, 2H, Ph-H), 7.54–7.45 (m, 3H, Ph-H), 7.05 (t, *J* = 8.50 Hz, 2H, Ph-H), 2.90–2.76 (m, 2H, pyrimidine-CH_2_), 1.32 (t, *J* = 7.60 Hz, 3H, CH_3_); ^13^C NMR (150 MHz, CDCl_3_, ppm) *δ* 162.21, 161.13 (d, *J* = 6.75 Hz), 159.10 (d, *J* = 7.50 Hz), 156.96 (d, *J* = 18.75 Hz), 155.94 (d, *J* = 15.90 Hz), 153.45 (t, *J* = 4.16 Hz), 153.42, 153.37, 151.33, 150.69, 138.57, 136.93, 136.73, 133.78, 133.70, 133.62, 121.25, 120.93, 120.59, 112.58 (d, *J* = 25.20 Hz), 112.55 (d, *J* = 25.20 Hz), 112.33 (t, *J* = 21.60 Hz), 24.03, 11.78; ^19^F NMR (565 MHz, DMSO-*d*_6_, ppm) δ −113.34, −135.67; HRMS (ESI) *m/z* calculated for C_20_H_15_F_3_N_4_O_2_S [M + Na]^+^: 455.07559, found: 459.07600.
2,6-Difluoro-*N*-((4-(((2-(methylthio)pyrimidin-4-yl)thio)phenyl)carbamoyl)benzamide (**4r**). Yellow solid; yield 49.9%; m.p. 170.9–173.5 °C; ^1^H NMR (600 MHz, CDCl_3_, ppm) *δ* 10.64 (s, 1H, CONH), 9.04 (s, 1H, CONH), 8.13 (d, *J* = 5.40 Hz, 1H, pyrimidine-H), 7.68–7.58 (m, 2H, Ph-H), 7.54 (d, *J* = 8.40 Hz, 3H, Ph-H), 7.07 (t, *J* = 8.70 Hz, 2H, Ph-H), 6.47 (d, *J* = 5.50 Hz, 1H, pyrimidine-H), 2.42 (s, 3H, pyrimidine-SCH_3_); ^13^C NMR (150 MHz, CDCl_3_, ppm) *δ* 172.73, 172.19, 162.38, 161.11 (d, *J* = 6.75 Hz), 159.08 (d, *J* = 6.75 Hz), 155.62, 150.96, 138.78, 136.81, 133.71 (t, *J* = 12.60 Hz), 122.84, 121.11, 112.55 (d, *J* = 25.50 Hz), 112.52 (d, *J* = 25.35 Hz), 112.27 (t, *J* = 21.90 Hz), 112.17, 14.05; ^19^F NMR (565 MHz, DMSO-*d*_6_, ppm) δ −114.80; HRMS (ESI) *m/z* calculated for C_19_H_14_F_2_N_4_O_2_S_2_ [M + Na]^+^: 455.04181, found: 455.04184.
*N*-(4-(((2-chloro-5-(methylthio)pyrimidin-4-yl)thio)phenyl)carbamoyl)-2,6-difluorobenzamide (**4s**). Yellow solid; yield 52.7%; m.p. 170.9–173.5 °C; ^1^H NMR (600 MHz, CDCl_3_, ppm) *δ* 10.60 (s, 1H, CONH), 8.44 (s, 1H, CONH), 8.20 (s, 1H, pyrimidine-H), 7.67 (d, *J* = 8.10 Hz, 2H, Ph-H), 7.51 (d, *J* = 8.60 Hz, 3H, Ph-H), 7.07 (t, *J* = 8.90 Hz, 2H, Ph-H), 2.56 (s, 3H, pyrimidine-SCH_3_), ^13^C NMR (150 MHz, CDCl_3_, ppm) *δ* 170.34, 162.58, 160.01 (d, *J* = 7.05 Hz), 158.35 (d, *J* = 7.05 Hz), 156.53, 154.99, 150.44, 139.69, 136.77, 129.58, 121.20, 120.91, 112.70 (d, *J* = 20.85 Hz), 112.67 (d, *J* = 20.40 Hz); ^19^F NMR (565 MHz, DMSO-*d*_6_, ppm) δ −113.38; HRMS (ESI) *m/z* calculated for C_19_H_13_ClF_2_N_4_O_2_S_2_ [M + Na]^+^: 489.00284, found: 489.00287.
*N*-(4-(((2-chloro-5-methoxypyrimidin-4-yl)thio)phenyl)carbamoyl)-2,6-difluorobenzamide (**4t**). White solid; yield 54.0%; m. p. 199.1–203.4 °C; ^1^H NMR (600 MHz, CDCl_3_, ppm) *δ* 10.58 (s, 1H, CONH), 8.39 (s, 1H, CONH), 7.87 (s, 1H, pyrimidine-H), 7.66 (d, *J* = 8.30 Hz, 2H, Ph-H), 7.52 (d, *J* = 8.50 Hz, 3H, Ph-H), 7.07 (t, *J* = 9.00 Hz, 2H, Ph-H), 4.01 (s, 3H, pyrimidine-OCH_3_); ^13^C NMR (150 MHz, CDCl_3_, ppm) *δ* 162.58, 162.03, 160.00 (d, *J* = 6.90 Hz), 158.34 (d, *J* = 6.75 Hz), 150.57, 150.44, 149.17, 139.55, 139.01, 136.89, 121.19, 120.51, 112.71 (d, *J* = 20.70 Hz), 112.68 (d, *J* = 20.40 Hz), 57.65; ^19^F NMR (565 MHz, DMSO-*d*_6_, ppm) δ −113.41; HRMS (ESI) *m/z* calculated for C_19_H_13_ClF_2_N_4_O_3_S [M + Na]^+^: 473.02567, found: 473.02572.
*N*-((4-(((2-chloro-6-methylpyrimidin-4-yl)thio)phenyl)carbamoyl)-2,6-difluorobenzamide (**4u**). White solid; yield 58.1%; m.p. 173.9–175.8 °C; ^1^H NMR (600 MHz, CDCl_3_, ppm) *δ* 10.69 (s, 1H, CONH), 9.09 (s, 1H, CONH), 7.66 (d, *J* = 8.20 Hz, 2H, Ph-H), 7.55 (d, *J* = 8.00 Hz, 3H, Ph-H), 7.07 (t, *J* = 8.80 Hz, 2H, Ph-H), 6.46 (s, 1H, pyrimidine-H), 2.35 (s, 3H, pyrimidine-CH_3_); ^13^C NMR (150 MHz, CDCl_3_, ppm) *δ* 176.06, 169.06, 162.15, 161.19 (d, *J* = 7.05 Hz), 160.16, 159.15 (d, *J* = 6.45 Hz), 150.46, 139.25, 136.76, 134.01 (t, *J* = 12.90 Hz), 122.15, 121.51, 114.28, 112.71 (d, *J* = 25.80 Hz), 112.68 (d, *J* = 27.00 Hz), 111.94, 23.98; ^19^F NMR (565 MHz, DMSO-*d*_6_, ppm) δ −113.40; HRMS (ESI) *m/z* calculated for C_19_H_13_ClF_2_N_4_O_2_S [M + Na]^+^: 457.03064, found: 457.03080.
*N*-(4-(((2-chloropyrimidin-4-yl)thio) phenyl)carbamoyl)-2,6-difluorobenzamide (**4v**). White solid; yield 54.3%; m.p. 199.6–202.9 °C; ^1^H NMR (600 MHz, CDCl_3_, ppm) *δ* 10.66 (s, 1H, CONH), 8.64 (s, 1H, CONH), 8.20 (d, *J* = 5.40 Hz, 1H, pyrimidine-H), 7.74–7.66 (m, 2H, Ph-H), 7.61–7.52 (m, 3H, Ph-H), 7.08 (t, *J* = 8.70 Hz, 2H, Ph-H), 6.65 (d, *J* = 5.50 Hz, 1H, pyrimidine-H); ^13^C NMR (150 MHz, CDCl_3_, ppm) *δ* 176.82, 161.97, 161.23 (d, *J* = 5.40 Hz), 159.17 (d, *J* = 4.58 Hz), 160.66, 157.74, 150.02, 139.38, 136.75, 134.18 (t, *J* = 11.25 Hz), 121.93, 121.60, 115.24, 112.78 (d, *J* = 25.65 Hz), 112.74 (d, *J* = 25.20 Hz); ^19^F NMR (565 MHz, DMSO-*d*_6_, ppm) δ −113.40; HRMS (ESI) *m/z* calculated for C_18_H_11_ClF_2_N_4_O_2_S [M + Na]^+^: 443.01495, found: 443.01515.
*N*-(4-(((5-bromo-2-chloropyrimidin-4-yl)thio)phenyl)carbamoyl)-2,6-difluorobenzamide (**4w**). Yellow solid; yield 54.3%; m.p. 248.7–252.1 °C; ^1^H NMR (600 MHz, CDCl_3_, ppm) *δ* 10.61 (s, 1H, CONH), 8.36 (s, 1H, CONH), 8.32 (s, 1H, pyrimidine-H), 7.69 (d, *J* = 8.40 Hz, 2H, Ph-H), 7.54–7.49 (m, 2H, Ph-H), 7.07 (t, *J* = 8.70 Hz, 3H, Ph-H); ^13^C NMR (150 MHz, CDCl_3_, ppm) *δ* 172.11, 162.57, 160.01 (d, *J* = 6.90 Hz), 159.30, 158.38, 158.33 (d, *J* = 7.05 Hz), 150.44, 139.91, 136.78, 121.24, 120.74, 116.16, 112.70 (d, *J* = 20.40 Hz), 112.67 (d, *J* = 20.40 Hz); ^19^F NMR (565 MHz, DMSO-*d*_6_, ppm) δ −113.37; HRMS (ESI) *m/z* calculated for C_18_H_10_BrClF_2_N_4_O_2_S [M + Na]^+^: 520.92517, found: 520.92566.

### 3.3. Biological Activity Assay

#### 3.3.1. In Vitro Antifungal Activity Test

The in vitro antifungal activities of the target compounds **4a**–**4w** against *Botrytis cinerea* in cucumber, *Botrytis cinerea* in tobacco, *Botrytis cinerea* in blueberry, *Phomopsis* sp., and *Rhizoctonia solani* were tested according to the reported method [29]. Each of the target compounds (5 mg) was dissolved in 1 mL of DMSO, followed by addition of 9 mL of an aqueous solution containing Tween-20 and 90 mL of potato dextrose agar (PDA) medium. Subsequently, the resulting PDA medium mixture was poured into 6 Petri dishes to prepare PDA plates. Afterward, mycelial discs with a diameter of 0.4 cm were aseptically placed at the center of each PDA plate and incubated at 28 °C for a period of 3−4 days until the mycelia reached a diameter range between 6 and 7 cm. DMSO was utilized as a negative control, while hymexazol served as a positive control. The inhibition rate I (%) is calculated using the following formula, where C (cm) and T (cm) represent the fungal diameters of untreated and treated PDA plates, respectively.

Inhibition rate I (%) = (C − T)/(C − 0.4) × 100(1)

#### 3.3.2. In Vitro Antibacterial Activity Test

The in vitro antibacterial activities of the target compounds **4a**–**4w** against *Xanthomonas oryzae* pv. *oryzicola* and *Xanthomonas citri* subsp. *citri* were tested according to the reported method [30]. Each target compound (7.5 mg) was dissolved in 150 μL of DMSO and then a mixture solution of 80 and 40 μL was poured into 15 mL centrifuge tubes containing 4 mL of a 0.1% Twain aqueous solution. Next, the resulting mixture solutions (1 mL) were added to test tubes containing 4 mL nutrient broth (NB) medium to prepare test solutions with concentrations of 200 and 100 μg/mL, respectively. Then, NB medium (40 μL) containing *Xanthomonas oryzae* pv. *oryzicola* or *Xanthomonas citri* subsp. *citri* was added to each test tube mentioned above. The inoculated test tubes were incubated at a temperature of 30 °C and a speed of 180 rpm for up to 48 h until the OD_595_ values reached between 0.6–0.8 during the logarithmic growth phase as determined by monitoring on a Multiskan Sky1530 spectrophotometer (Thermo Scientific, Wilmington, Poland). DMSO served as the negative control while thiodiazole copper served as a positive control. Inhibition rate I (%) was calculated using the following formula, where C represents the corrected turbidity value of the untreated NB mediums, and T is the corrected turbidity value of the treated NB mediums.

Inhibition rate I (%) = (C − T)/C × 100(2)

### 3.4. Molecular Modeling

The SDH enzyme plays a crucial role in the Krebs cycle, making it an appealing target for the development of SDHIs based on antifungal agents [31]. In order to investigate the mechanism of action and target interaction, we selected the binding modes between SDH and the highly active compound **4l** as an example, using Discovery Studio 2.5 software (Accelrys Inc., San Diego, CA, USA). The three-dimensional (3D) structure of compound **4l** was depicted using ChemDraw Ultra 20.0 software (PerkinElmer, Waltham, MA, USA). The protein SDH receptor (PDB: 2FBW) structure was obtained from the RCBs PDB database (https://www.rcsb.org/structure/2FBW, accessed on 1 August 2023). A molecular docking study was conducted to investigate the binding mode of compound **4l** with SDH utilizing the CDOCKER program of the Discovery Studio 2.5 software following the reported methodology [32].

## 4. Conclusions

In this study, a total of 23 novel benzoylurea derivatives containing a pyrimidine moiety were synthesized through condensation, acylation, and thioetherification reactions. Compounds **4j** and **4l** reveal good antifungal activity against *Rhizoctonia solani*, with EC_50_ values of 6.72 and 5.21 μg/mL, respectively, which were similar to that of hymexazol (6.11 μg/mL). Molecular docking simulation revealed that compound **4l** interacted with SER-17 and SER-39 through the hydrogen bond. This study provides a foundation for the further development of novel benzoylurea derivatives containing a pyrimidine moiety, which can be used to control plant fungal and bacterial diseases.

## Figures and Tables

**Table 1:** Antifungal activity of the target compounds against the test fungi at concentrations of 50 μg/mL.

Compounds	Inhibition Rate (%) ^a^				
	*Botrytis cinerea* in Cucumber	*Rhizoctonia solani*	*Botrytis cinerea* in Tobacco	*Phomopsis* sp.	*Botrytis cinerea* in Blueberry
**4a**	24.92 ± 2.24	55.41 ± 1.46	51.26 ± 1.60	30.35 ± 1.38	39.94 ± 4.36
**4b**	15.20 ± 1.36	52.03 ± 1.12	38.99 ± 1.17	27.48 ± 1.43	30.82 ± 1.00
**4c**	39.51 ± 4.68	51.35 ± 2.55	45.49 ± 1.60	32.27 ± 1.29	55.35 ± 1.31
**4d**	22.49 ± 2.05	46.96 ± 2.26	37.18 ± 1.25	29.39 ± 0.96	33.96 ± 1.54
**4e**	21.88 ± 3.46	53.04 ± 1.30	42.24 ± 1.25	28.12 ± 1.14	46.86 ± 2.12
**4f**	32.52 ± 1.57	54.73 ± 1.38	50.54 ± 1.85	28.12 ± 1.14	44.03 ± 2.90
**4g**	16.41 ± 1.57	57.09 ± 1.38	50.90 ± 1.17	28.43 ± 0.99	54.72 ± 1.83
**4h**	21.58 ± 2.82	56.08 ± 1.10	44.77 ± 1.03	37.38 ± 1.02	42.77 ± 4.49
**4i**	3.65 ± 1.30	54.39 ± 2.49	36.82 ± 1.26	40.89 ± 1.01	46.54 ± 1.56
**4j**	18.84 ± 1.86	70.27 ± 1.43	46.93 ± 1.46	49.84 ± 1.47	52.20 ± 3.26
**4k**	17.33 ± 1.58	47.30 ± 1.43	39.35 ± 1.22	30.35 ± 0.79	45.28 ± 1.58
**4l**	50.15 ± 1.82	89.74 ± 8.84	47.65 ± 1.21	42.17 ± 1.34	53.77 ± 3.62
**4m**	30.70 ± 1.55	47.30 ± 1.63	41.16 ± 1.19	25.24 ± 0.87	42.77 ± 2.04
**4n**	10.94 ± 1.13	46.62 ± 1.57	36.82 ± 1.46	21.73 ± 0.91	34.28 ± 1.77
**4o**	9.12 ± 0.92	47.30 ± 2.60	40.07 ± 1.54	30.99 ± 0.91	35.85 ± 1.23
**4p**	20.36 ± 3.33	49.32 ± 1.94	36.10 ± 1.75	23.64 ± 2.38	2.83 ± 1.15
**4q**	43.47 ± 3.44	57.77 ± 2.62	40.07 ± 1.39	28.75 ± 0.96	36.16 ± 1.41
**4r**	29.48 ± 2.66	55.41 ± 1.46	37.91 ± 1.20	31.63 ± 0.86	42.14 ± 1.52
**4s**	15.50 ± 1.09	47.64 ± 1.55	44.77 ± 2.26	30.99 ± 0.91	44.97 ± 1.66
**4t**	9.42 ± 1.05	40.88 ± 1.12	35.74 ± 1.18	26.20 ± 1.12	26.10 ± 2.81
**4u**	24.92 ± 2.74	50.00 ± 1.78	39.35 ± 1.09	32.91 ± 1.55	62.26 ± 2.07
**4v**	12.16 ± 1.79	48.65 ± 1.75	36.46 ± 1.18	38.02 ± 1.08	35.53 ± 1.61
**4w**	6.38 ± 1.04	51.01 ± 1.61	40.07 ± 1.39	36.42 ± 2.84	29.56 ± 4.12
Hymexazol	24.64 ± 3.09	71.98 ± 2.00	72.88 ± 2.30	47.09 ± 1.69	71.26 ± 4.43

^a^ Average of three replicates.

**Table 2:** The EC_50_ values of compounds **4j** and **4l** against *Rhizoctonia solani*.

Compounds	Toxic Regression Equation	R^2^	EC_50_ (μg/mL) ^a^
**4j**	y = 0.47x + 3.43	0.98	6.72 ± 0.95
**4l**	y = 0.58x + 3.49	0.94	5.21 ± 1.04
Hymexazol	y = 0.78x + 4.39	0.99	6.11 ± 1.24

^a^ Average of three replicates.

**Table 3:** Antibacterial activity of the target compounds against the test bacteria at concentrations of 200 and 100 μg/mL.

Compounds	Inhibition Rate (%) ^a^			
	*Xanthomonas oryzae* pv. *oryzicola*		*Xanthomonas citri* subsp. *citri*	
	200 μg/mL	100 μg/mL	200 μg/mL	100 μg/mL
**4a**	34.41 ± 2.78	19.46 ± 3.21	48.81 ± 2.68	32.59 ± 2.58
**4b**	26.36 ± 1.60	19.89 ± 1.91	29.77 ± 1.90	17.0 ± 0.30
**4c**	10.56 ± 2.70	6.69 ± 3.50	15.39 ± 2.70	7.70 ± 2.40
**4d**	35.98 ± 2.20	20.54 ± 1.03	19.90 ± 2.39	6.90 ± 2.47
**4e**	20.58 ± 1.96	19.78 ± 2.63	22.83 ± 2.70	13.32 ± 2.34
**4f**	35.10 ± 0.93	20.56 ± 3.28	31.09 ± 2.94	24.45 ± 1.55
**4g**	26.32 ± 1.75	23.22 ± 3.40	52.19 ± 2.71	31.60 ± 2.70
**4h**	43.48 ± 2.20	29.03 ± 2.45	38.93 ± 2.80	20.27 ± 2.00
**4i**	14.79 ± 1.43	6.28 ± 1.27	25.24 ± 2.41	6.98 ± 1.43
**4j**	30.17 ± 3.07	19.01 ± 2.96	31.66 ± 2.36	16.91 ± 3.75
**4k**	27.85 ± 3.26	26.85 ± 3.88	41.44 ± 2.64	16.85 ± 1.42
**4l**	35.78 ± 2.14	19.89 ± 4.16	35.25 ± 2.34	15.14 ± 2.41
**4m**	29.96 ± 2.12	26.22 ± 1.29	32.45 ± 2.38	15.55 ± 2.56
**4n**	39.85 ± 2.14	14.82 ± 2.77	32.84 ± 2.63	13.81 ± 0.26
**4o**	28.02 ± 2.58	17.00 ± 3.30	26.23 ± 2.14	14.03 ± 2.14
**4p**	18.57 ± 1.90	16.51 ± 2.54	39.43 ± 1.93	18.1 ± 2.86
**4q**	25.34 ± 1.16	19.01 ± 0.57	46.55 ± 2.95	25.08 ± 2.95
**4r**	29.44 ± 3.16	16.44 ± 2.41	44.13 ± 3.20	28.81 ± 1.19
**4s**	16.51 ± 2.03	14.82 ± 3.38	32.55 ± 3.21	11.24 ± 2.17
**4t**	33.85 ± 2.17	11.06 ± 2.29	30.62 ± 2.21	22.15 ± 2.90
**4u**	22.22 ± 1.90	17.02 ± 2.01	55.24 ± 1.03	24.77 ± 2.79
**4v**	21.94 ± 2.01	15.76 ± 3.03	66.80 ± 2.30	24.96 ± 3.02
**4w**	26.75 ± 1.70	19.85 ± 2.17	55.67 ± 1.75	38.88 ± 2.25
Thiodiazole copper	62.51 ± 1.52	46.42 ± 2.91	76.59 ± 3.10	48.01 ± 2.33

^a^ Average of three replicates.

## Data Availability

Not applicable.

## Supplementary Materials

The following supporting information can be downloaded at: https://www.mdpi.com/article/10.3390/molecules28186498/s1.

## Sample Availability

Samples of the compounds **4a**–**4w** are available from the authors.
