# Supplementary material for: Synthesis, Antifungal, and Antibacterial Activities of Novel Benzoylurea Derivatives Containing a Pyrimidine Moiety
Source: Molecules. 2023 Sep 7;28(18):6498. doi: 10.3390/molecules28186498 (PMC10535663; doi:10.3390/molecules28186498)
Supplement: Supplementary file 1 [file molecules-28-06498-s001.zip › molecules-2585733-supplementary.pdf]

Synthesis, antifungal, and antibacterial activities of novel benzoylurea derivatives containing a pyrimidine moiety

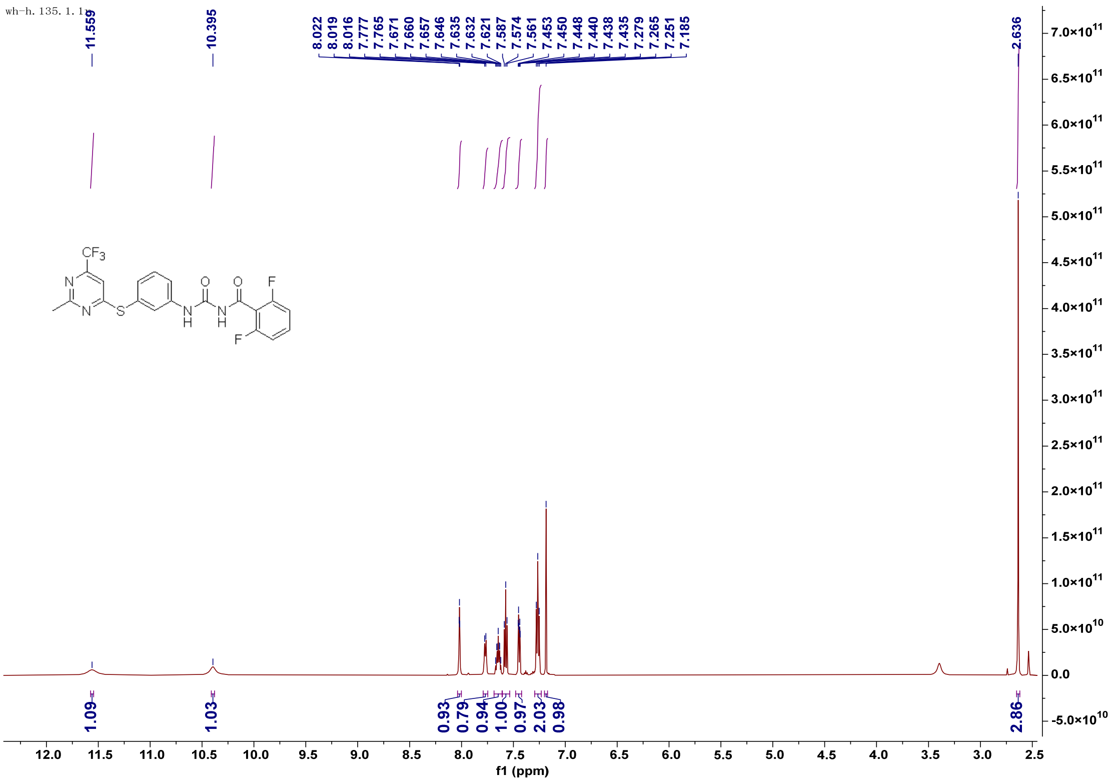

Figure S1 ^{1}H NMR spectrum of compound **4a**

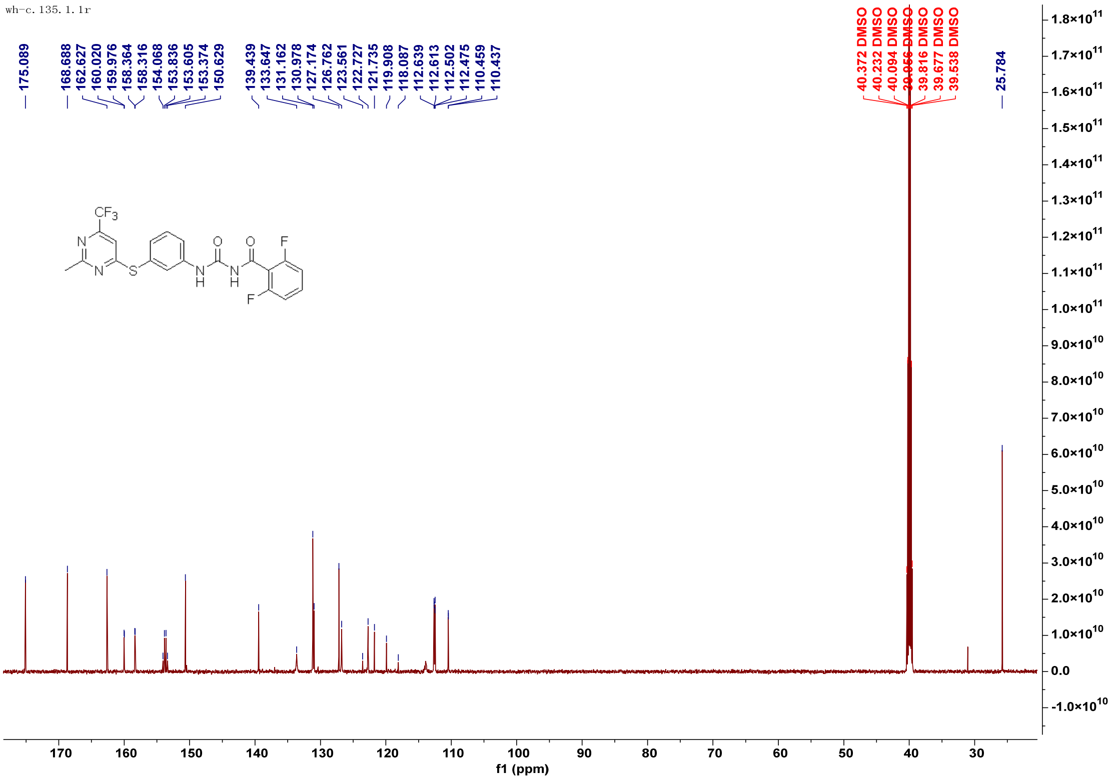

Figure S2 ^{13}C NMR spectrum of compound **4a**

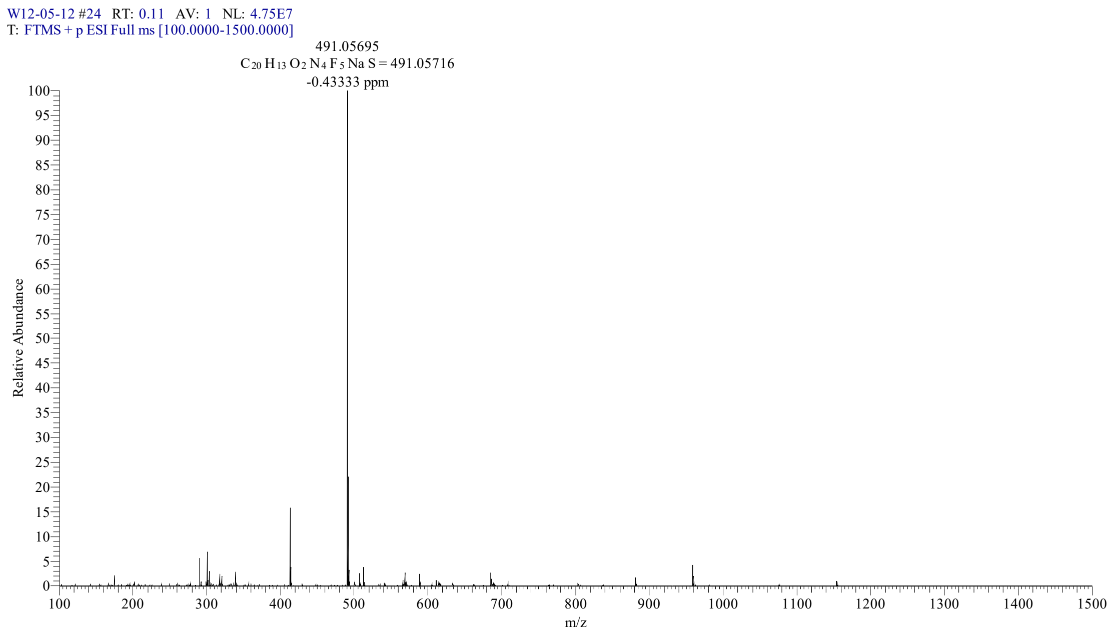

Figure S3 HRMS spectrum of compound **4a**

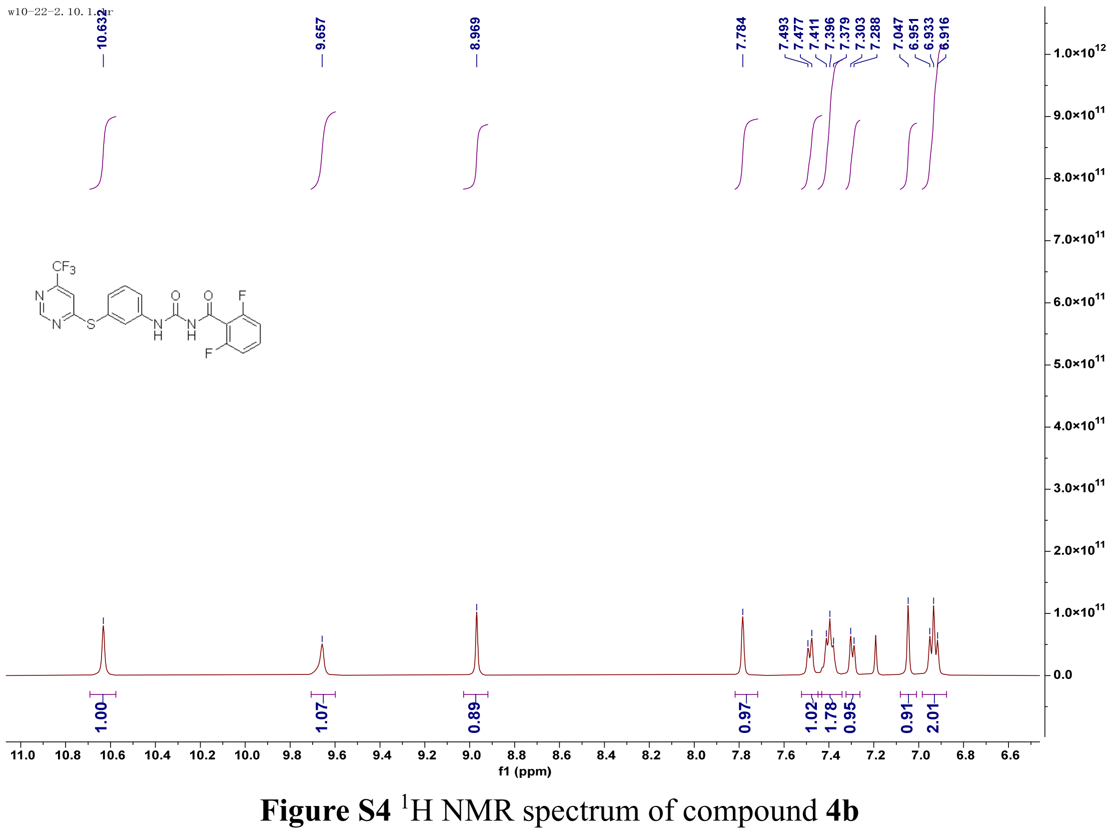

Figure S4 ^{1}H NMR spectrum of compound **4b**

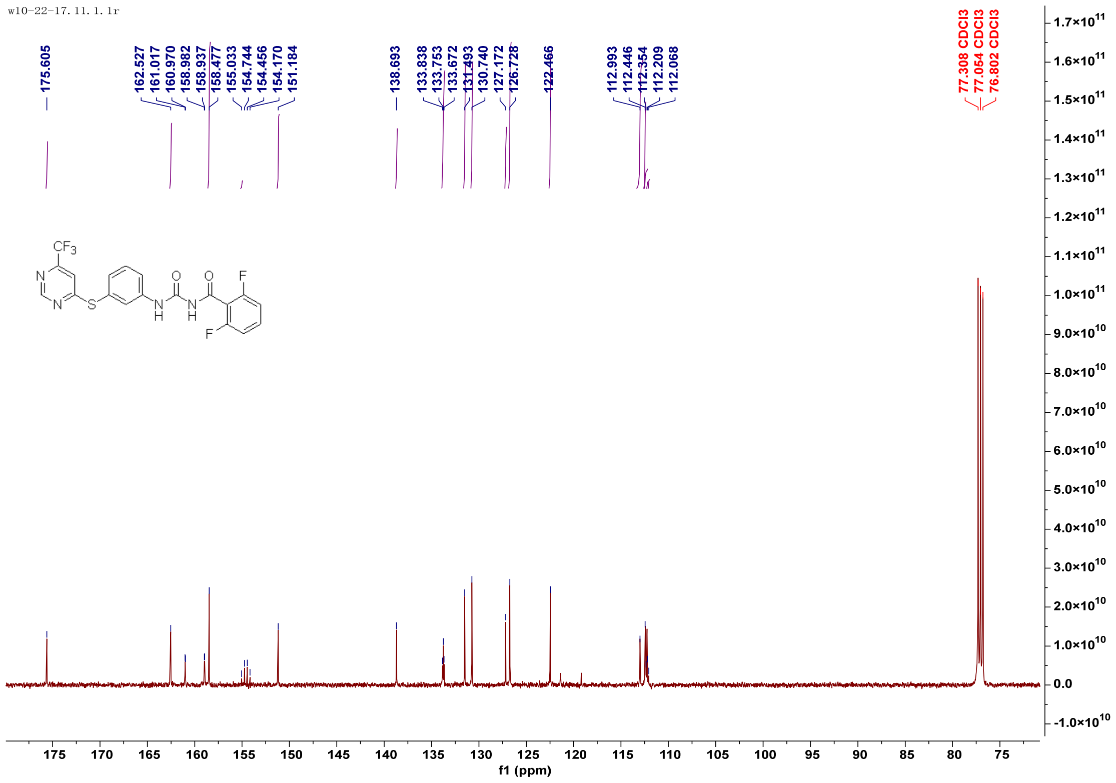

Figure S5 ^{13}C NMR spectrum of compound **4b**

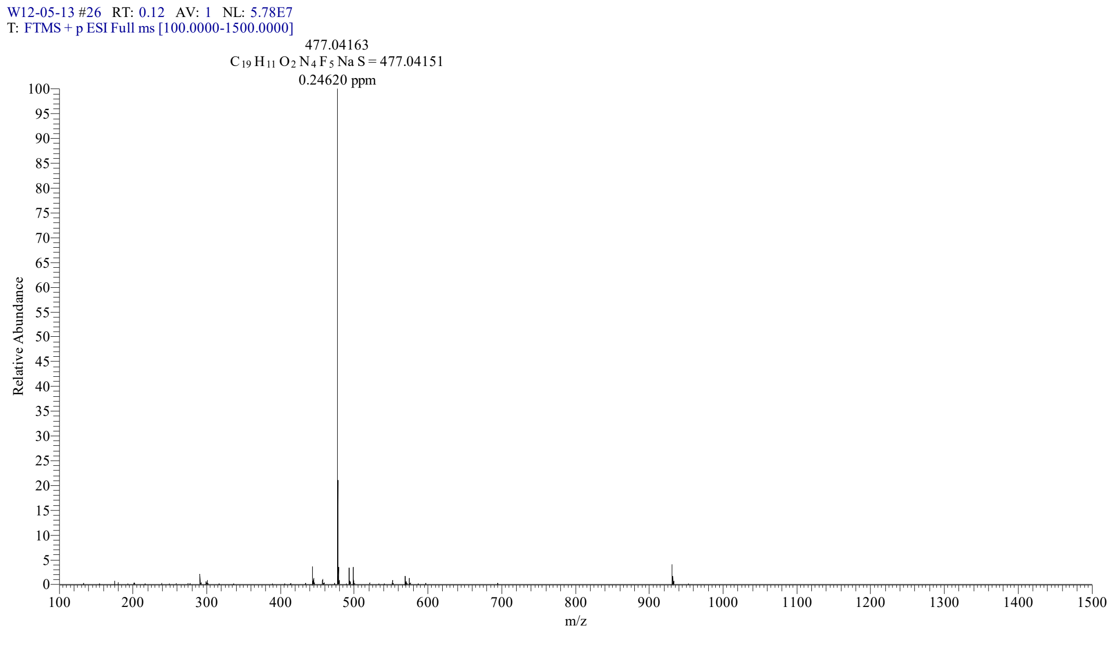

Figure S6 HRMS spectrum of compound **4b**

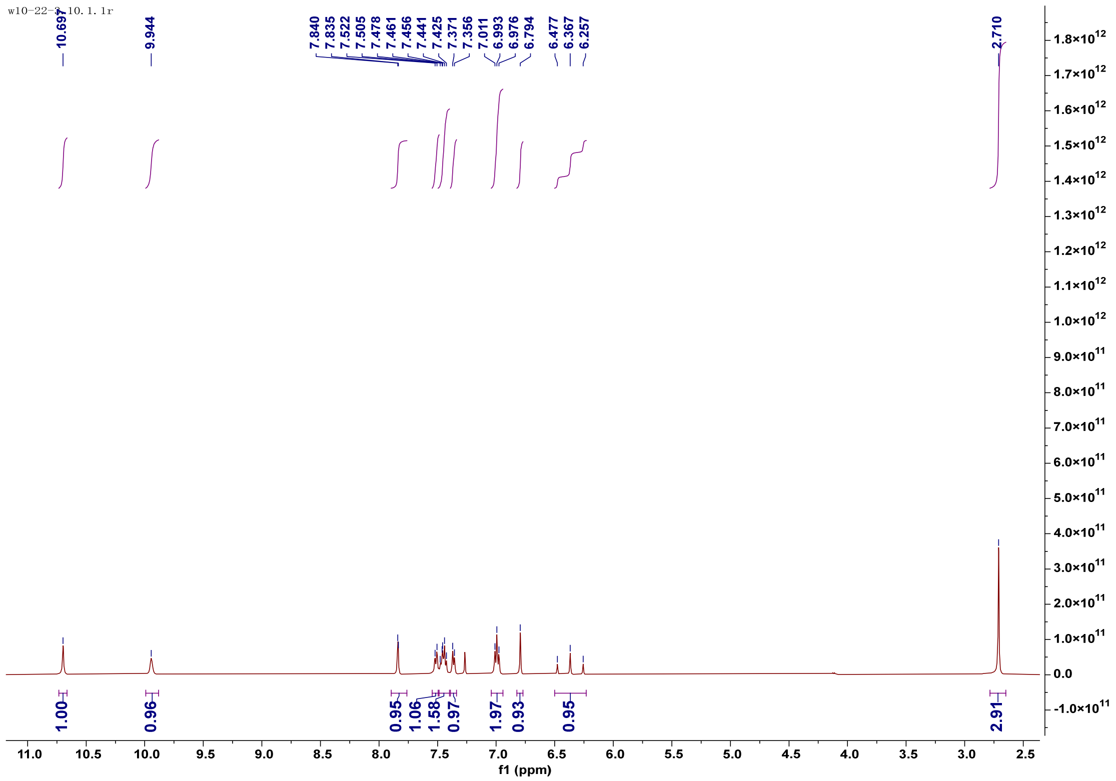

Figure S7 ^{1}H NMR spectrum of compound **4c**

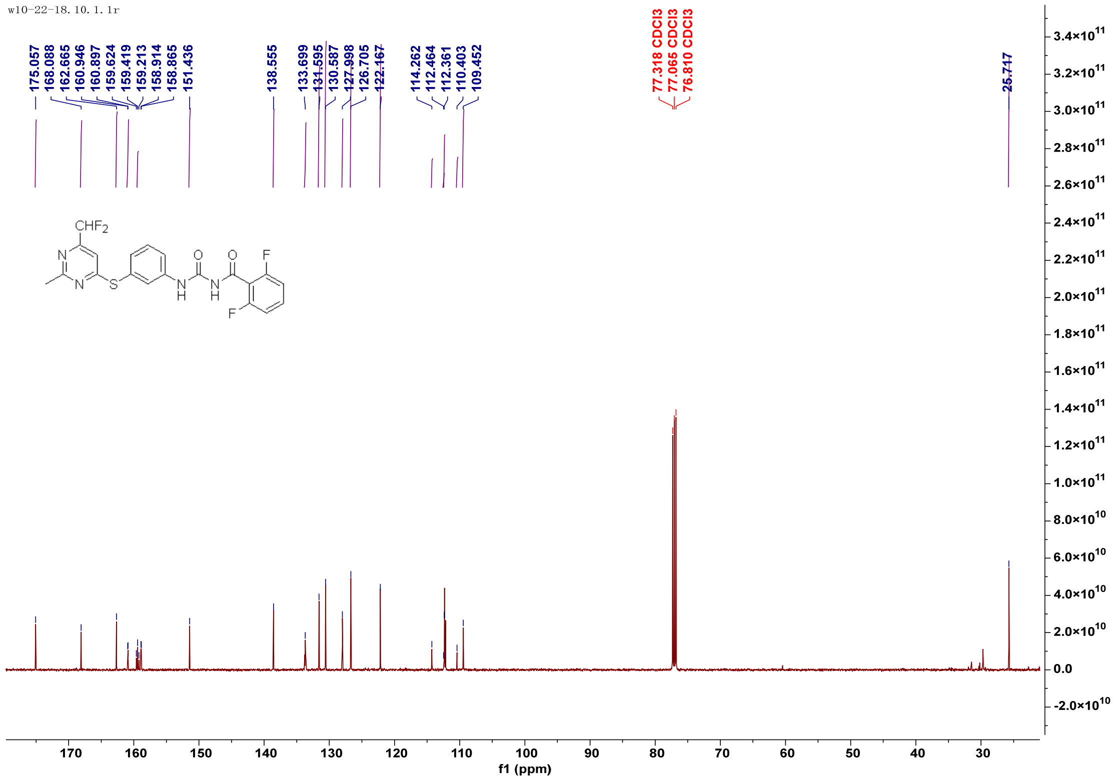

Figure S8 ^{13}C NMR spectrum of compound **4c**

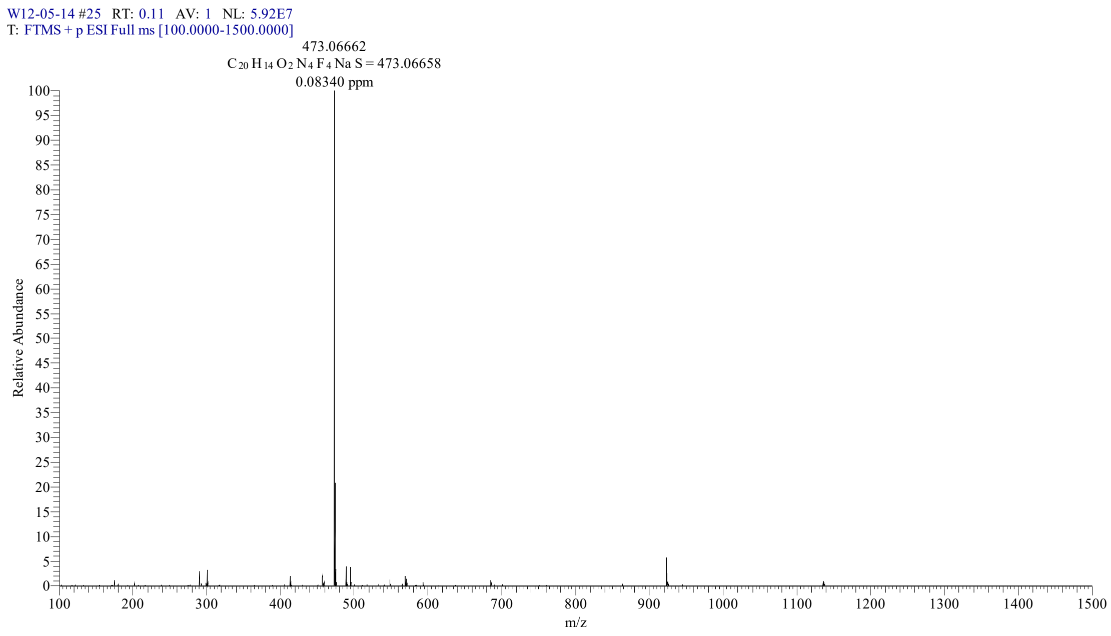

Figure S9 HRMS spectrum of compound **4c**

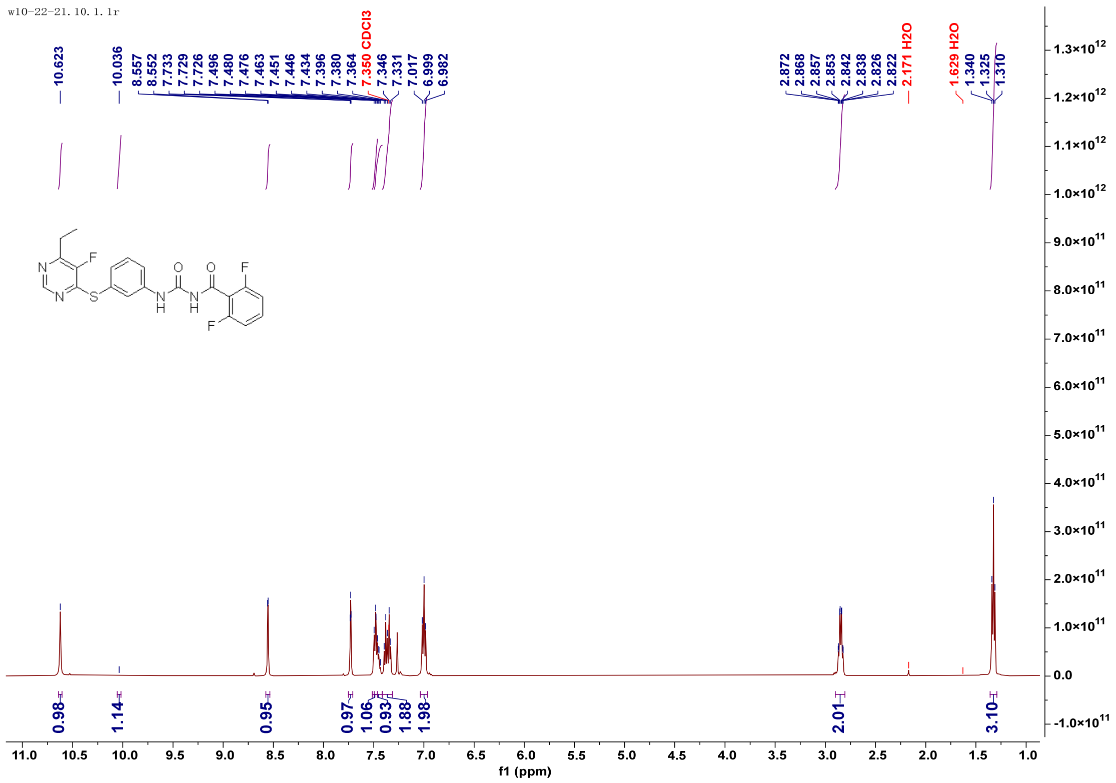

Figure S10 ^{1}H NMR spectrum of compound **4d**

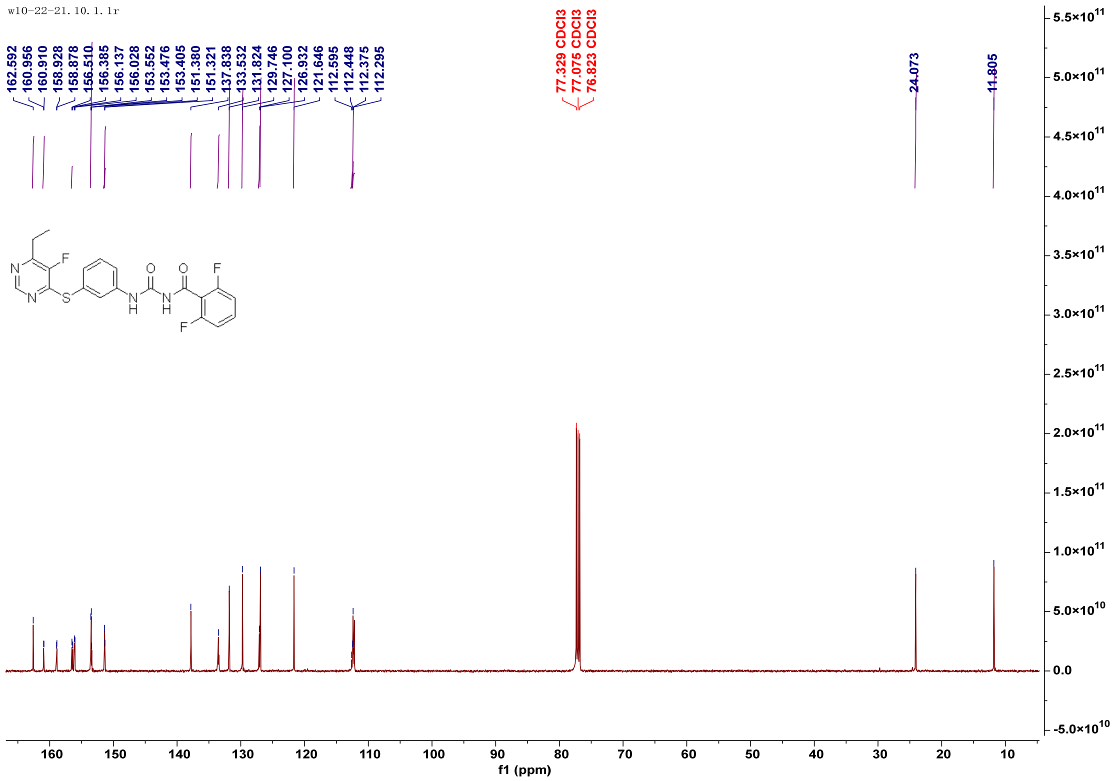

Figure S11 ^{13}C NMR spectrum of compound **4d**

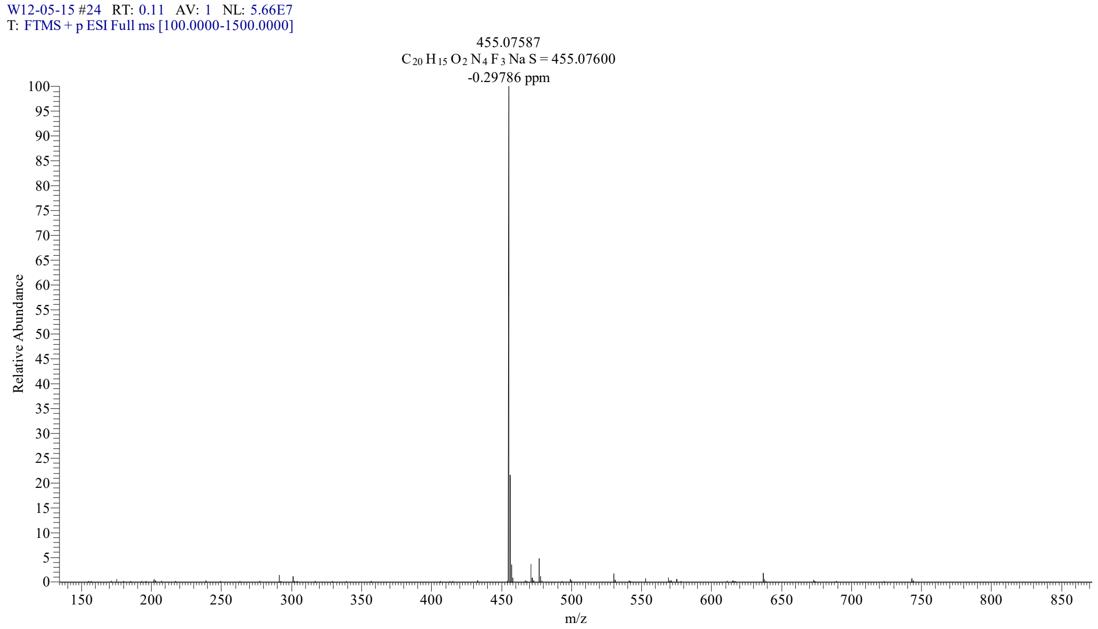

Figure S12 HRMS spectrum of compound **4d**

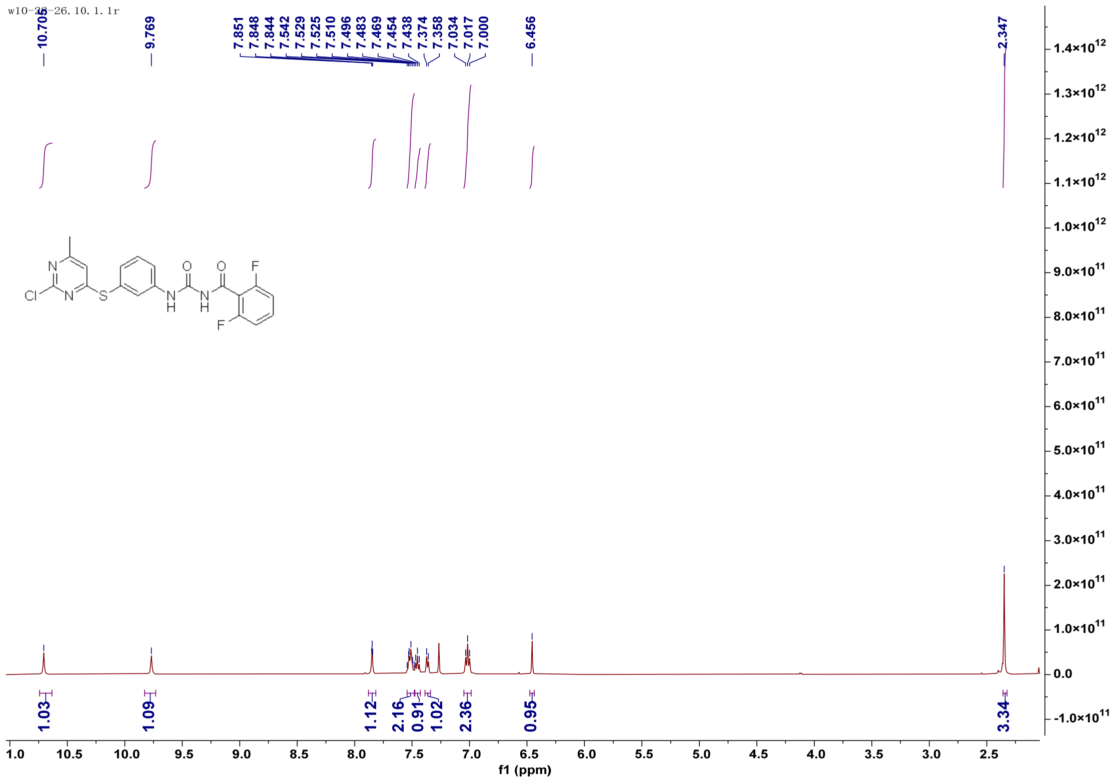

Figure S13 ^{1}H NMR spectrum of compound **4e**

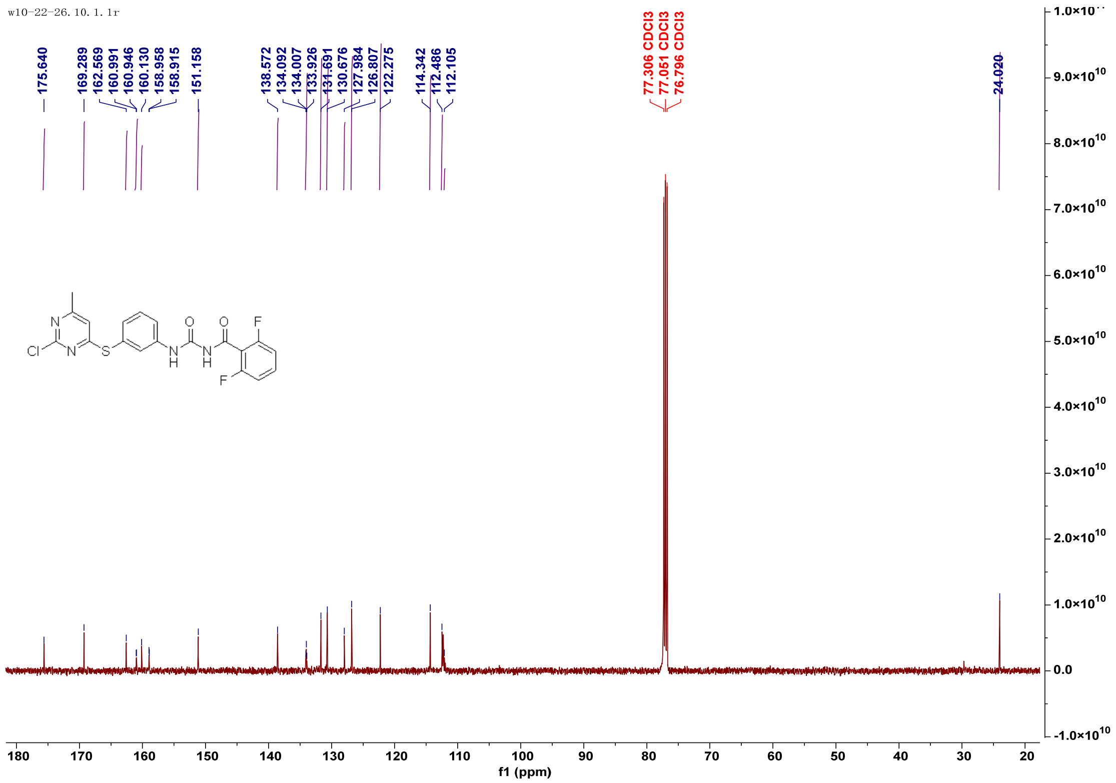

Figure S14 ^{13}C NMR spectrum of compound **4e**

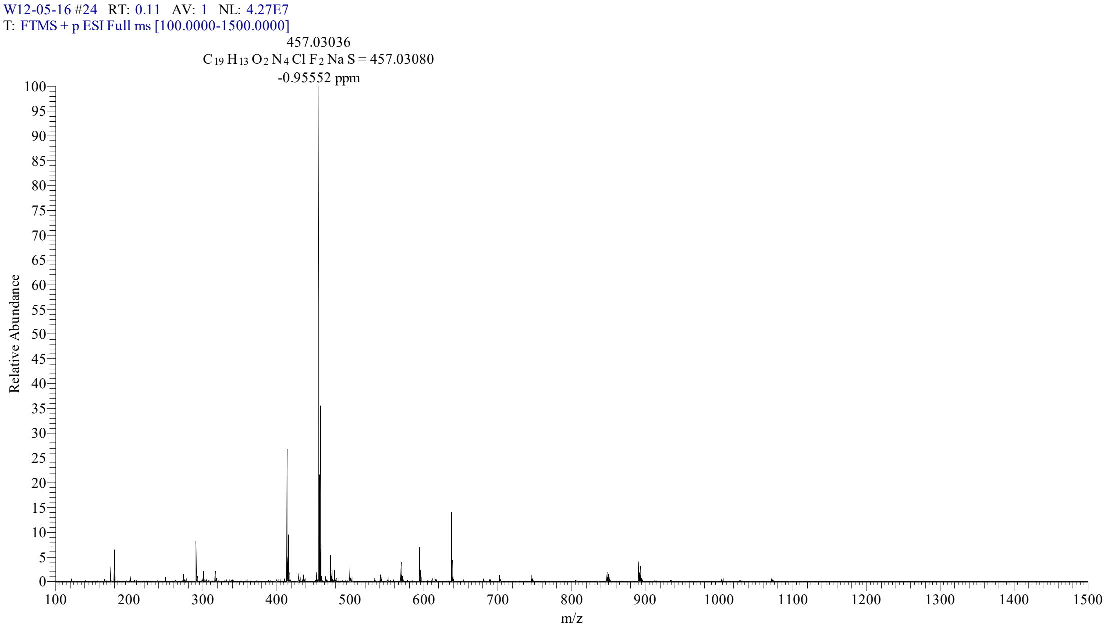

Figure S15 HRMS spectrum of compound **4e**

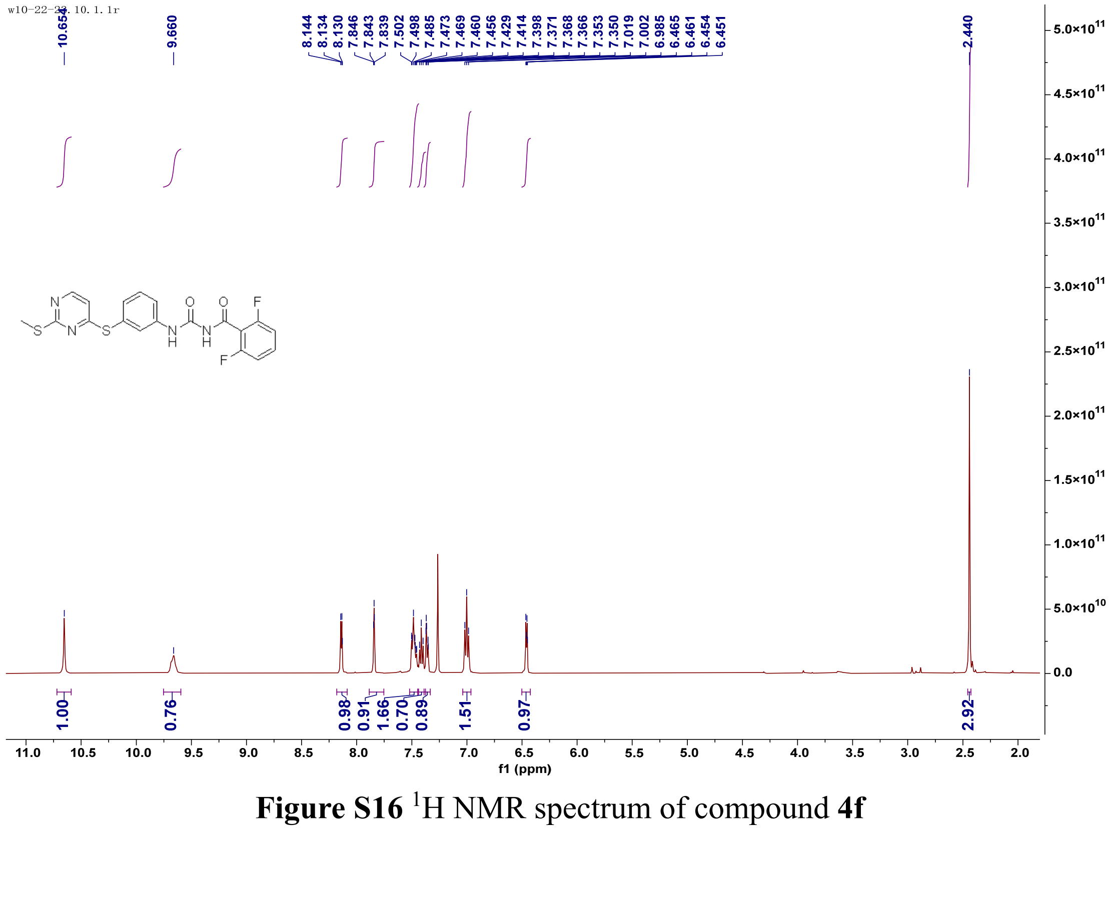

Figure S16 ^{1}H NMR spectrum of compound **4f**

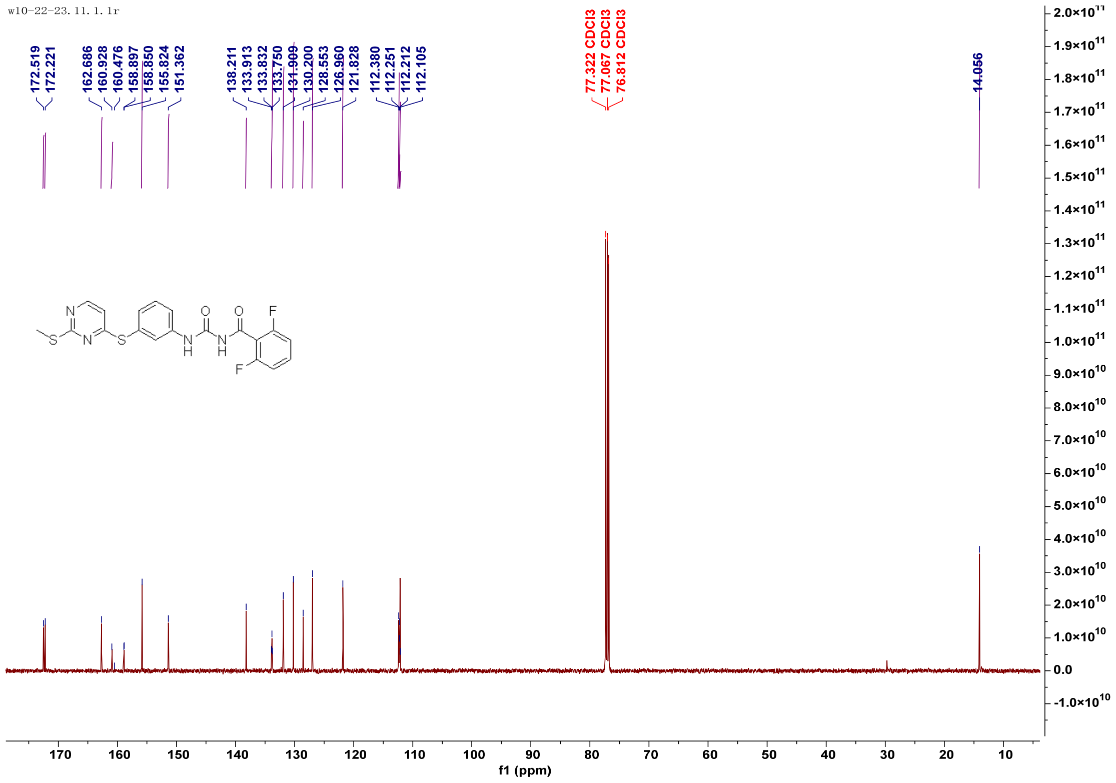

Figure S17 ^{13}C NMR spectrum of compound**4f**

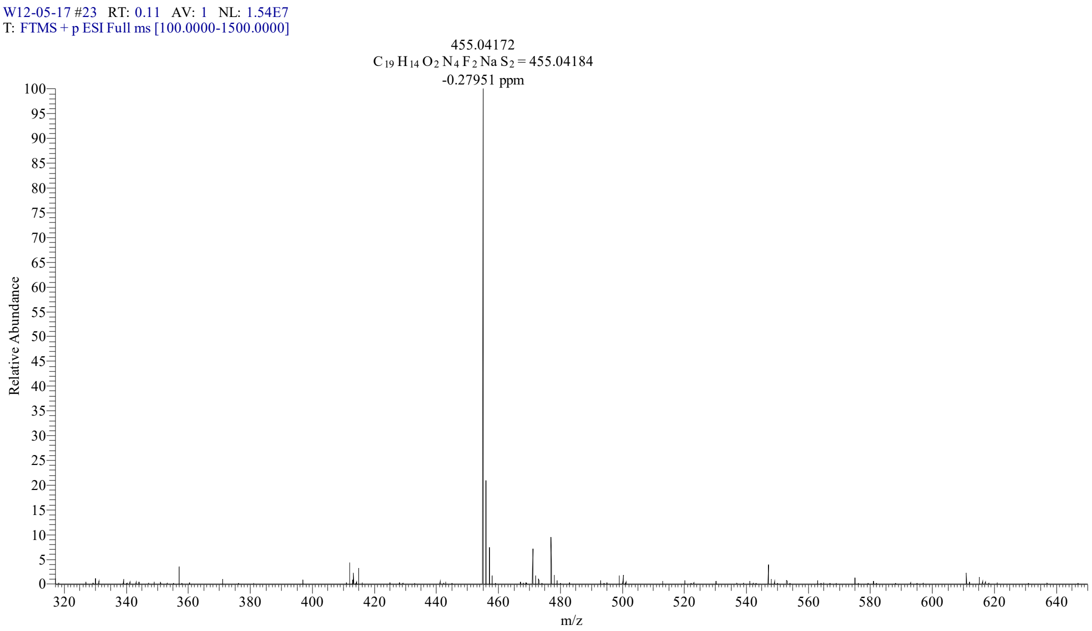

Figure S18 HRMS spectrum of compound **4f**

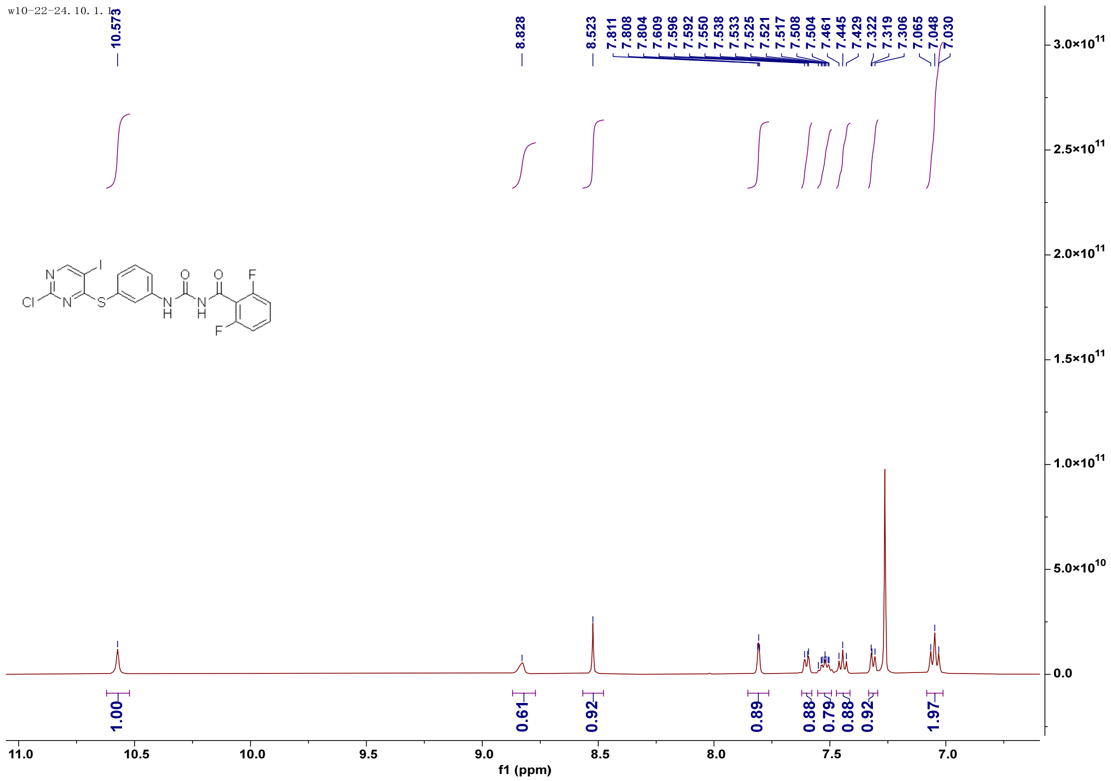

Figure S19 ^{1}H NMR spectrum of compound **4g**

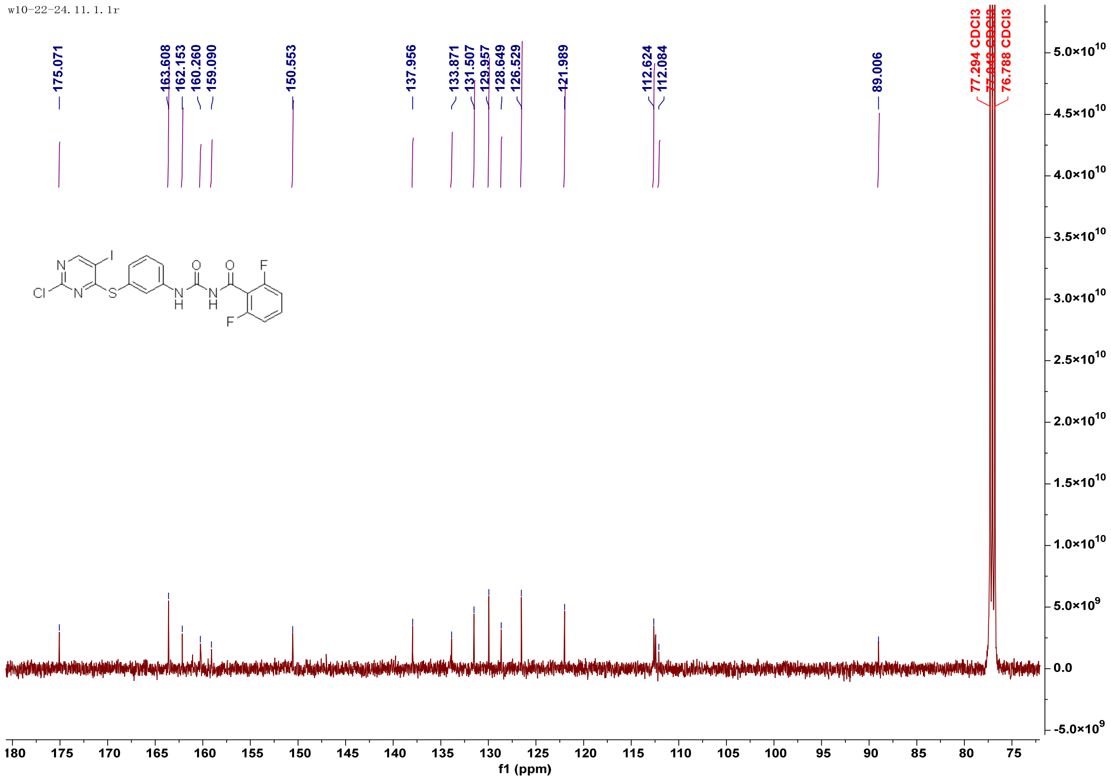

Figure S20 ^{13}C NMR spectrum of compound **4g**

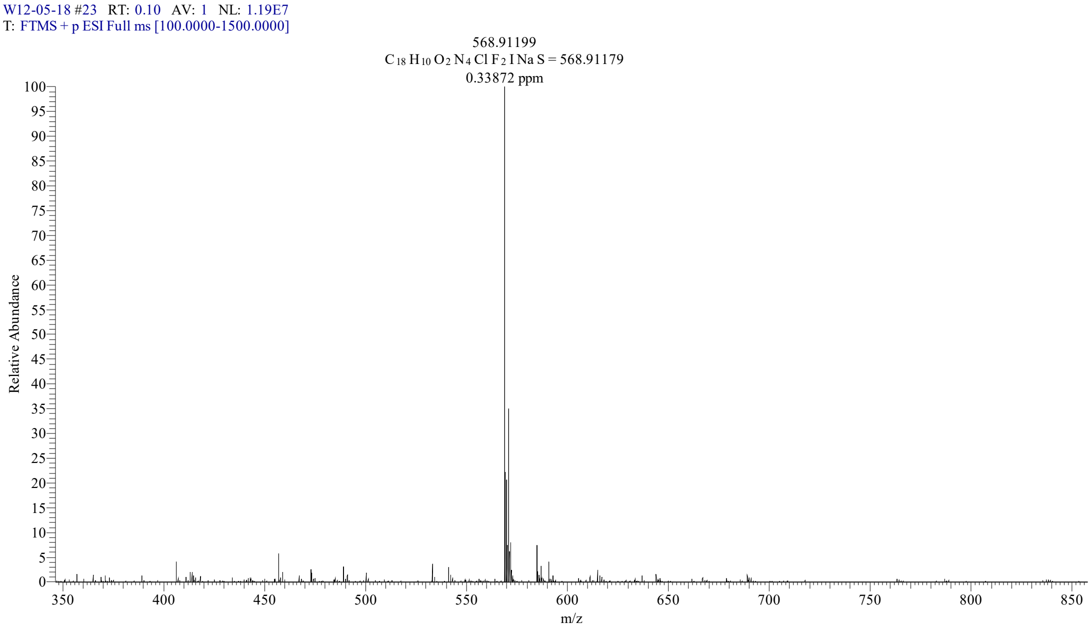

Figure S21 HRMS spectrum of compound **4g**

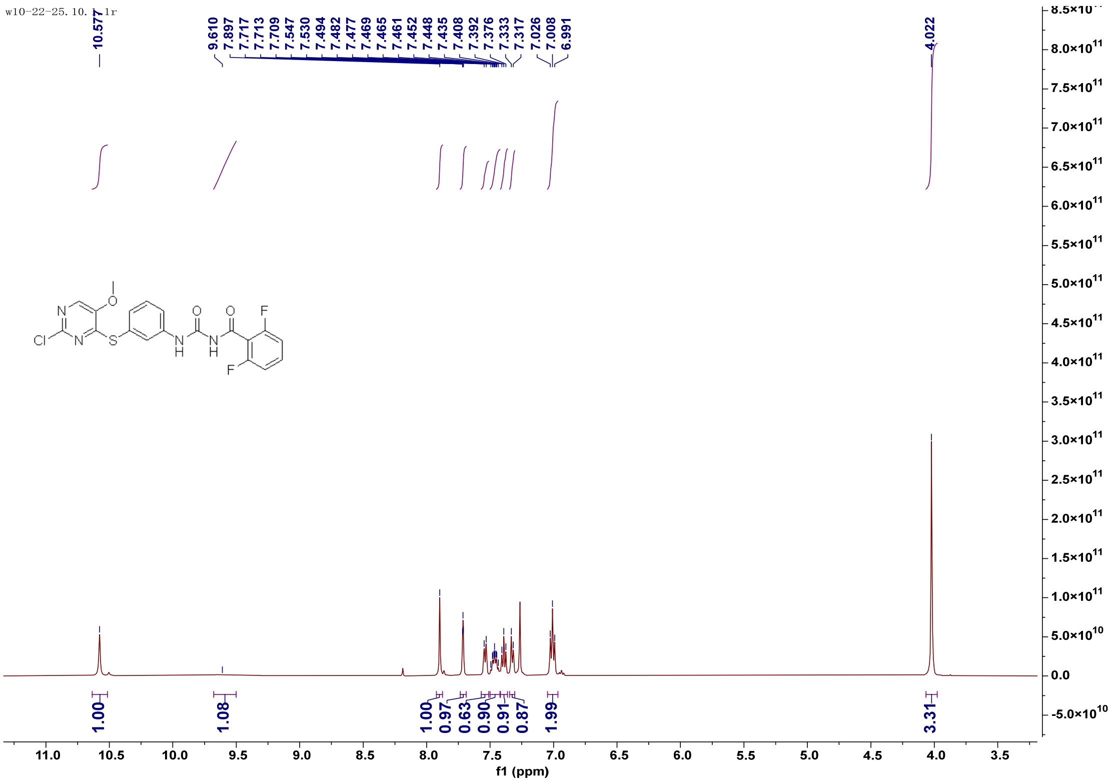

Figure S22 ^{1}H NMR spectrum of compound **4h**

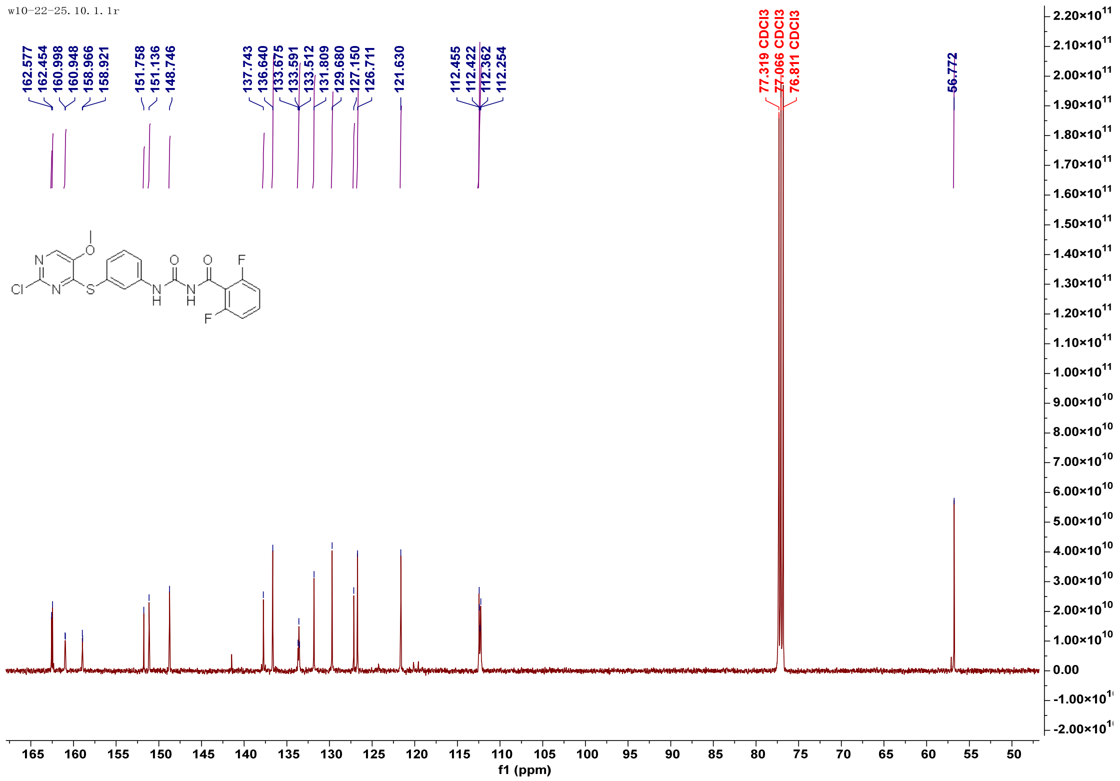

Figure S23 ^{13}C NMR spectrum of compound **4h**

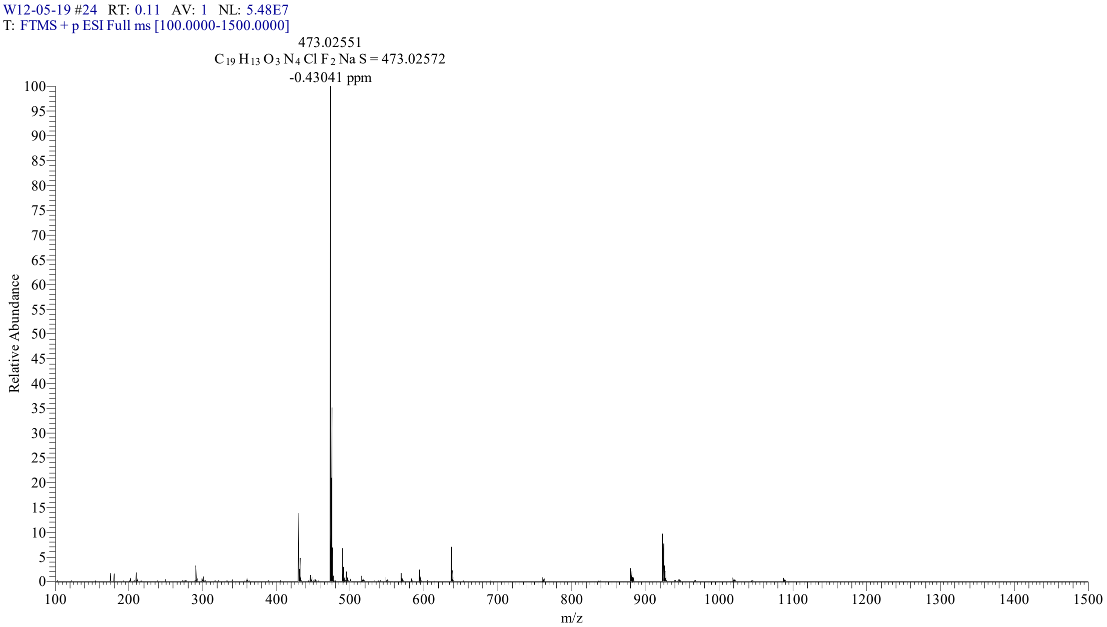

Figure S24 HRMS spectrum of compound **4h**

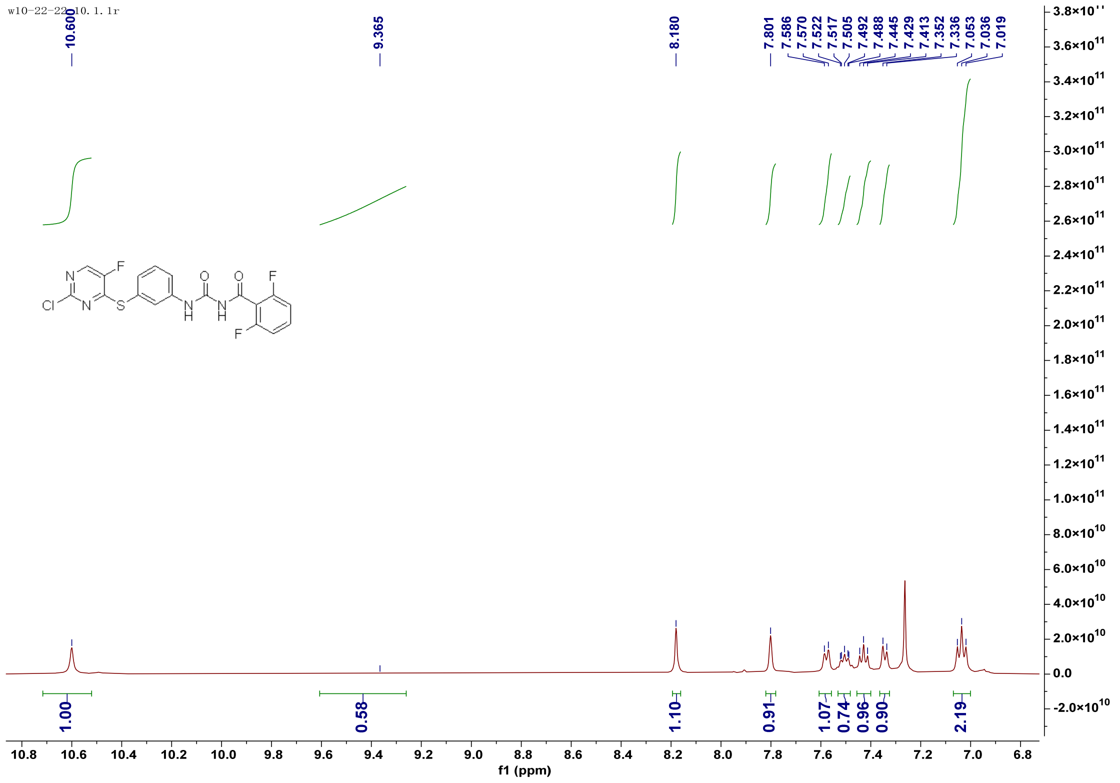

Figure S25 ^{1}H NMR spectrum of compound **4i**

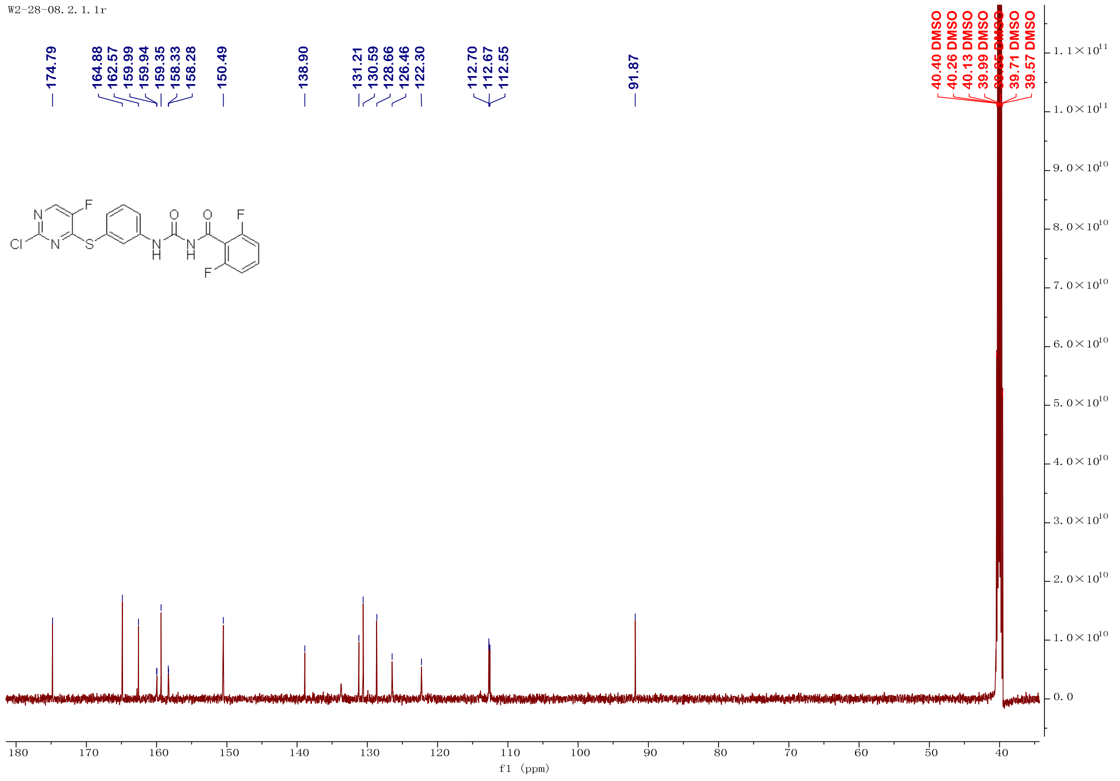

Figure S26 ^{13}C NMR spectrum of compound **4i**

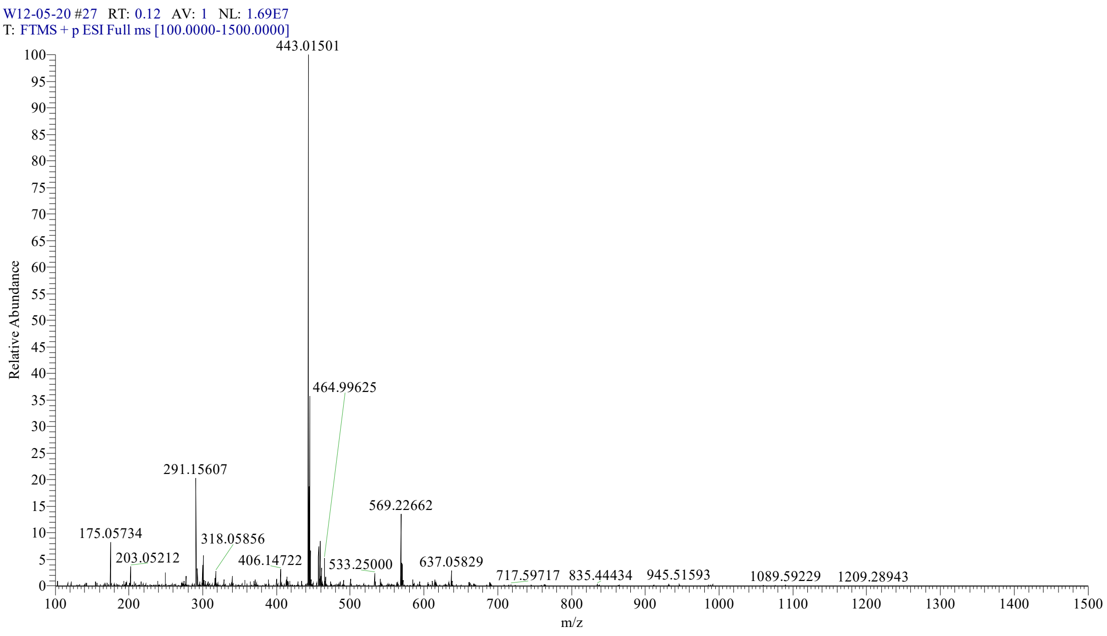

Figure S27 HRMS spectrum of compound **4i**

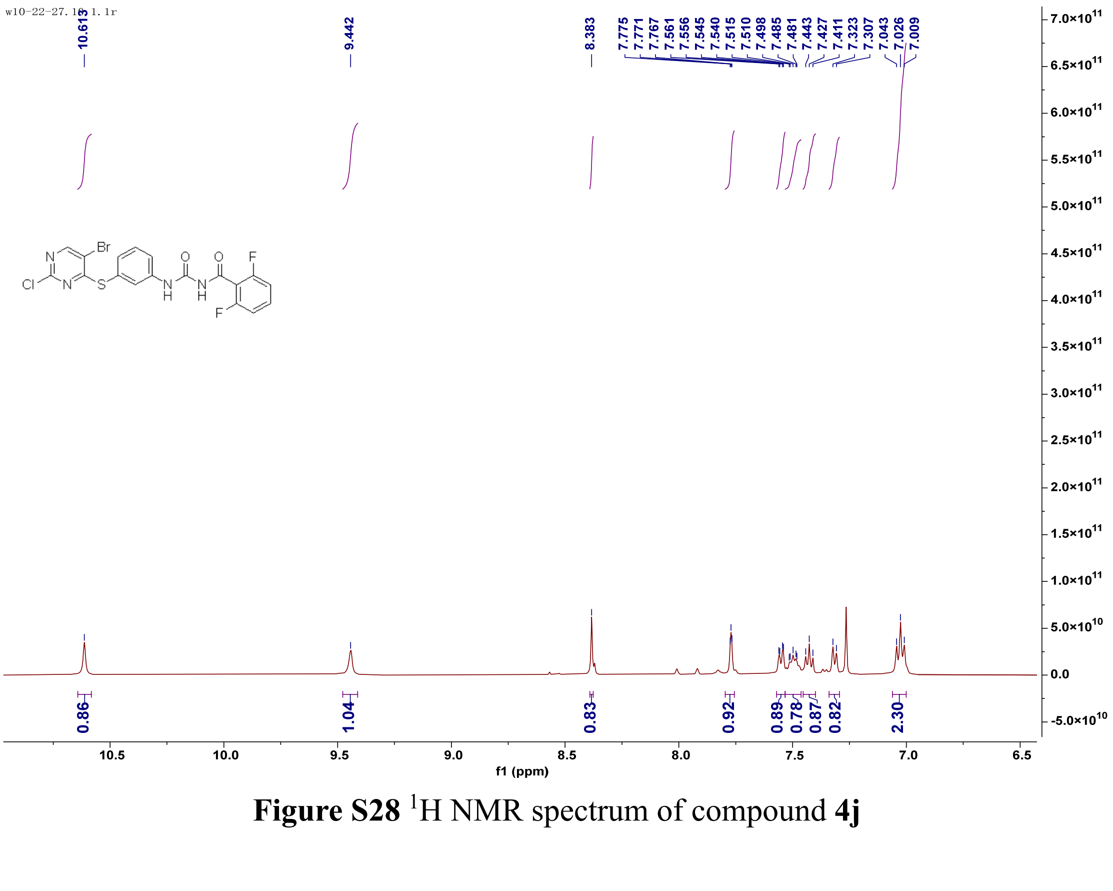

Figure S28 ^{1}H NMR spectrum of compound **4j**

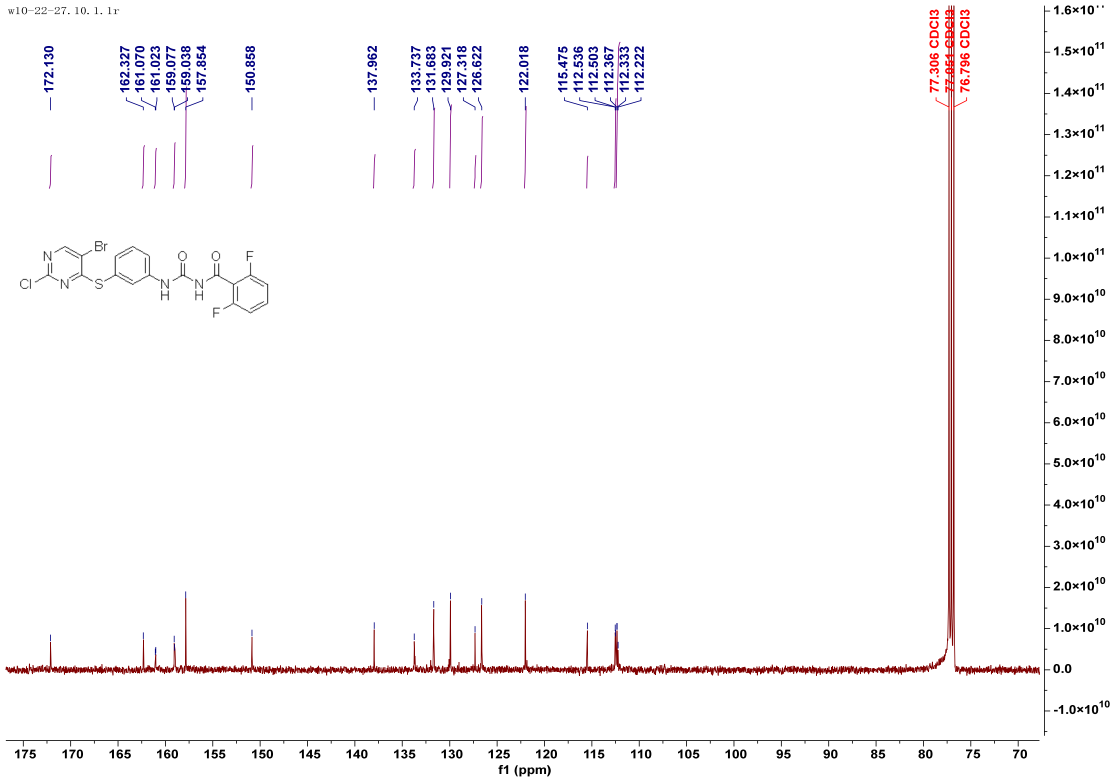

Figure S29 ^{13}C NMR spectrum of compound **4j**

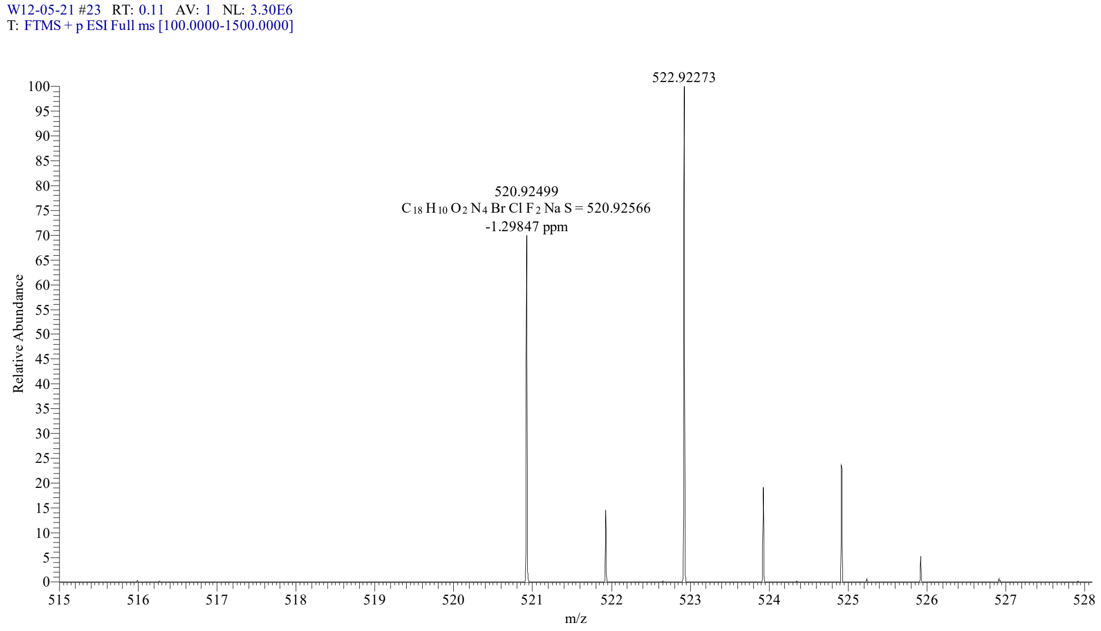

Figure S30 HRMS spectrum of compound **4j**

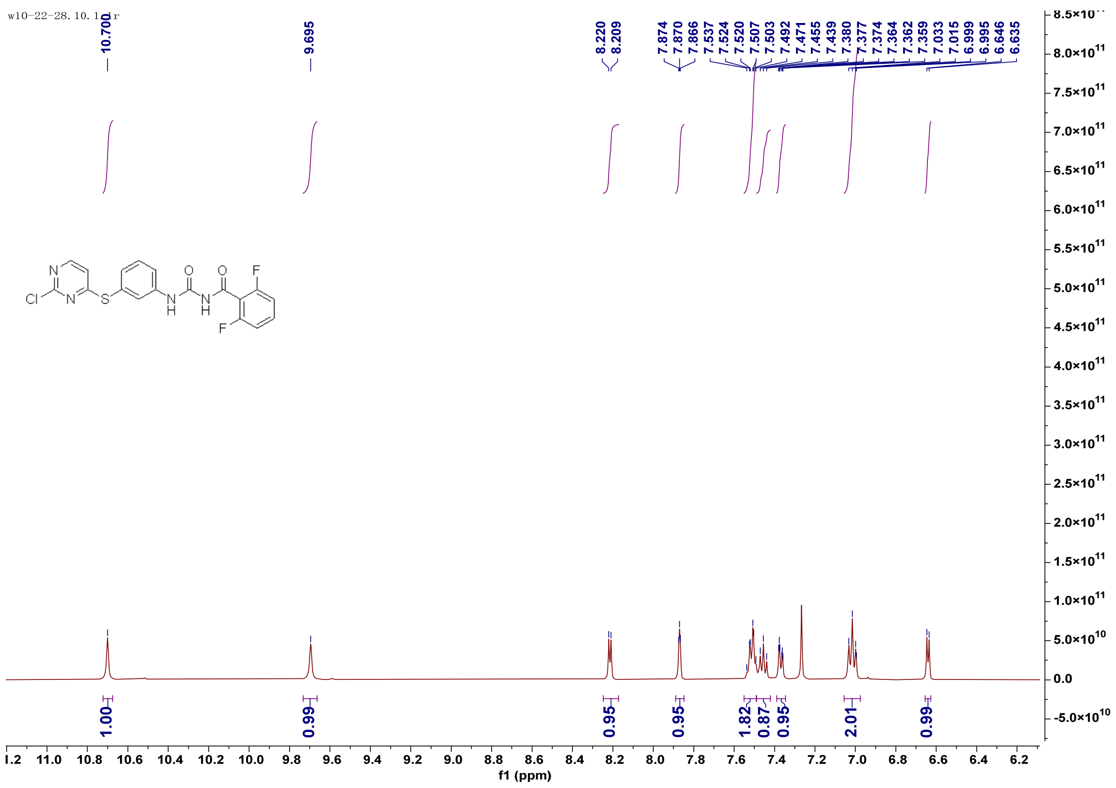

Figure S31 ^{1}H NMR spectrum of compound **4k**

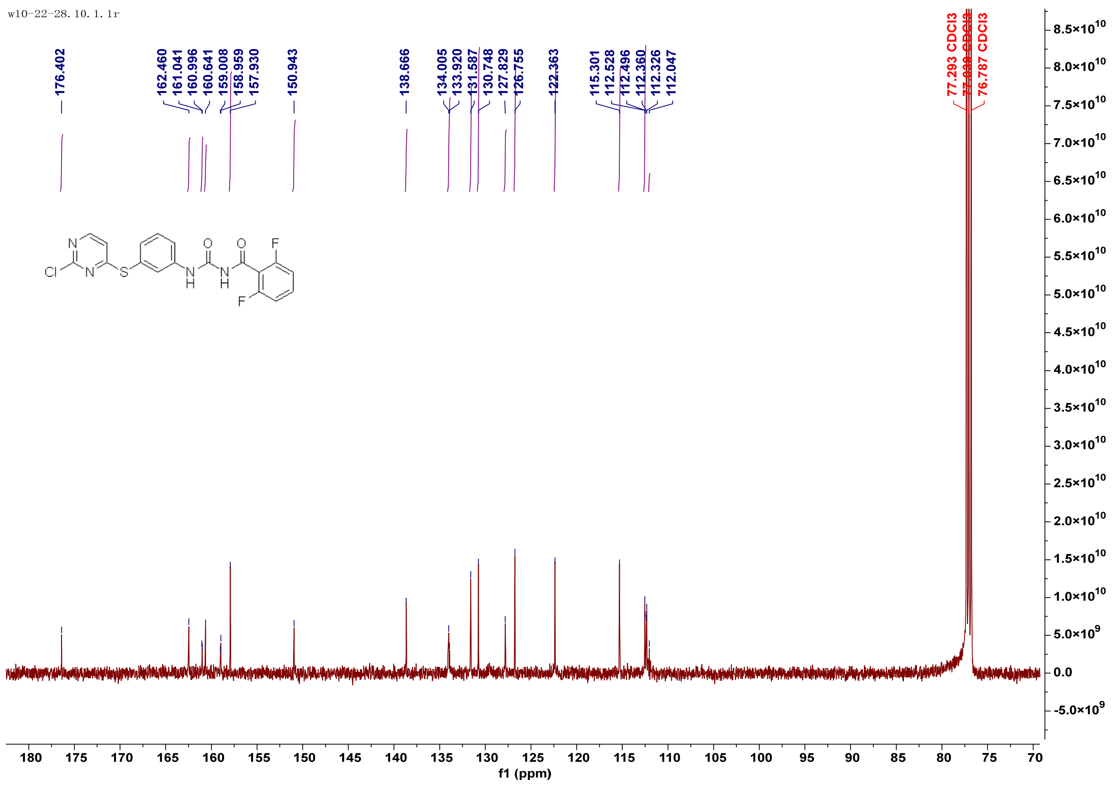

Figure S32 ^{13}C NMR spectrum of compound **4k**

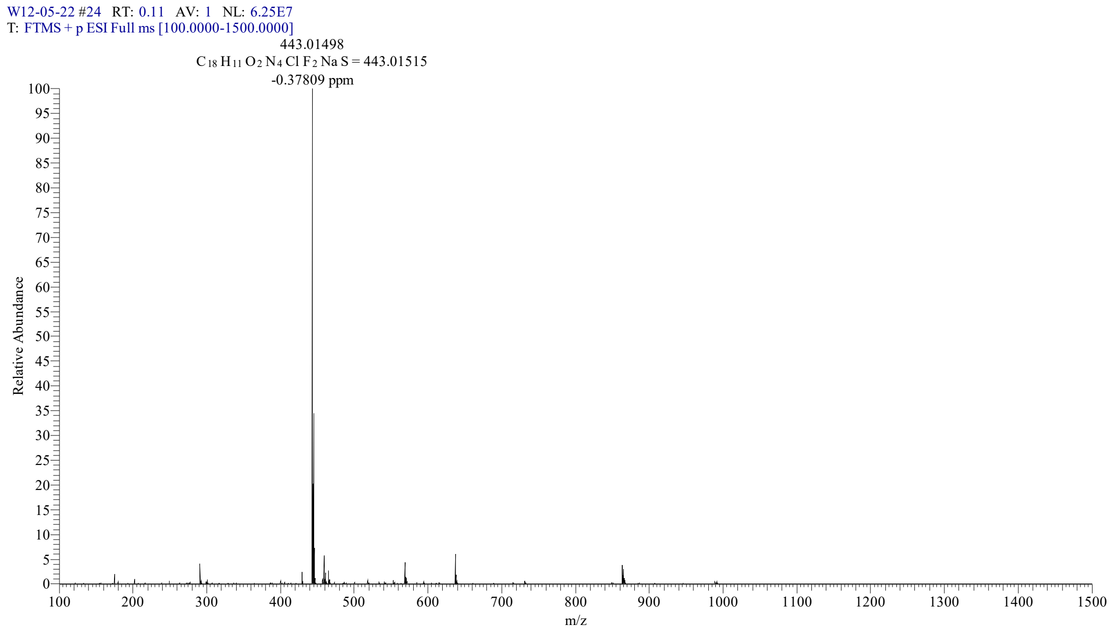

Figure S33 HRMS spectrum of compound **4k**

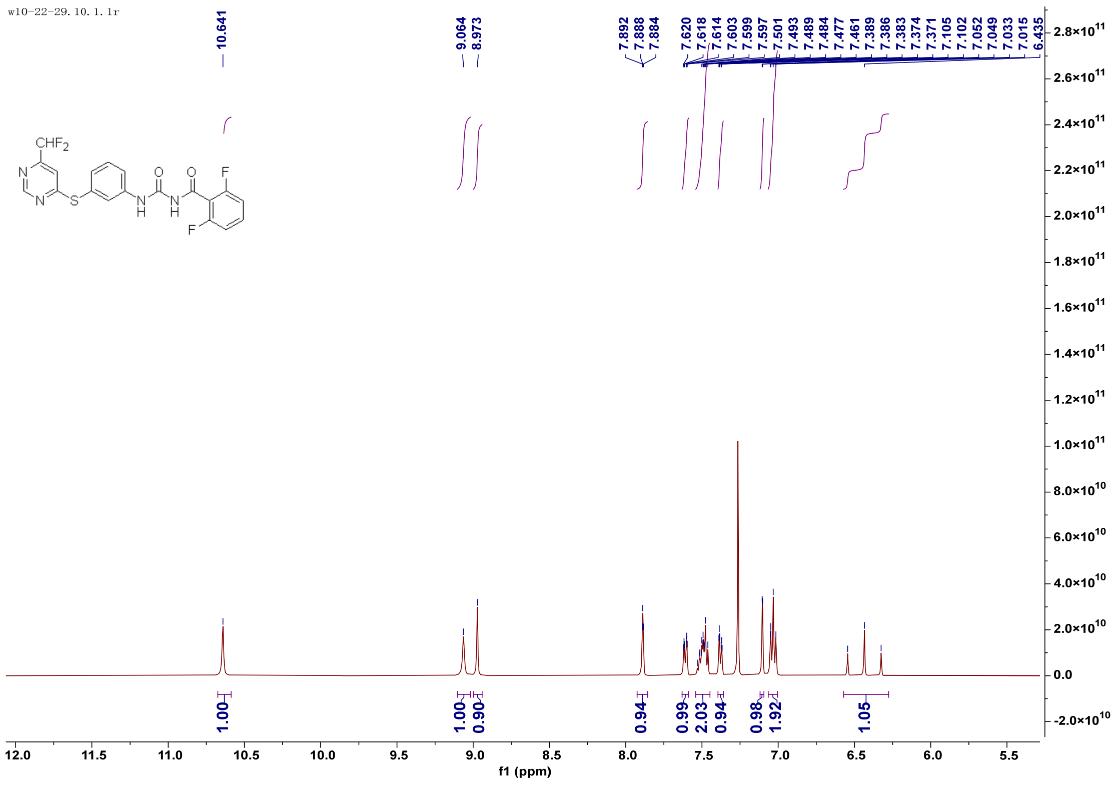

Figure S34 ^{1}H NMR spectrum of compound **4l**

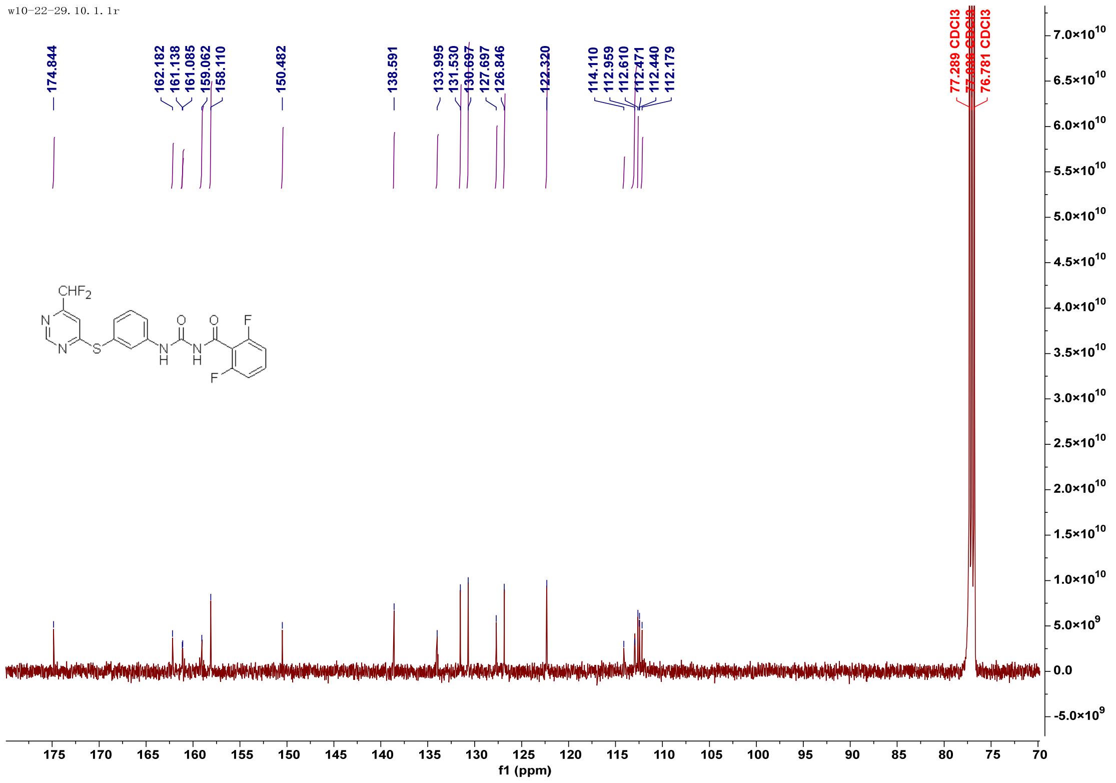

Figure S35 ^{13}C NMR spectrum of compound **4l**

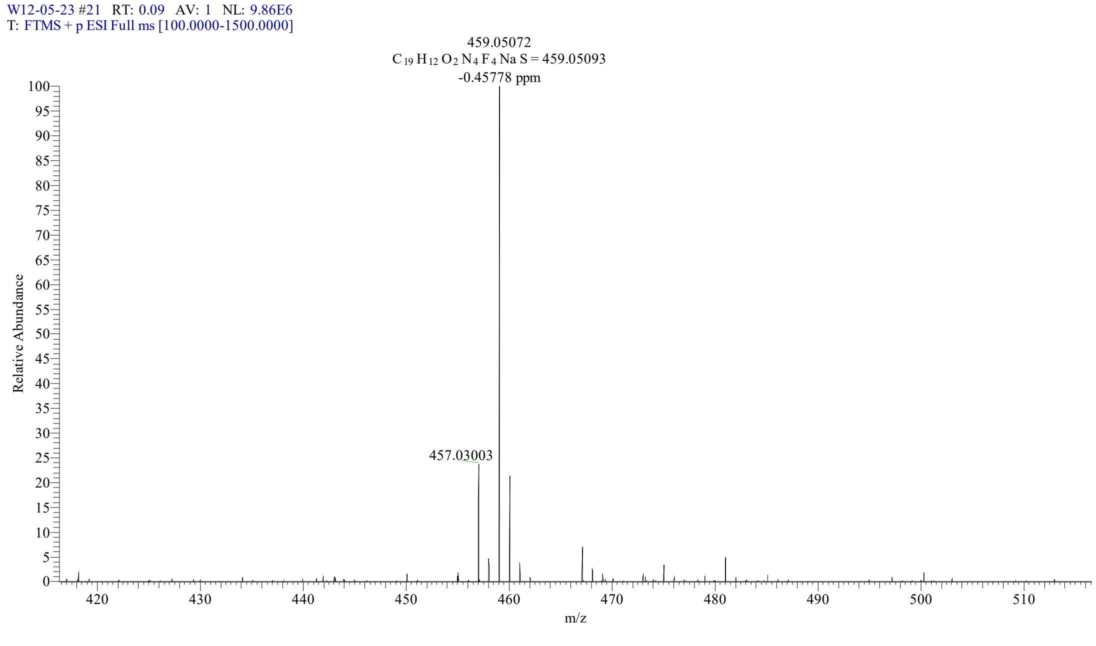

Figure S36 HRMS spectrum of compound **4l**

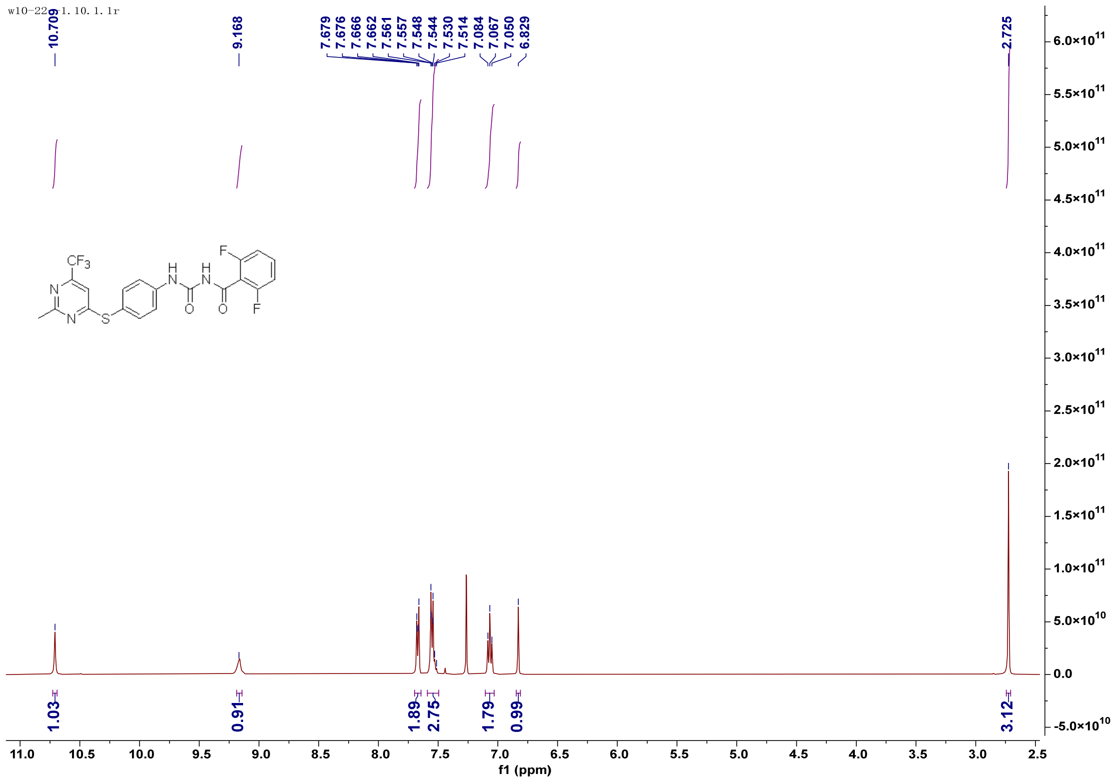

Figure S37 ^{1}H NMR spectrum of compound **4m**

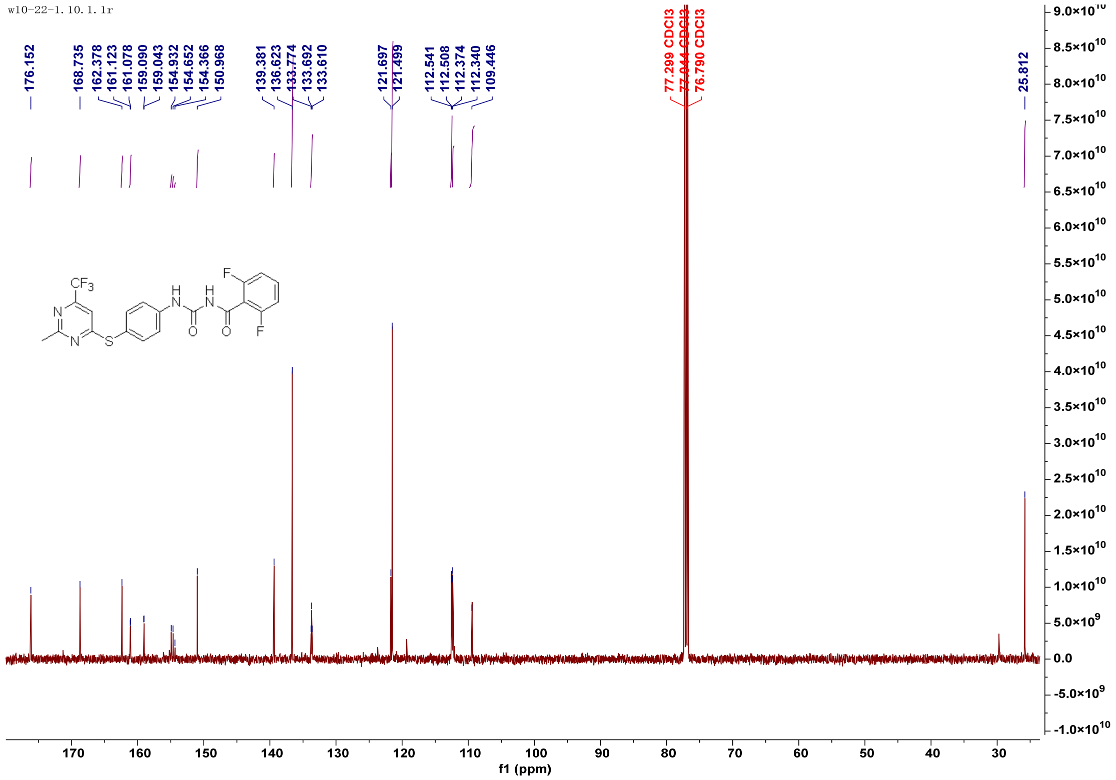

Figure S38 ^{13}C NMR spectrum of compound **4m**

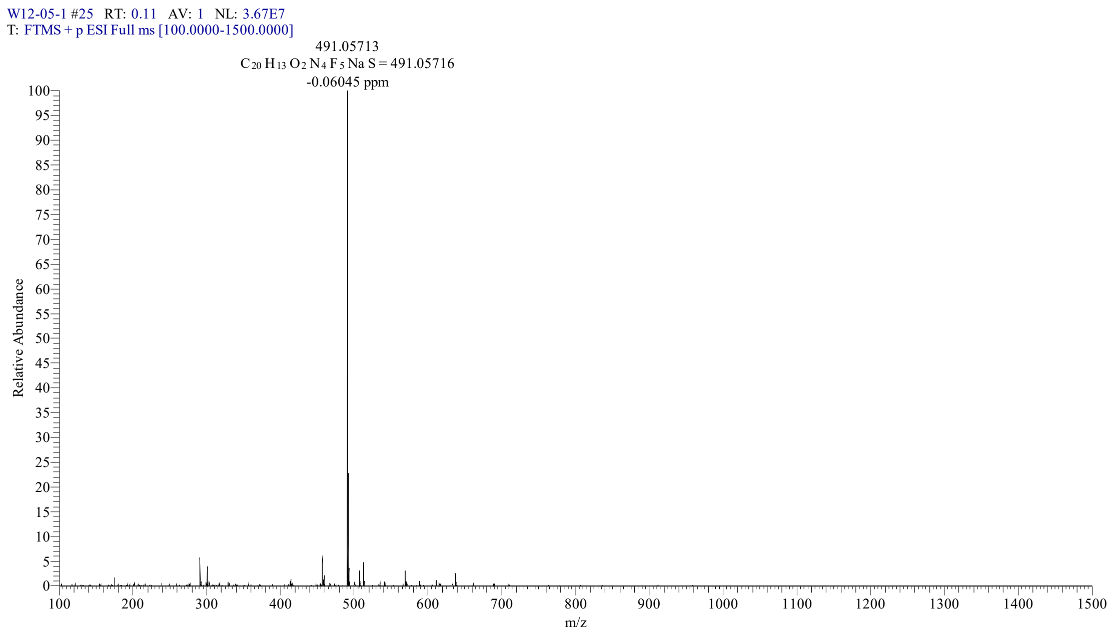

Figure S39 HRMS spectrum of compound **4m**

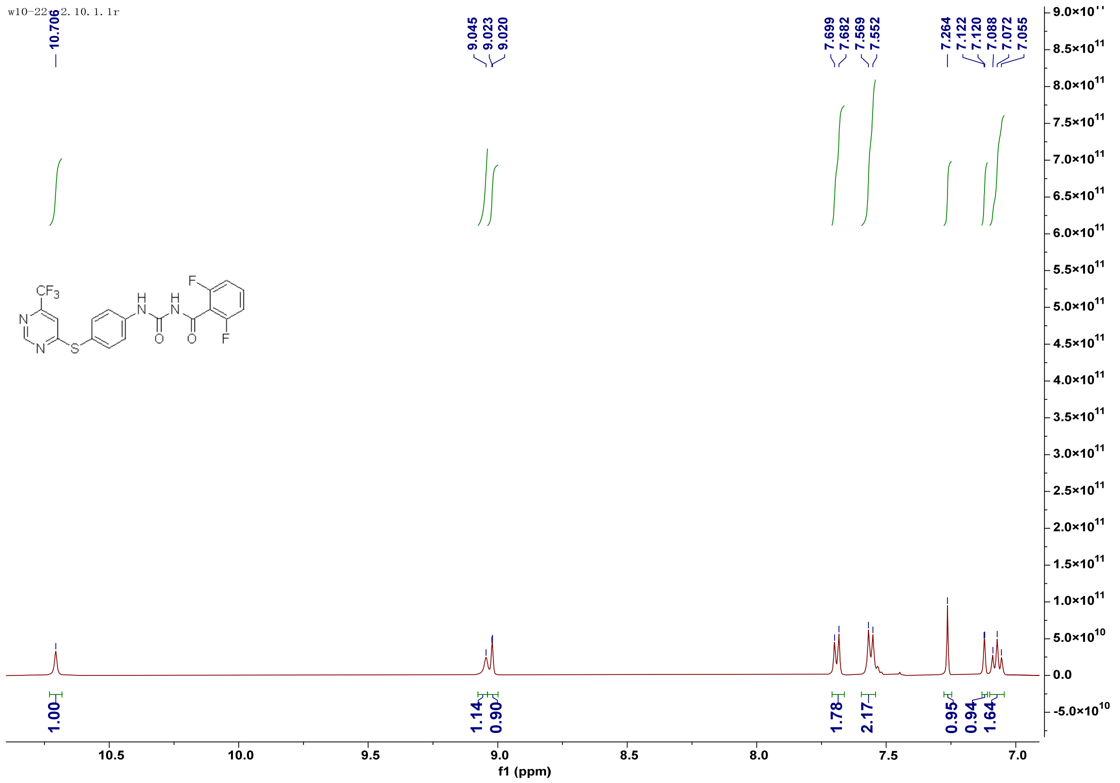

Figure S40 ^{1}H NMR spectrum of compound **4n**

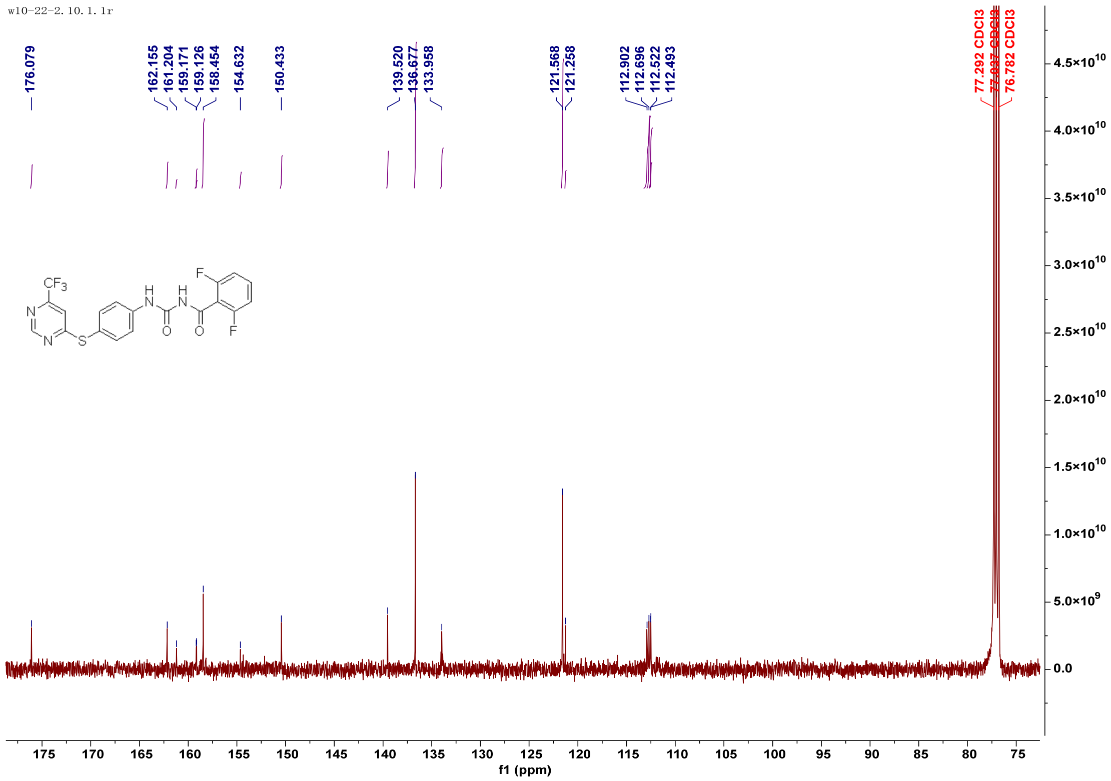

Figure S41 ^{13}C NMR spectrum of compound **4n**

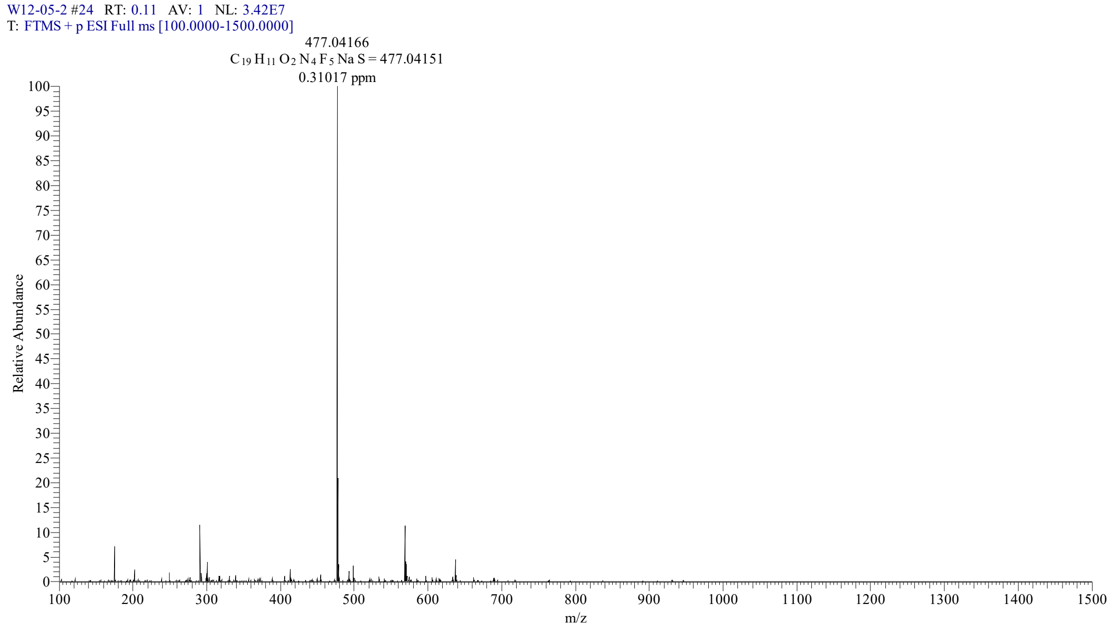

Figure S42 HRMS spectrum of compound **4n**

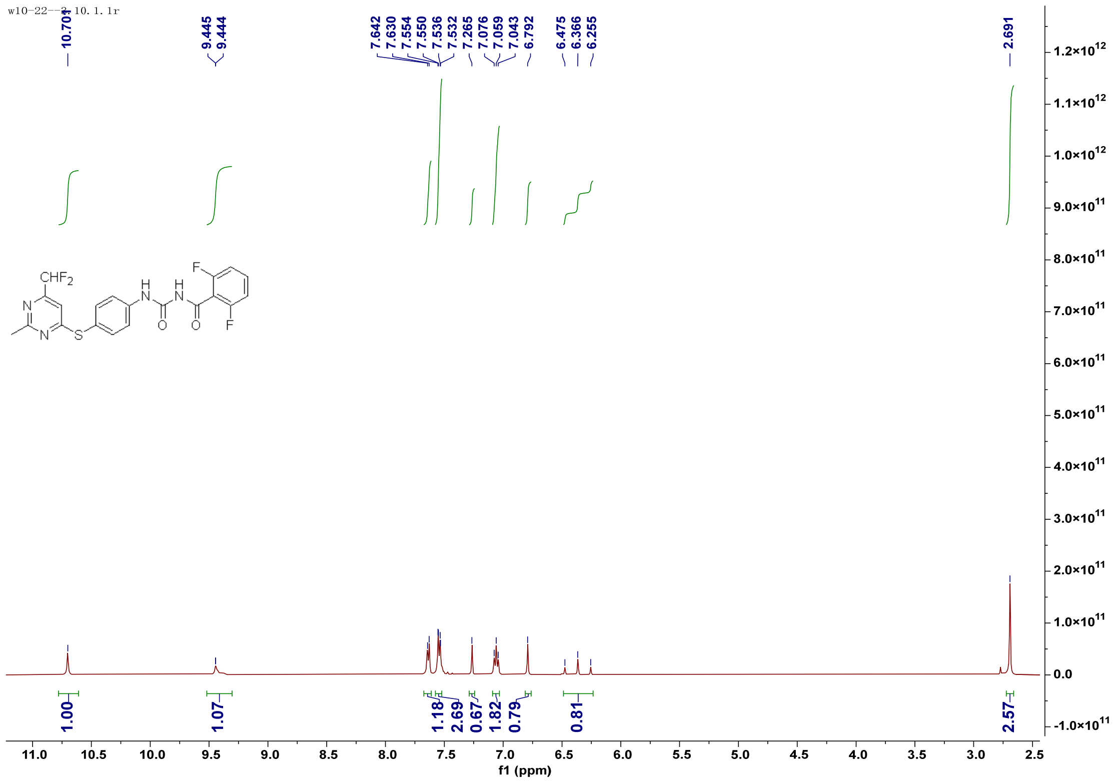

Figure S43 ^{1}H NMR spectrum of compound **4o**

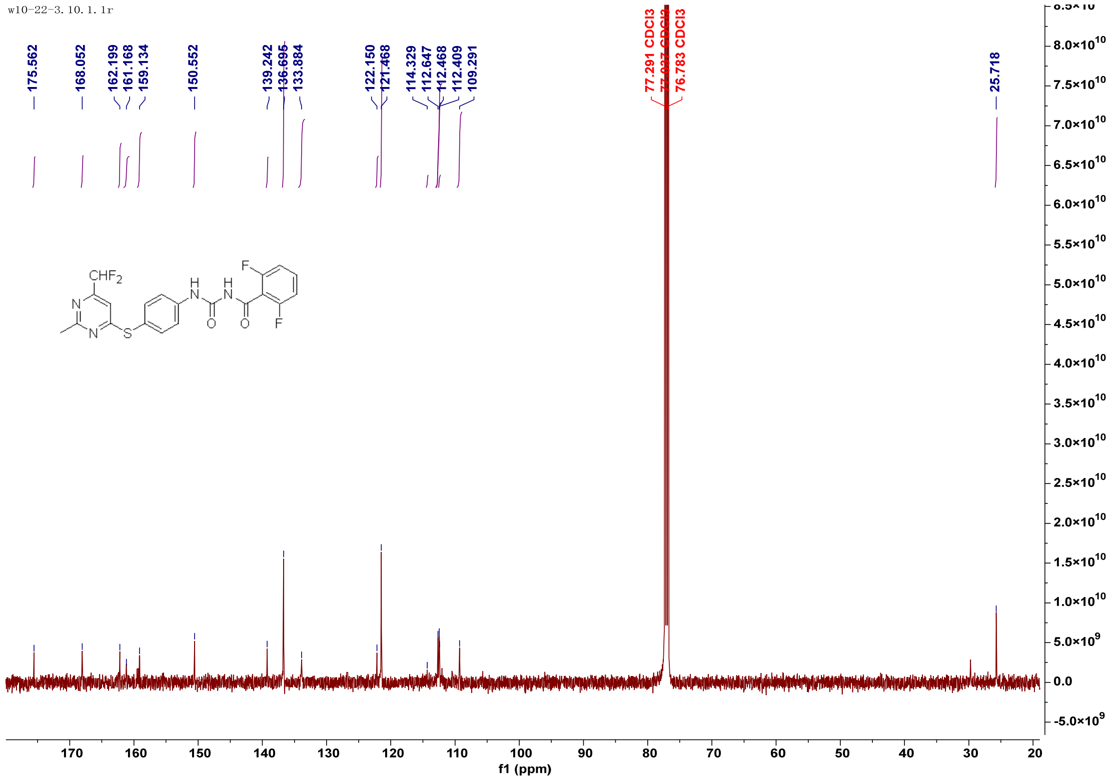

Figure S44 ^{13}C NMR spectrum of compound **4o**

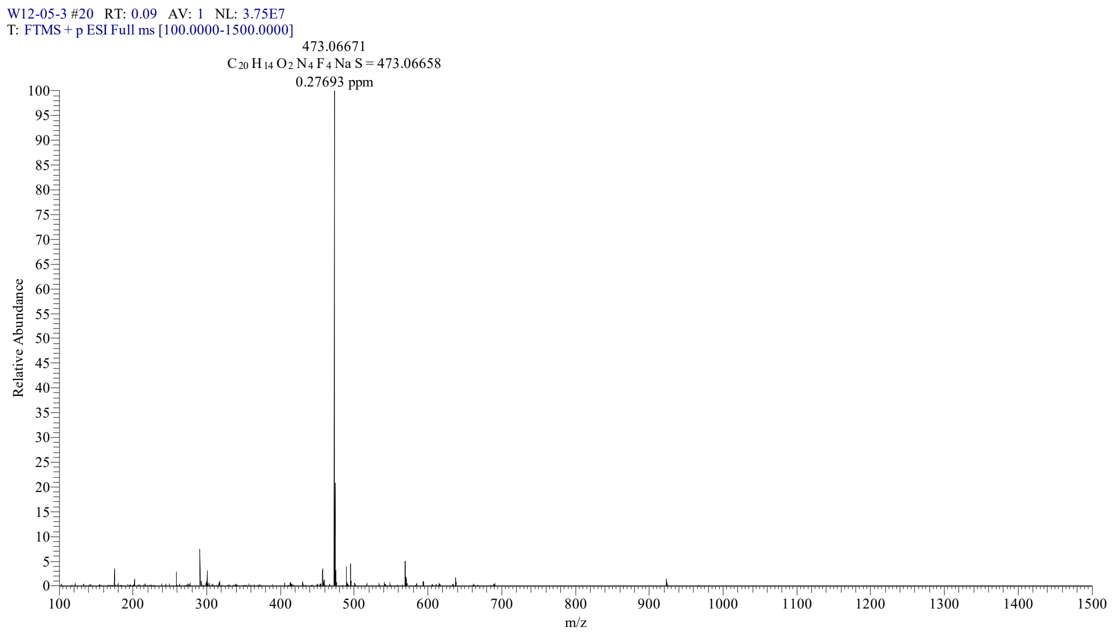

Figure S45 HRMS spectrum of compound **4o**

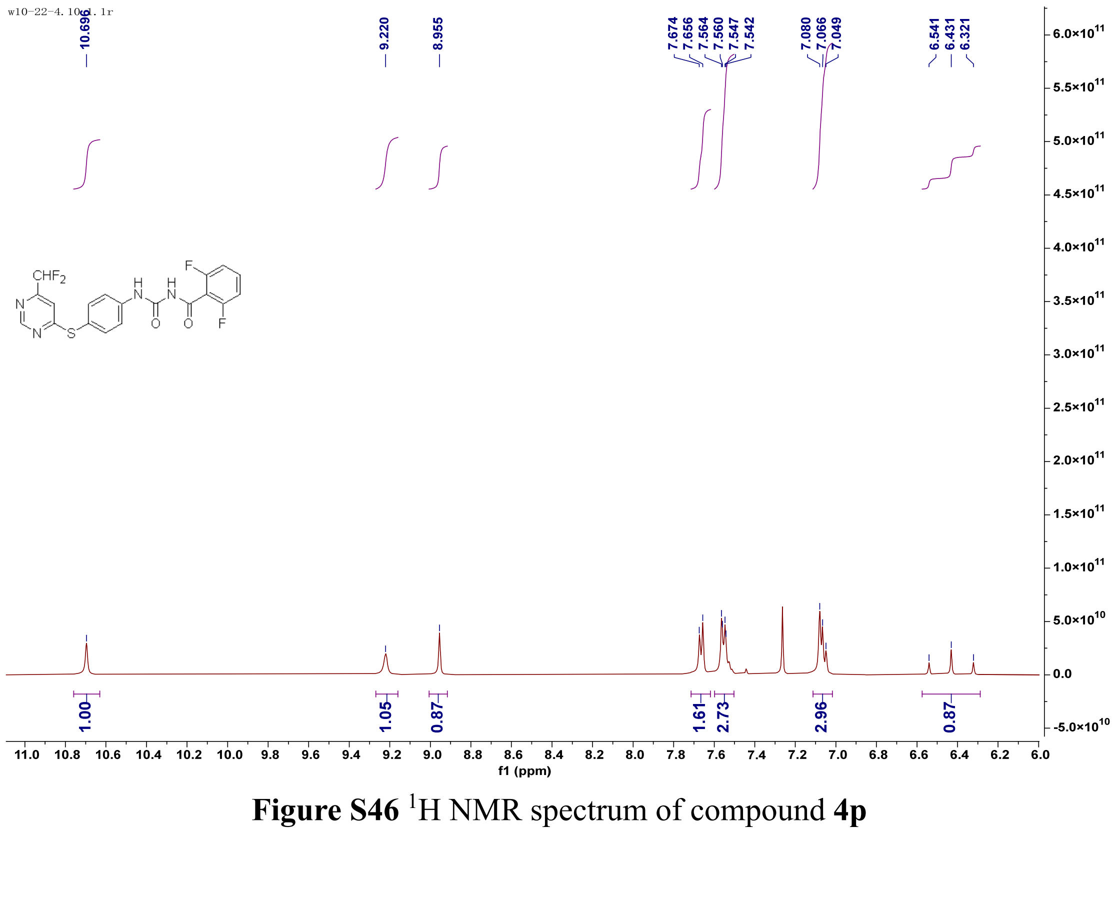

Figure S46 ^{1}H NMR spectrum of compound **4p**

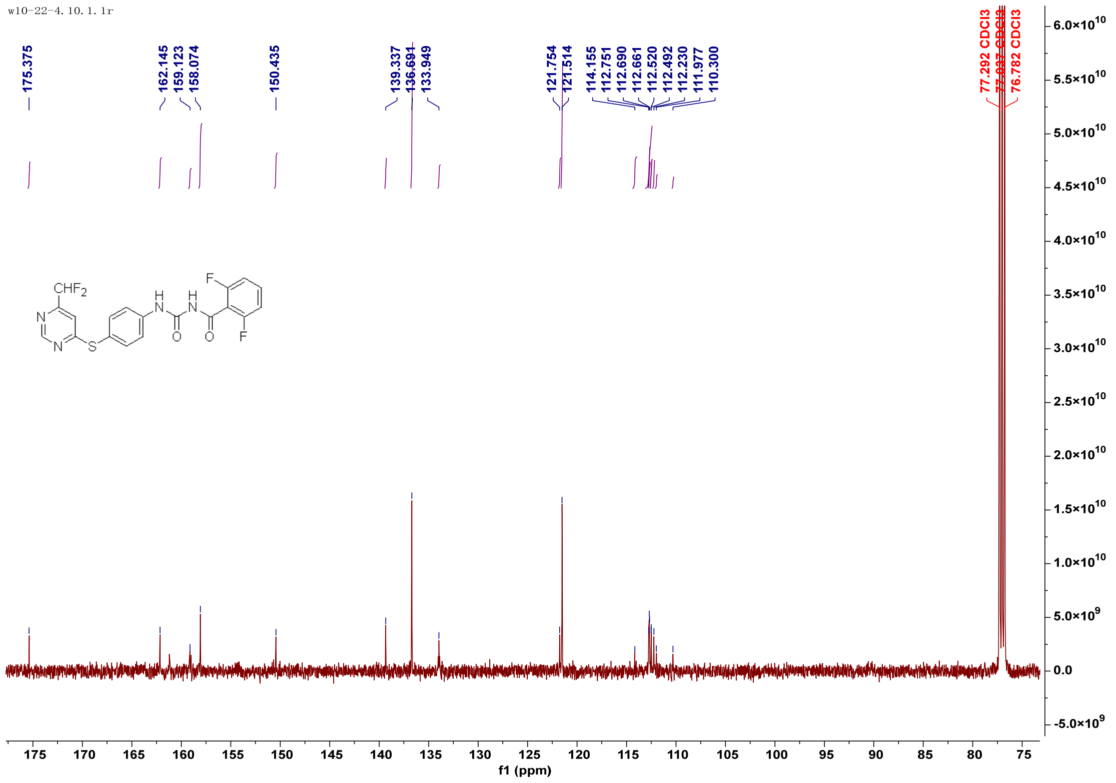

Figure S47 ^{13}C NMR spectrum of compound **4p**

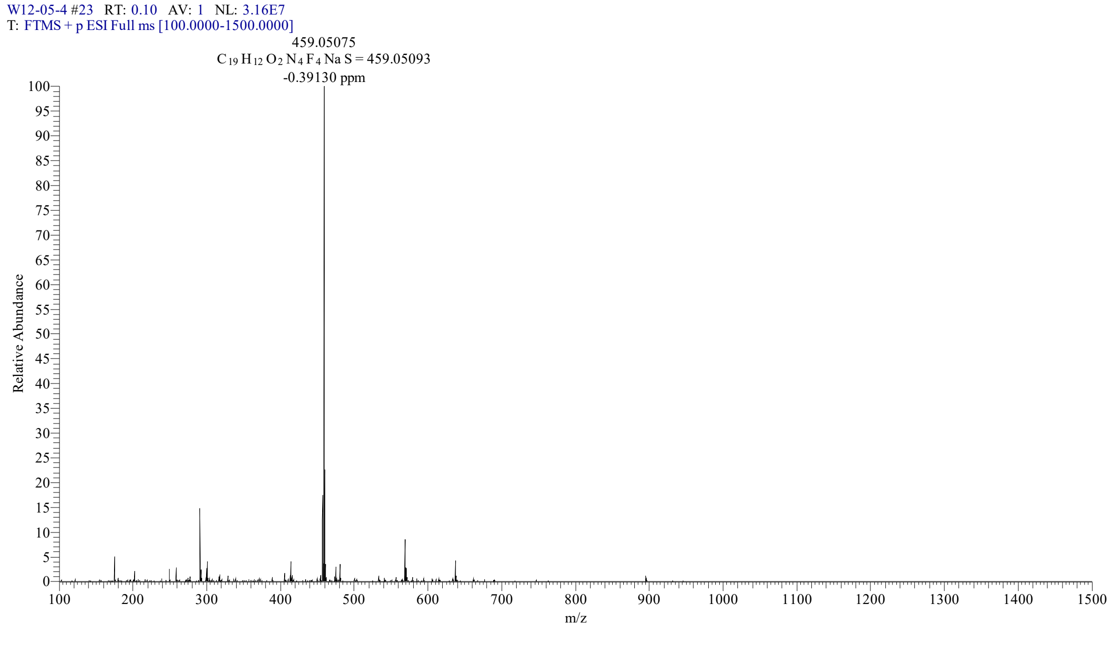

Figure S48 HRMS spectrum of compound **4p**

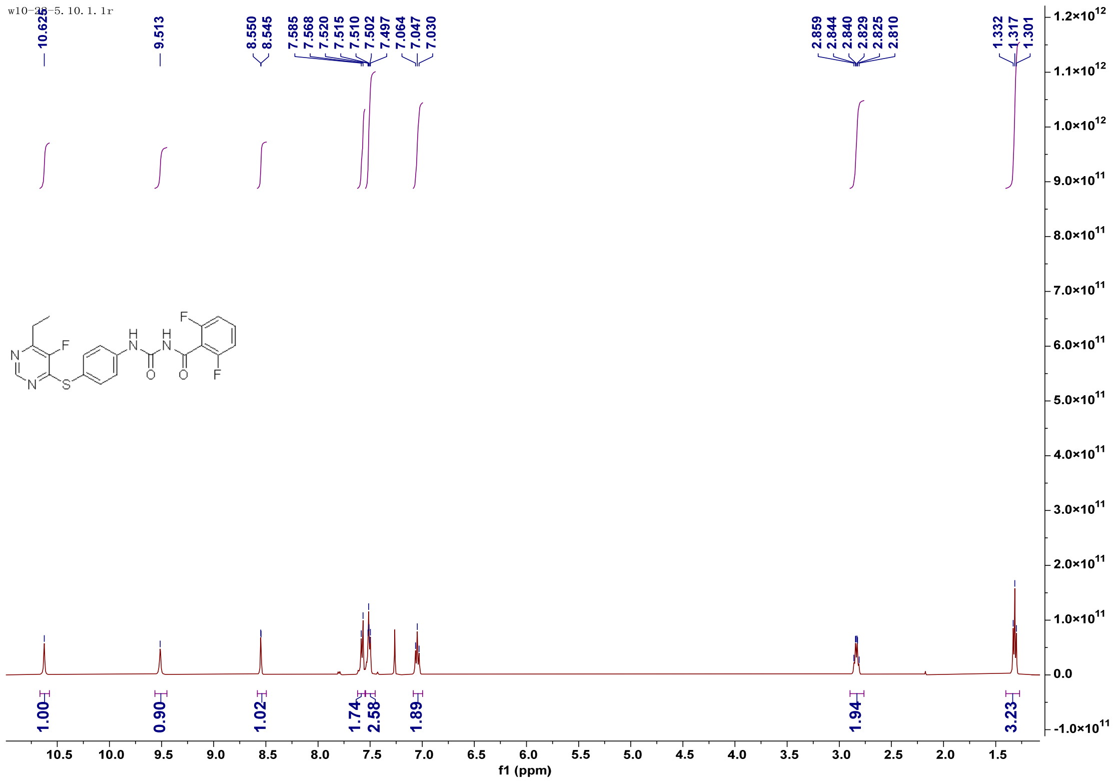

Figure S49 ^{1}H NMR spectrum of compound **4q**

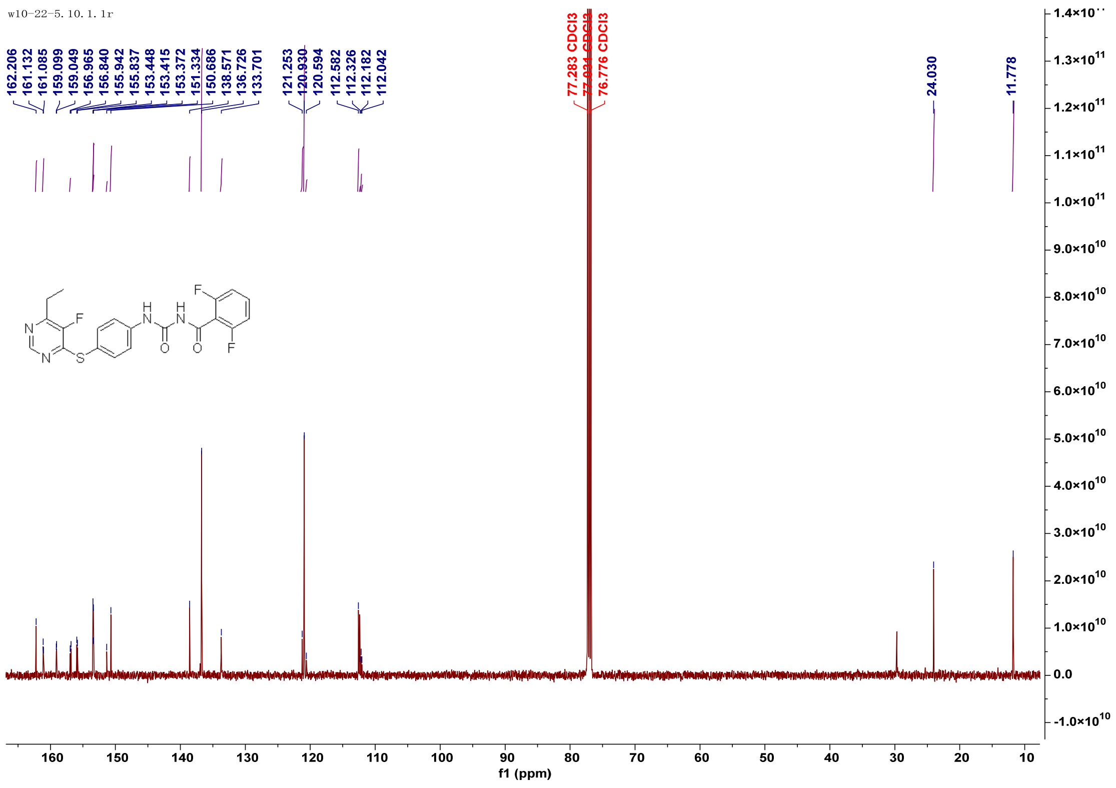

Figure S50 ^{13}C NMR spectrum of compound **4q**

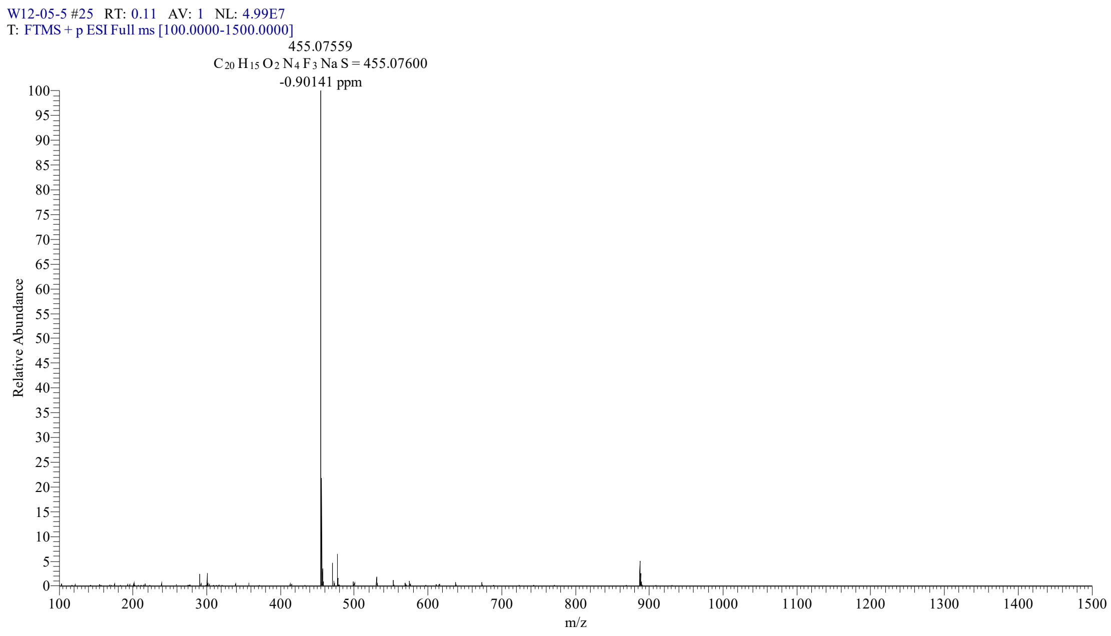

Figure S51 HRMS spectrum of compound **4q**

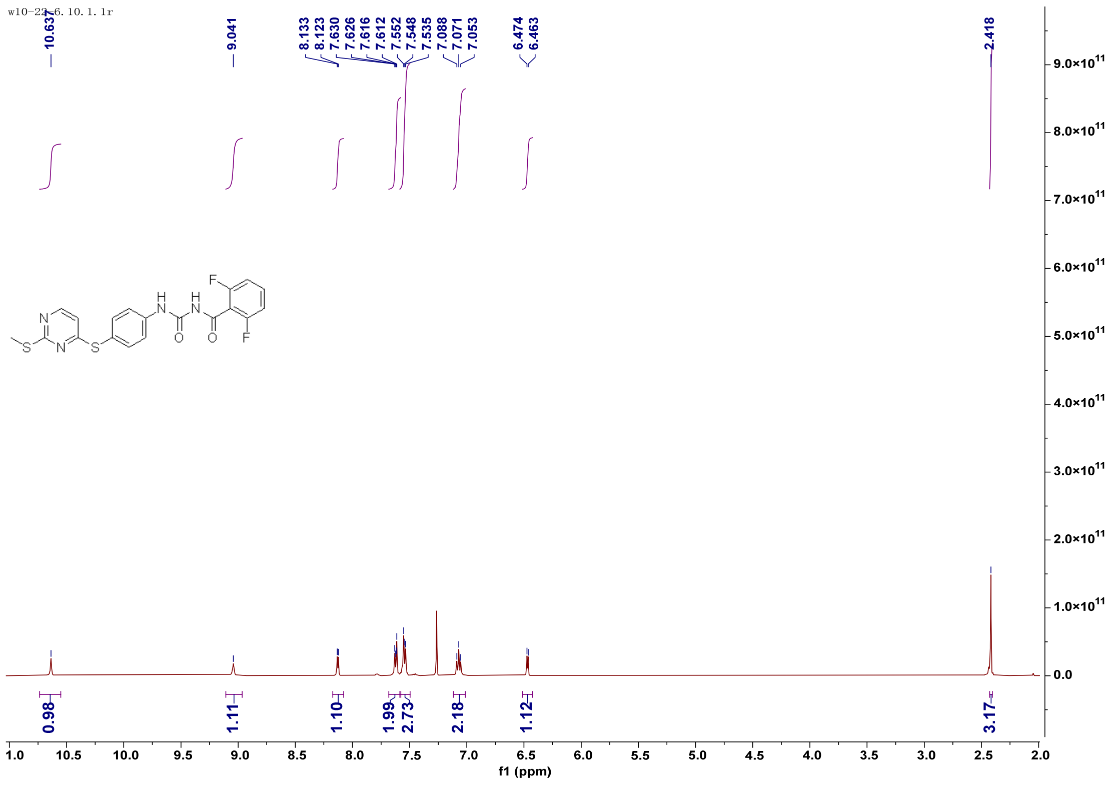

Figure S52 ^{1}H NMR spectrum of compound **4r**

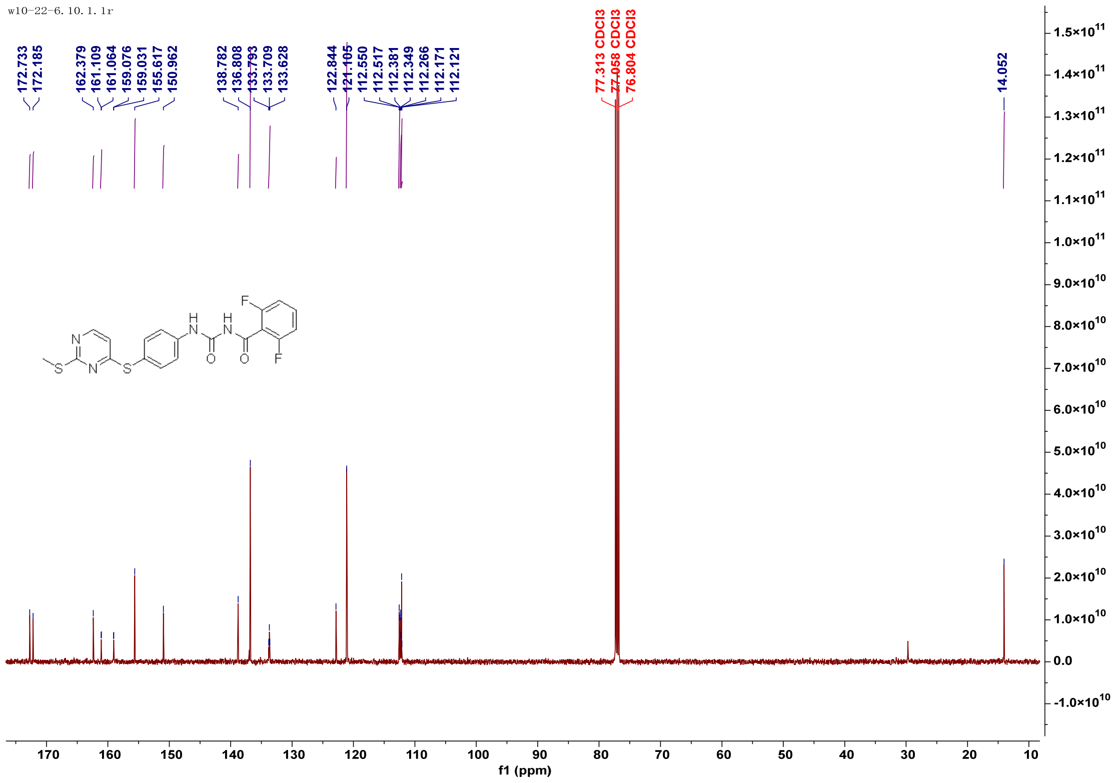

Figure S53 ^{13}C NMR spectrum of compound **4r**

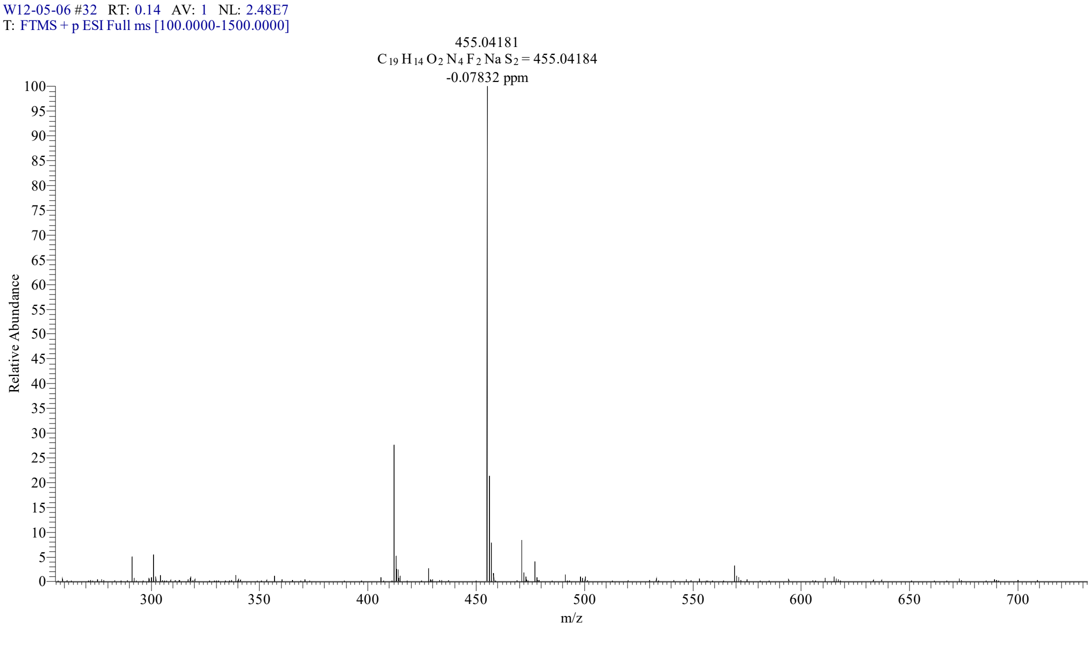

Figure S54 HRMS spectrum of compound **4r**

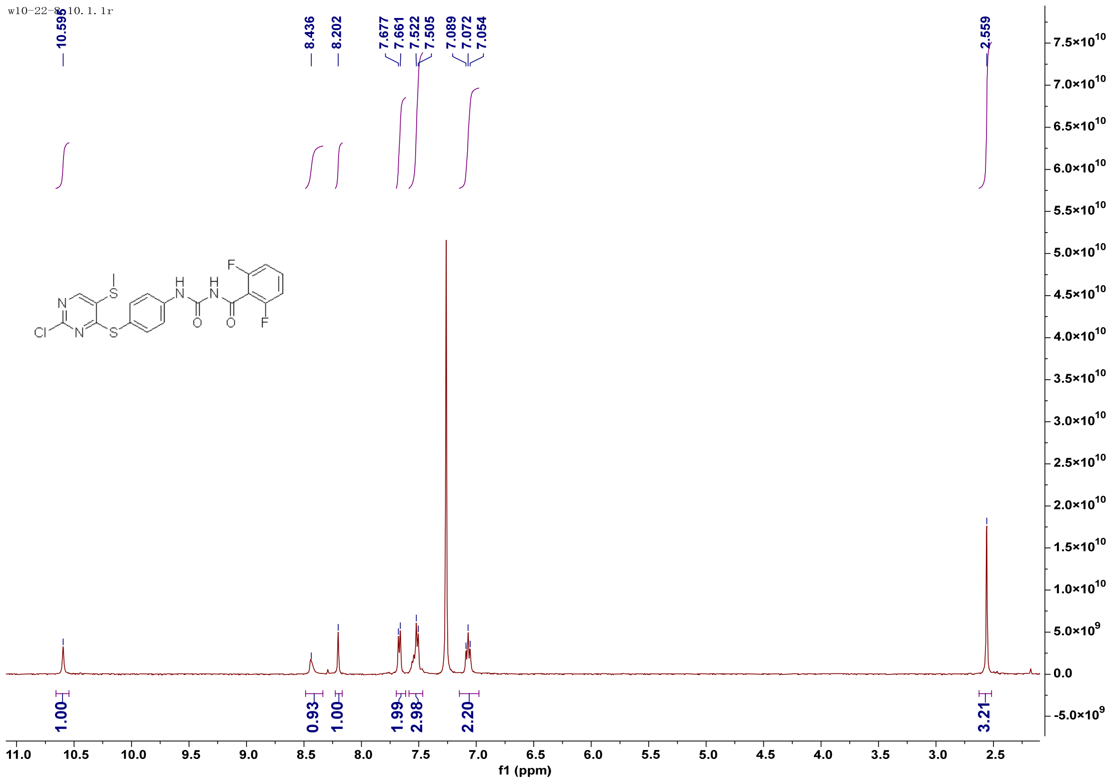

Figure S55 ^{1}H NMR spectrum of compound **4s**

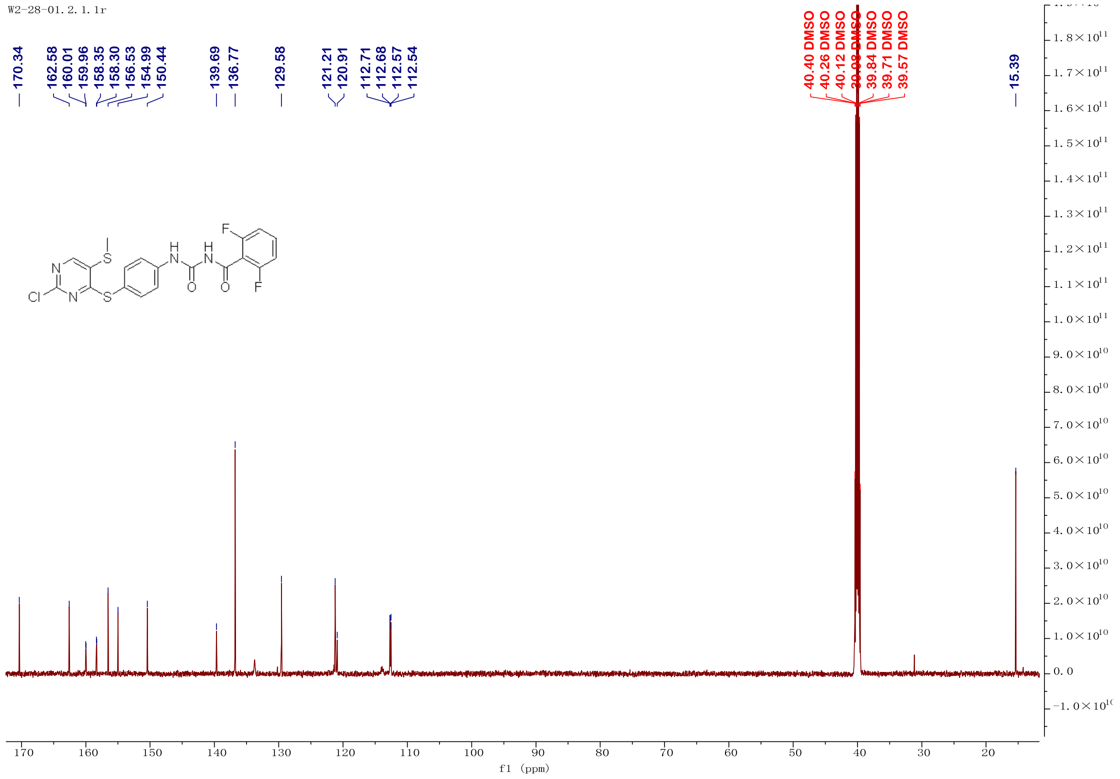

Figure S56 ^{13}C NMR spectrum of compound **4s**

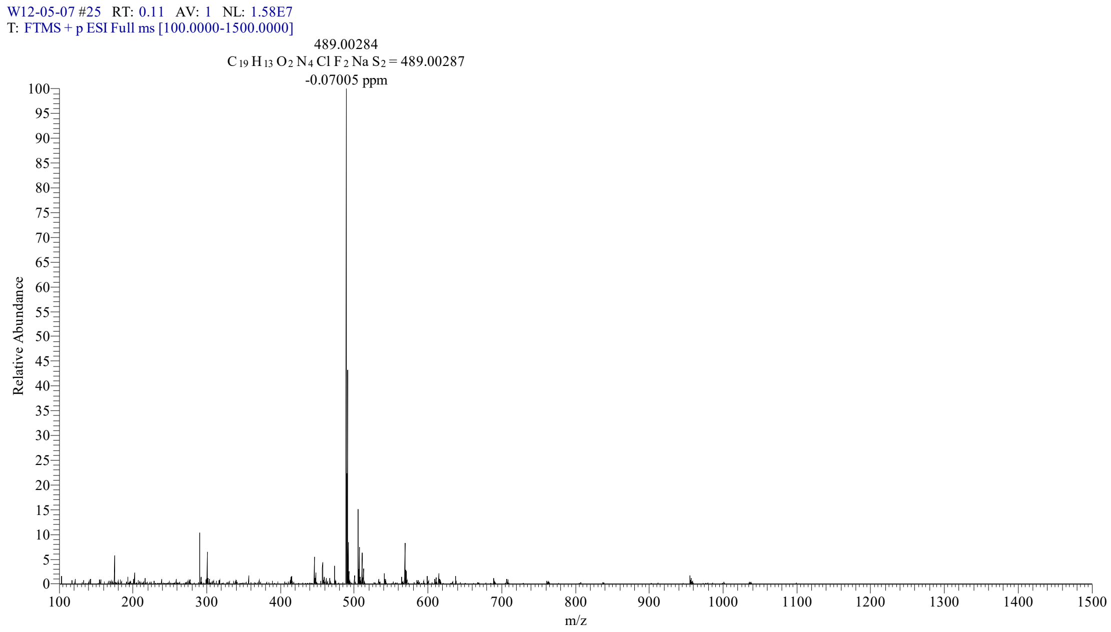

Figure S57 HRMS spectrum of compound **4s**

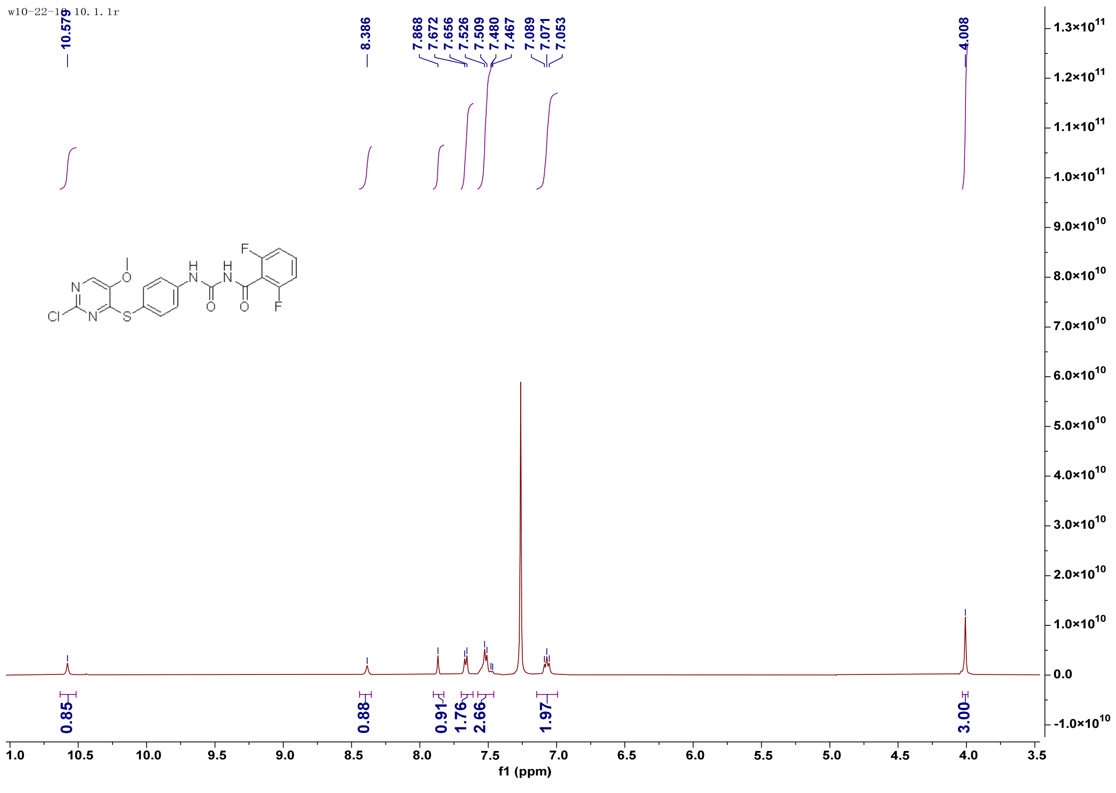

Figure S58 ^{1}H NMR spectrum of compound **4t**

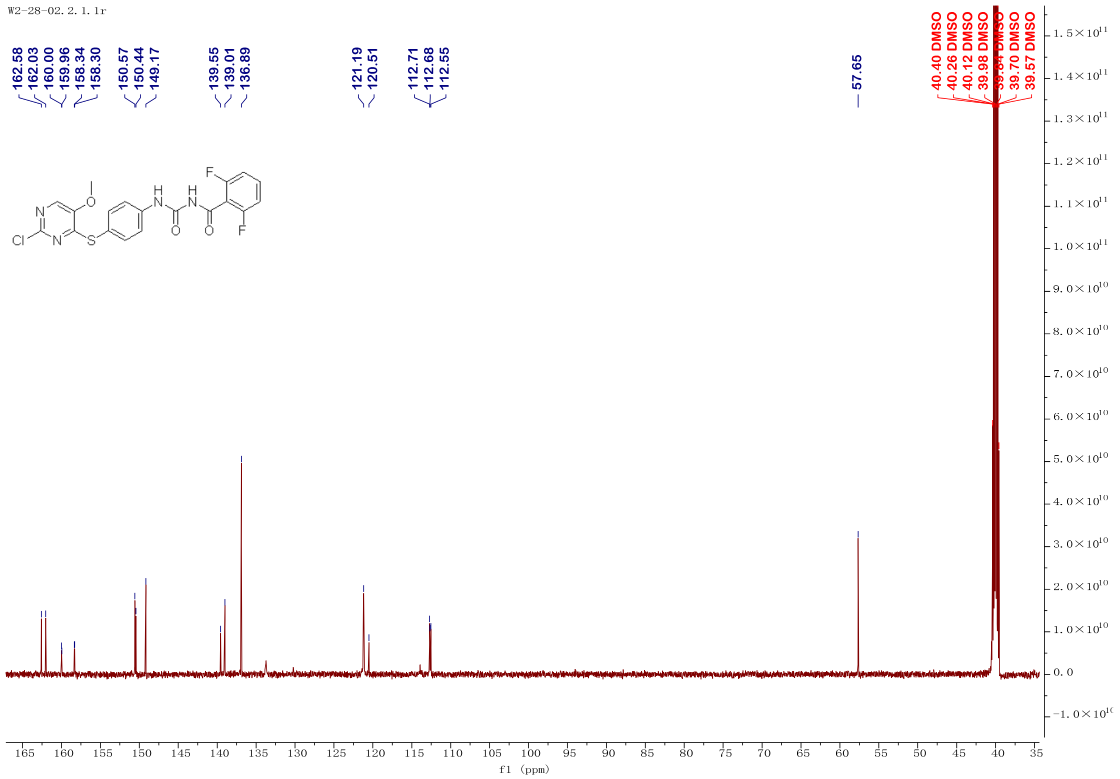

Figure S59 ^{13}C NMR spectrum of compound **4t**

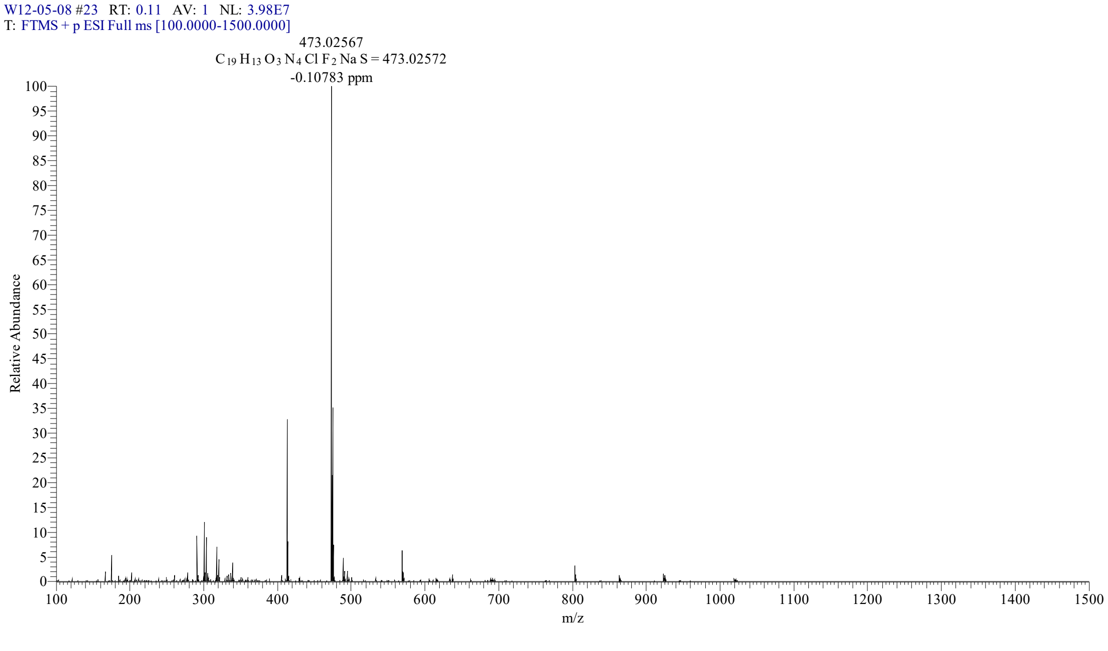

Figure S60 HRMS spectrum of compound **4t**

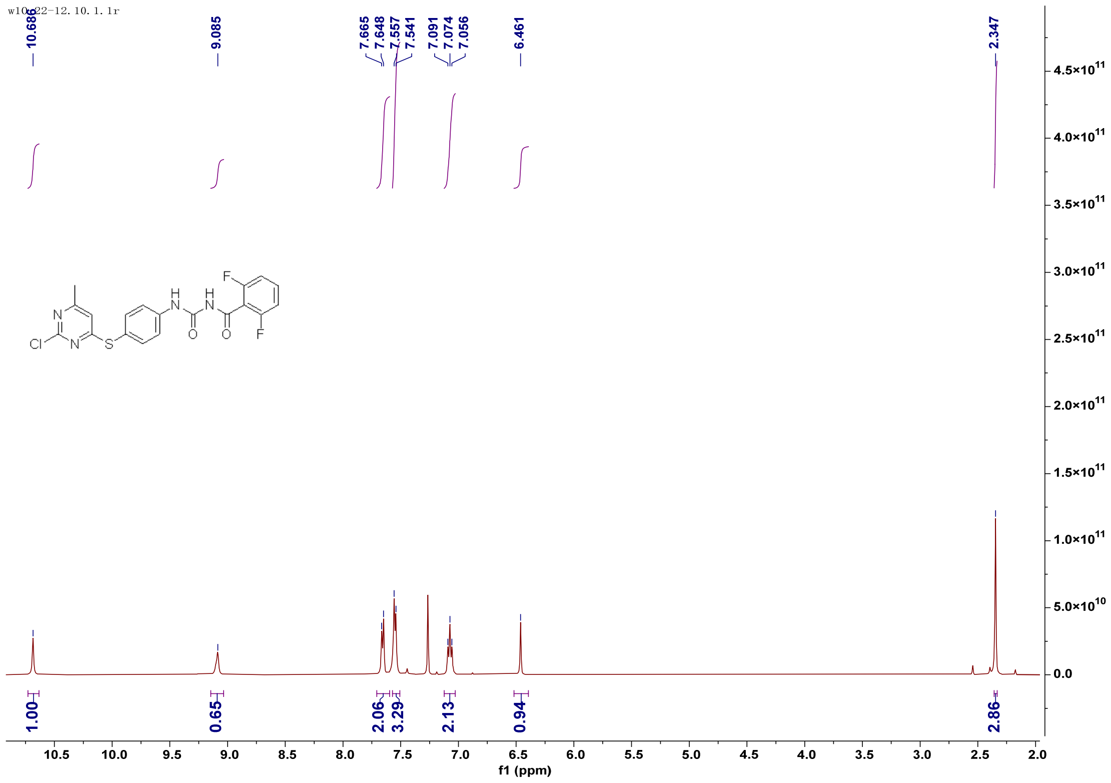

Figure S61 ^{1}H NMR spectrum of compound **4u**

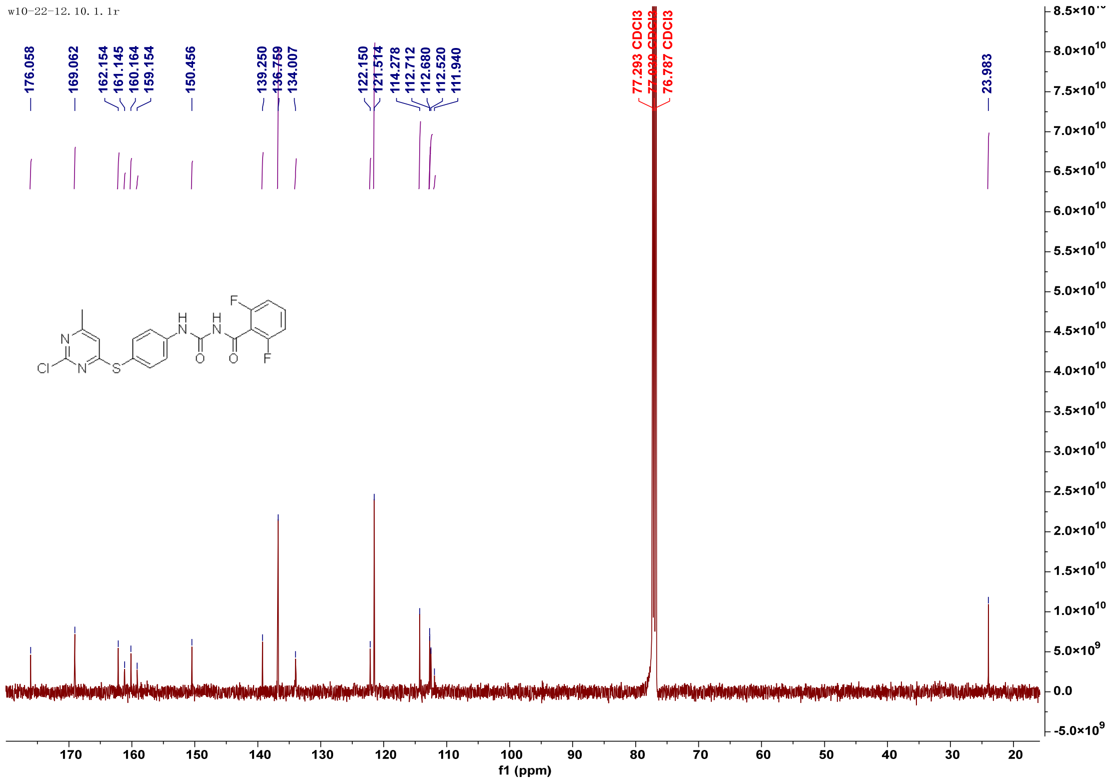

Figure S62 ^{13}C NMR spectrum of compound **4u**

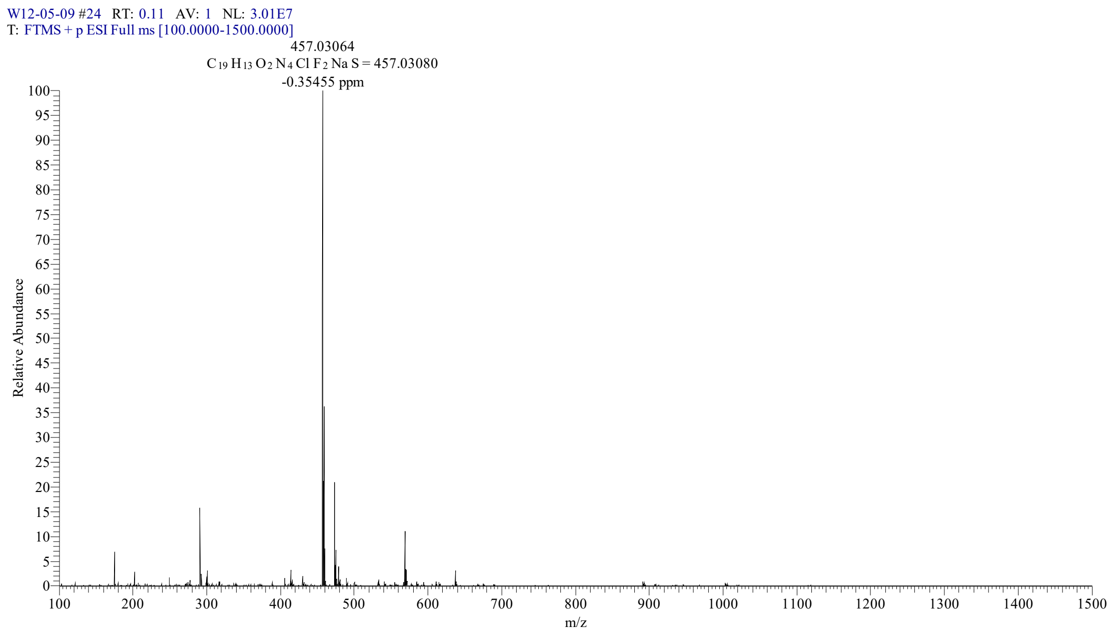

Figure S63 HRMS spectrum of compound **4u**

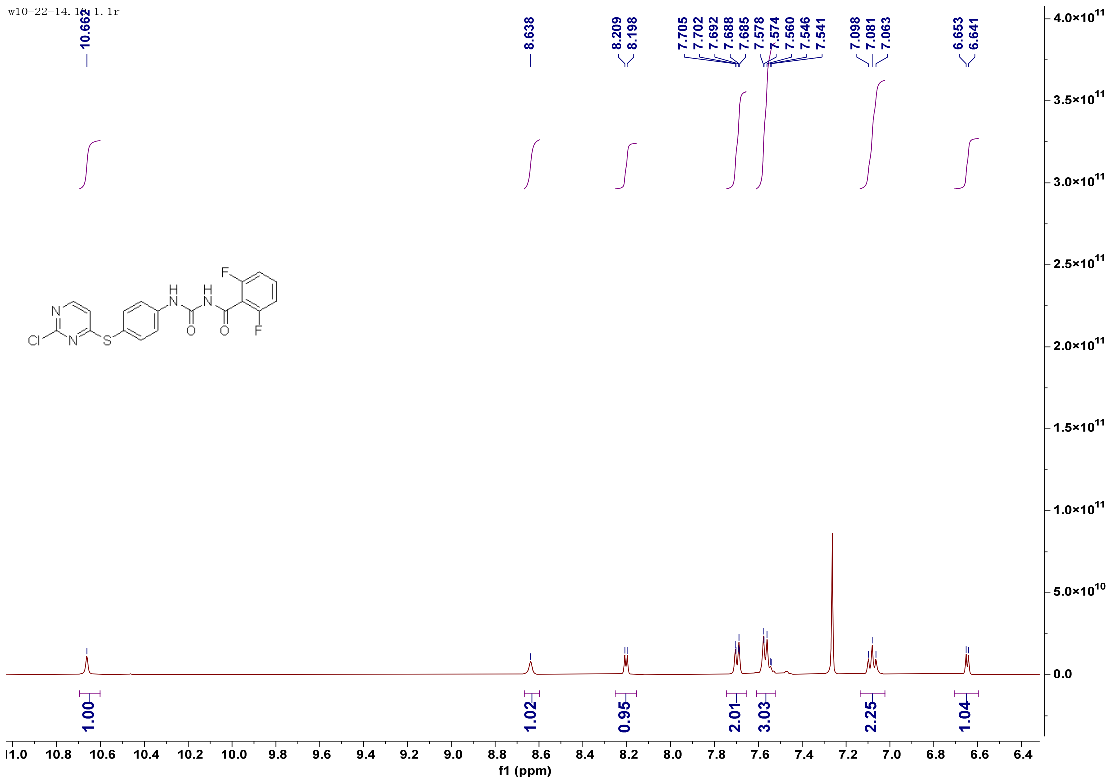

Figure S64 ^{1}H NMR spectrum of compound **4v**

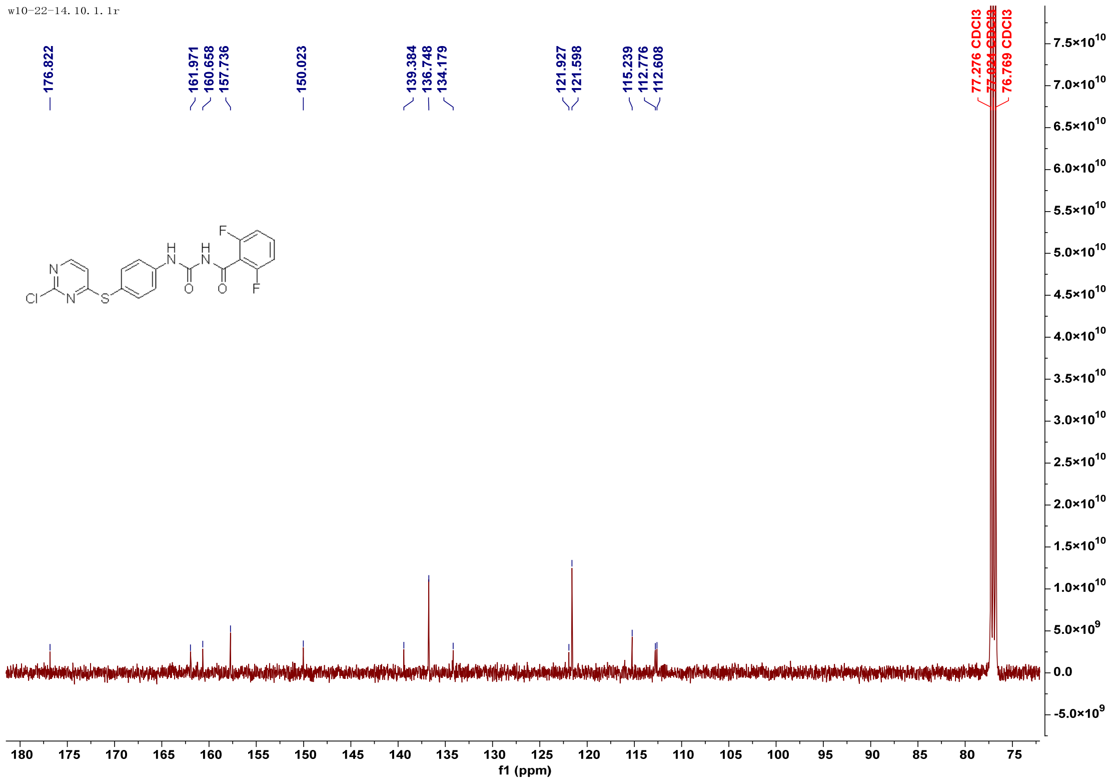

Figure S65 ^{13}C NMR spectrum of compound **4v**

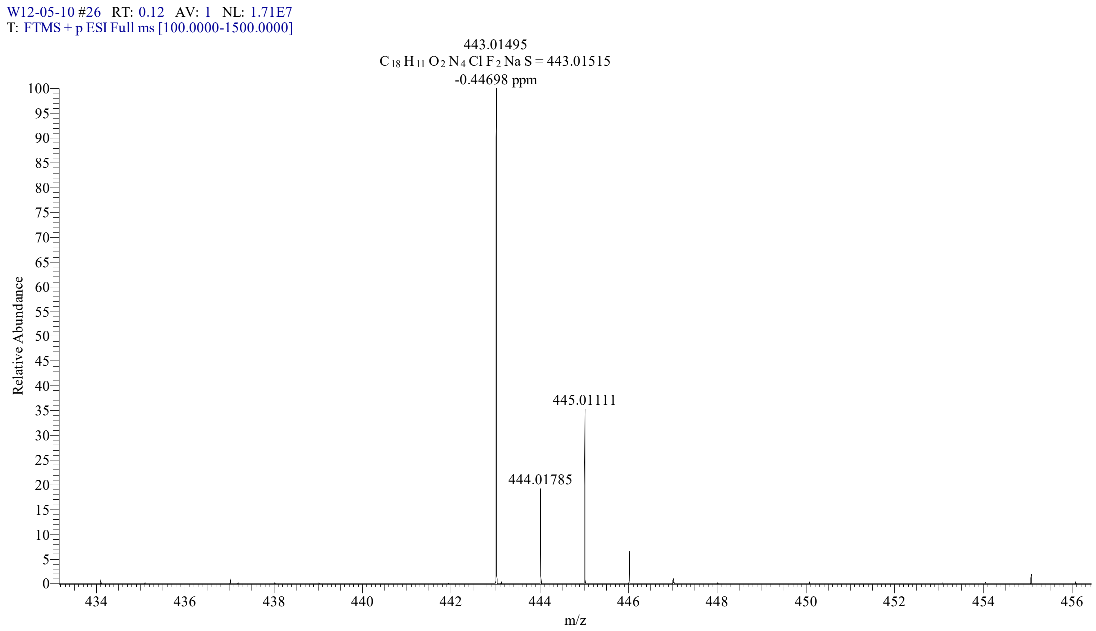

Figure S66 HRMS spectrum of compound **4v**

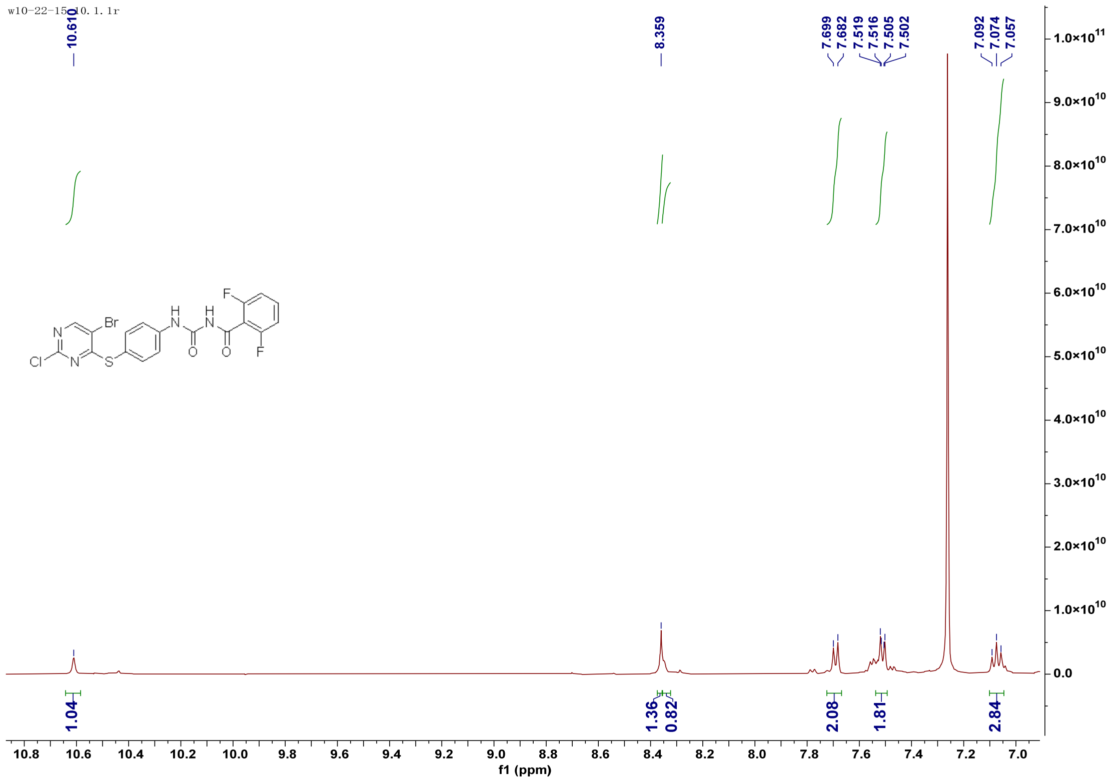

Figure S67 ^{1}H NMR spectrum of compound **4w**

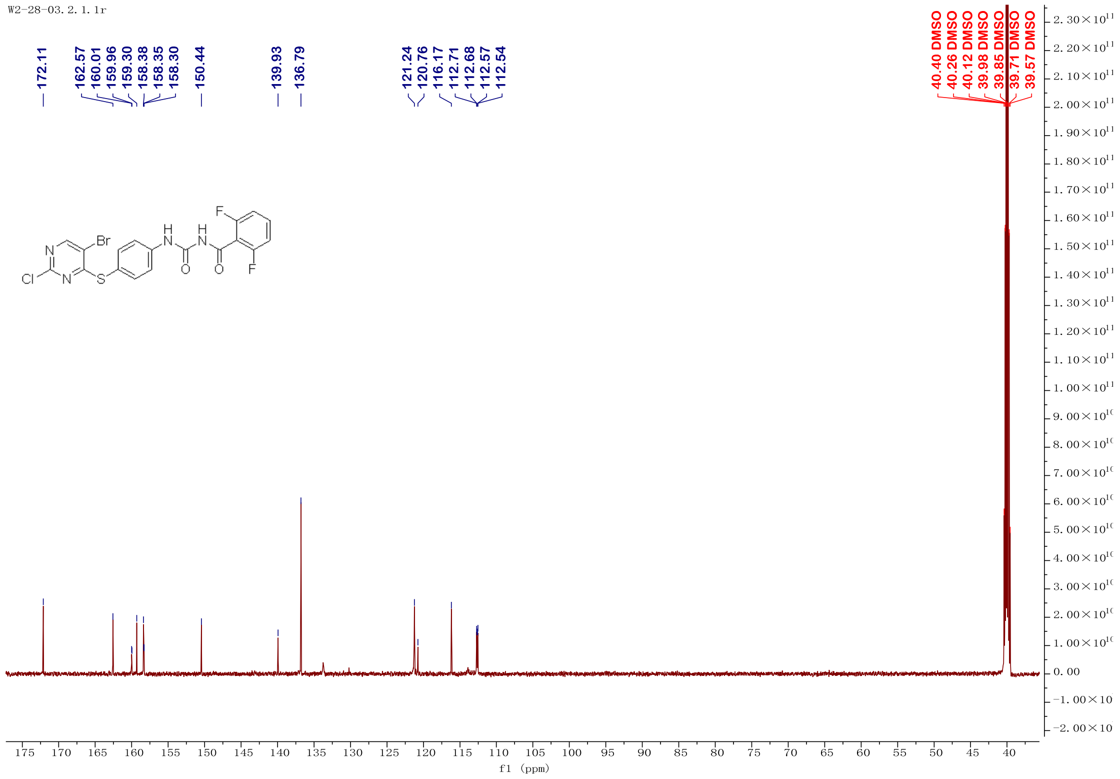

Figure S68 ^{13}C NMR spectrum of compound **4w**

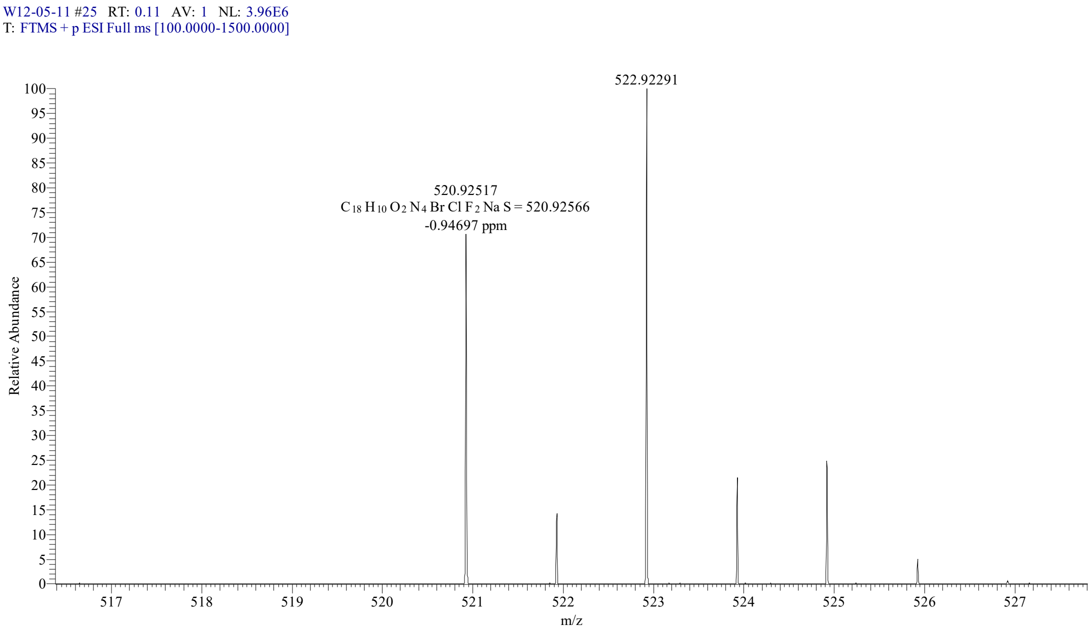

Figure S69 HRMS spectrum of compound **4w**
